# Supplementary material for: Navigating challenges: a socioecological analysis of sexual and reproductive health barriers among Eritrean refugee women in Ethiopia, using a key informant approach
Source: BMJ Open. 2024 Apr 23;14(4):e080654. doi: 10.1136/bmjopen-2023-080654 (PMC11043775; doi:10.1136/bmjopen-2023-080654)
Supplement: Supplementary data [file bmjopen-2023-080654supp002.pdf]

NIIDS
V2.0_09.02.2021

KII with health professionals and Key officers

Health professionals in the refugee camp/CH: health professionals treating Eritrean/Somali migrants

**Demographic Characteristics of the study participants**
Place
Date
Interviewers

ID	Age	Type of the professional*	Experience in camp/center	Religion	Education level	Nationality

***Medical doctor, Nurse, Public health etc.**

1. Survey of the present health status:

- Is a general clinical assessment conducted for women arriving at the camp/asylum centre? If yes, please indicate which kind (physical/psychological/gynaecological)?Where is the clinical assessment usually conducted? Who is usually present during the examination? Is the clinical examination done by a man or a woman? How many persons are present during a gynecological examination?
- Is an interpreter consulted during assessment? If not, why not?
- Is a professional interpreter available?
- How are the confidentiality and privacy maintained when SRH issues are discussed

2. Psychological healthcare:

- Is data on experiences with violence / trauma systematically collected?
- Is an interpreter consulted during this procedure? If not, why not?
- What is the course of action if a current traumatisation is found?
- What should be improved according to you?

Zepro NB, *et al. BMJ Open* 2024; 14:e080654. doi: 10.1136/bmjopen-2023-080654

3. Gynaecological healthcare:

- How can an asylum-seeking woman get information on contraception and family planning that she can understand (considering language, literacy) ? How does she get information on consultations?
- Who can a woman go to if she has acute or chronic gynaecological problems (e.g. vaginal infections, bladder infections, menstrual problems, strong or foul-smelling discharge)? How well are women informed about these possibilities? Are they accessing these services, and who supports them in this? Options for improvement?
- Where are prophylactic gynaecological examinations conducted?
- Is an interpreter consulted during the examination? Who is present during the examination?

4. Healthcare during pregnancy:

- How are the pregnancy check-ups organised?
- Is the woman familiarised with different models of healthcare during pregnancy? (e.g. freelance midwife or gynaecologist, hospital care)? Are pregnant women given a choice? How can they get support (e.g. in terms of accompanying, interpreting) if they want to access ANC services?
- Which options (e.g. internet access, information material, prenatal classes) do women have to inform themselves or exchange information about pregnancy in a language they are familiar with?
- Whom can a pregnant woman contact for acute problems and emergencies? If there is no formal contact person, what do women usually do in case of emergencies?
- Which options does a pregnant woman with pregnancy issues (e.g. contractions or back pains) have, so she can be alone?

5. Care during birth:

- How is the transport to the place of child birth organised (public transport, car)?
- Is the pregnant woman accompanied by someone? If yes, by whom? (Partner, friend, acquaintance, relative). In case of an emergency: availability of ambulances? Who covers the cost of an ambulance?

NIIDS
V2.0_09.02.2021

6. Protection from Violence in the camp/Asylum Centre

- Does your centre have a violence protection concept?
- How are women protected from gender-based violence and sexual assault/harassment?
- Whom can affected women turn to if they feel harassed? Are there any administrative hurdles?
- What supervision, debriefing, or counselling is available for health professionals who treat survivors of violence?

Thank you very much for your time!

Zepro NB, *et al. BMJ Open* 2024; 14:e080654. doi: 10.1136/bmjopen-2023-080654
