# Supplementary material for: Navigating challenges: a socioecological analysis of sexual and reproductive health barriers among Eritrean refugee women in Ethiopia, using a key informant approach
Source: BMJ Open. 2024 Apr 23;14(4):e080654. doi: 10.1136/bmjopen-2023-080654 (PMC11043775; doi:10.1136/bmjopen-2023-080654)
Supplement: Supplementary data [file bmjopen-2023-080654supp001.pdf]

Additional file 3

Standards for Reporting Qualitative Research (SRQR)*
http://www.equator-network.org/reporting-guidelines/srqr/

**Title and abstract**	**Page/line no**
**Title** - Concise description of the nature and topic of the study Identifying the study as qualitative or indicating the approach (e.g., ethnography, grounded theory) or data collection methods (e.g., interview, focus group) is recommended	Page# 1, line #1-2
**Abstract** - Summary of key elements of the study using the abstract format of the intended publication; typically includes background, purpose, methods, results, and conclusions	Page# 2, line #32-53

Introduction

**Problem formulation** - Description and significance of the problem/phenomenon studied; review of relevant theory and empirical work; problem statement	Page# 3-5, line #71-151
**Purpose or research questio**n - Purpose of the study and specific objectives or questions	Page# 4-5, line #151-172

Methods

**Qualitative approach and research paradigm** - Qualitative approach (e.g., ethnography, grounded theory, case study, phenomenology, narrative research) and guiding theory if appropriate; identifying the research paradigm (e.g., postpositivist, constructivist/ interpretivist) is also recommended; rationale**	Page# 5, line #196-199 And Page# 7-8, line #251-299
**Researcher characteristics and reflexivity** - Researchers' characteristics that may influence the research, including personal attributes, qualifications/experience, relationship with participants, assumptions, and/or presuppositions; potential or actual interaction between researchers' characteristics and the research questions, approach, methods, results, and/or transferability	Page# 5, line #221-237 And #299-325
**Context** - Setting/site and salient contextual factors; rationale**	Page# 5, line #175-194
**Sampling strategy** - How and why research participants, documents, or events were selected; criteria for deciding when no further sampling was necessary (e.g., sampling saturation); rationale**	Page# 6, line #202-207 and #214-248

**Ethical issues pertaining to human subjects** - Documentation of approval by an appropriate ethics review board and participant consent, or explanation for lack thereof; other confidentiality and data security issues	Page# 24, line #891-902
**Data collection methods** - Types of data collected; details of data collection procedures including (as appropriate) start and stop dates of data collection and analysis, iterative process, triangulation of sources/methods, and modification of procedures in response to evolving study findings; rationale**	Page# 6, line #213-248

Results/findings

**Synthesis and interpretation** - Main findings (e.g., interpretations, inferences, and themes); might include development of a theory or model, or integration with prior research or theory	Page# 9-19, line #328-721
**Links to empirical data** - Evidence (e.g., quotes, field notes, text excerpts, photographs) to substantiate analytic findings	

Discussion

**Integration with prior work, implications, transferability, and contribution(s) to the field -** Short summary of main findings; explanation of how findings and conclusions connect to, support, elaborate on, or challenge conclusions of earlier scholarship; discussion of scope of application/generalizability; identification of unique contribution(s) to scholarship in a discipline or field	Page# 20-, line #722-805
**Limitations** - Trustworthiness and limitations of findings	Page# 22, line#808-821

Other

**Conflicts of interest** - Potential sources of influence or perceived influence on study conduct and conclusions; how these were managed	Page# 23, line #886
**Funding** - Sources of funding and other support; role of funders in data collection, interpretation, and reporting	Page# 23, line #878-881
