# Supplementary material for: Navigating challenges: a socioecological analysis of sexual and reproductive health barriers among Eritrean refugee women in Ethiopia, using a key informant approach
Source: BMJ Open. 2024 Apr 23;14(4):e080654. doi: 10.1136/bmjopen-2023-080654 (PMC11043775; doi:10.1136/bmjopen-2023-080654)
Supplement: Author's manuscript [file bmjopen-2023-080654.draft_revisions.pdf]

BMJ Open is committed to open peer review. As part of this commitment we make the peer review history of every article we publish publicly available.

When an article is published we post the peer reviewers' comments and the authors' responses online. We also post the versions of the paper that were used during peer review. These are the versions that the peer review comments apply to.

The versions of the paper that follow are the versions that were submitted during the peer review process. They are not the versions of record or the final published versions. They should not be cited or distributed as the published version of this manuscript.

BMJ Open is an open access journal and the full, final, typeset and author-corrected version of record of the manuscript is available on our site with no access controls, subscription charges or pay-per-view fees (http://bmjopen.bmj.com).

If you have any questions on BMJ Open's open peer review process please email info.bmjopen@bmj.com

BMJ Open

Sexual and Reproductive Health needs of Eritrean refugee women in Ethiopia: Perspectives from Health Care Providers, using a socio-ecological framework

Journal:	*BMJ Open*
Manuscript ID	bmjopen-2023-080654
Article Type:	Original research
Date Submitted by the Author:	06-Oct-2023

Complete List of Authors:	Zepro, Nejimu ; Swiss Tropical and Public Health Institute, Department of Epidemiology and Public Health; University of Basel<br>Medhanyie, Araya; Mekelle University<br>Probst-Hensch, Nicole ; Swiss Tropical and Public Health Institute, Department of Epidemiology and Public Health; University of Basel<br>Chernet, Afona; Swiss Tropical and Public Health Institute, Department of Epidemiology and Public Health; University of Basel<br>Tschopp, Rea; Swiss Tropical and Public Health Institute, Department of Epidemiology and Public Health; University of Basel<br>Abongomera, Charles; Swiss Tropical and Public Health Institute, Department of Epidemiology and Public Health; University of Basel<br>Paris, Daniel; Swiss Tropical and Public Health Institute, Department of Epidemiology and Public Health; University of Basel<br>Merten, Sonja; Swiss Tropical and Public Health Institute, Department of Epidemiology and Public Health; University of Basel

Keywords:	PUBLIC HEALTH, Sexual and gender disorders < PSYCHIATRY, REPRODUCTIVE MEDICINE, QUALITATIVE RESEARCH, MENTAL HEALTH

SCHOLARONE™
Manuscripts

I, the Submitting Author has the right to grant and does grant on behalf of all authors of the Work (as defined in the below author licence), an exclusive licence and/or a non-exclusive licence for contributions from authors who are: i) UK Crown employees; ii) where BMJ has agreed a CC-BY licence shall apply, and/or iii) in accordance with the terms applicable for US Federal Government officers or employees acting as part of their official duties; on a worldwide, perpetual, irrevocable, royalty-free basis to BMJ Publishing Group Ltd ("BMJ") its licensees and where the relevant Journal is co-owned by BMJ to the co-owners of the Journal, to publish the Work in this journal and any other BMJ products and to exploit all rights, as set out in our licence.

The Submitting Author accepts and understands that any supply made under these terms is made by BMJ to the Submitting Author unless you are acting as an employee on behalf of your employer or a postgraduate student of an affiliated institution which is paying any applicable article publishing charge ("APC") for Open Access articles. Where the Submitting Author wishes to make the Work available on an Open Access basis (and intends to pay the relevant APC), the terms of reuse of such Open Access shall be governed by a Creative Commons licence – details of these licences and which Creative Commons licence will apply to this Work are set out in our licence referred to above.

Other than as permitted in any relevant BMJ Author's Self Archiving Policies, I confirm this Work has not been accepted for publication elsewhere, is not being considered for publication elsewhere and does not duplicate material already published. I confirm all authors consent to publication of this Work and authorise the granting of this licence.

For peer review only

Sexual and Reproductive Health needs of Eritrean refugee women in Ethiopia: Perspectives from Health Care Providers, using a socio-ecological framework

*Nejimu Biza Zepro [1, 2, 3] nejimu.zepro@swisstph.ch

Araya Abrha Medhanyie [4] araya.medhanyie@gmail.com

Nicole Probst-Hensch [1, 2] nicole.probst@swisstph.ch

Afona Chernet[1, 2] afona.chernet@swisstph.ch

Rea Tschopp[1, 2, 5] rea.tschopp@swisstph.ch

Charles Abongomera[1, 2] charles.abongomera@swisstph.ch

Daniel Henry Paris [1, 2] daniel.paris@swisstph.ch

Sonja Merten[1, 2, 6] sonja.merten@swisstph.ch

[1] Department of Epidemiology and Public Health, Swiss Tropical and Public Health Institute, Allschwil, Switzerland

[2] University of Basel, Basel, Switzerland

[3] College of Health Sciences, Samara University, Afar, Ethiopia

[4] School of Public Health, College of Health Sciences, Mekelle University, Tigray, Ethiopia

[5] Armauer Hansen Research Institute, Addis Ababa, Ethiopia

[6] Center for African Studies, University of Basel, Basel, Switzerland

*Corresponding author: nejimu.zepro@swisstph.ch

For peer review only - http://bmjopen.bmj.com/site/about/guidelines.xhtml

Abstract

Background: Refugee women have worse health outcomes compared to the host population. This study aimed to find out how Health Care Providers (HCPs) in Asayta refugee camp, in Afar region, North East of Ethiopia, experience and perceive the Sexual Reproductive Health (SRH) needs of Eritrean refugee women.

Method: We used a qualitative, exploratory design with the Key Informant Research (KIR) approach. We conducted in-depth interviews with HCPs in the Asayta refugee camp and nearby district hospital. The interviews were recorded, transcribed professionally, and analyzed thematically through the lens of a socio-ecological model (SEM).

Results: Complex multi-structural factors are reported to improve SRH needs of Eritrean refugee women. High turnover and shortage of HCPs, restrictive laws, language issues, cultural inconsistencies, and gender inequalities were among the main barriers reported. The refugee women SRH needs were found to be hindered at multiple SEM levels. There is a need at the individual and interpersonal levels to overcome sociocultural barriers, and improve communication. Collaboration between the regional health system and the Refugees and Returnees Service (RRS) work structure needs to be enhanced to address the unique SRH needs of refugee women through an integrated, cross-system approach. Local humanitarian policies are expected to focus on community integration, resilience, livelihoods, access to health services, and health care.

Conclusion: SRH service needs of Eritrean refugees in Ethiopia remain severely limited due to multiple complex challenges. These challenges cut across the socio-ecological realm, from the individual to international policy implications. The overlap in findings underscores the importance of developing multilateral interventions across SEM. A bilateral collaboration between RRS service structures and Asayta district healthcare system is critical. The need for developing culturally appropriate and effective interventions is reported. Multiple and multidimensional interventions are needed. Therefore, effective refugee policies that equally address the needs of host communities are needed more than ever.

Keywords: sexual and reproductive health, humanitarian crises, refugees, Eritrea, Ethiopia, health care providers, key informant research, socio-ecological framework

For peer review only

SUMMARY BOX

What is already known?

- Limited Access to Services: Refugee women often have limited access to basic SRH services due to inadequate facilities, scarce resources, and interrupted health systems
- High Risk of gender based violence (GBV): Women and girls are particularly vulnerable to sexual violence, which leads to an increased risk of sexually transmitted infections (STIs) including HIV/AIDS, unwanted pregnancies, and unsafe abortions
- STIs and HIV: The cramped risky living conditions in every humanitarian setting can contribute to the spread of STIs and HIV.
- Maternal and Child Health: Pregnant women in refugee settings often lack adequate antenatal care and skilled birth attendants, which increases the risk of maternal and neonatal complications

What are the new findings?

- This is the first qualitative study to update the existing evidence and provide an overview of SRH needs of Eritrean refugee women in Ethiopia, as far as we know
- Entrenched in socio-cultural norms, taboos, and practices, the SRH needs of Eritrean refugee women are notably intricate.
- SRH needs of Eritrean refugee women are so complex and deeply intertwined with cultural beliefs.
- Socio-cultural expectations and multi-structural factors are the main barriers affecting refugee women SRH needs
- The existing health system is not well prepared to respond to emergencies and shocks such as disease outbreaks, natural disasters, or conflict.

What do the new findings imply?

- The key findings of this study call for the implementation of all-inclusive multidimensional evidence-based care approaches to improve the current situation of Eritrean refugee women SRH needs
- Optimal use of the existing health system, where out-of-camp interventions are clearly linked to local health services and embedded in daily practice to improve access to SRH services.
- Importance of coordination and collaboration between different refugee actors of Ethiopian Ministry of Health and International NGOs
- Key findings highlight the importance of necessitating culturally sensitive approaches SRH service provision.

For peer review only - http://bmjopen.bmj.com/site/about/guidelines.xhtml

Introduction

Migration health is a growing global public health issue that should be addressed in line with the United Nations (UN) 2030 Sustainable Development Goals (SDGs), particularly Goal 10, reducing inequalities, goal 5, gender equity and Goal 3, good health and well-being for all [1]. However, the public health priorities during migration on sexual and reproductive health (SRH) needs are often overlooked by the health system, with staggering consequences [2]. The World Health Organization (WHO) advocates for universal health coverage, leaving no one behind. This includes also the most vulnerable populations including refugees and their right to health [1]. WHO has also recognized SRH care access as a global health priority in every setting [3].

SRH needs of refugee women are often prone to risky life-threatening complications, traumatic events to serious mental illness due to poor access to SRH services, unmet need to contraceptives, unplanned pregnancy, unsafe abortion, sexual gender-based violence (SGBV), and prone to sexually transmitted infections (STI) [4]. The United Nations Higher Commission for Refugees (UNHCR) estimates that forced migration has increased from 42.75 million in 2012 to 101.1 million in 2022 as a result of persecution, hostilities, conflict, violence, abuses of human rights, and significant disruptions to the public order [5].

Ethiopia is the second most populous country in Africa (over 110 million people) and the third-largest refugee-hosting African country, sheltering 926,000 refugees and asylum seekers as of 13 June 2023, mainly from South Sudan, Somalia and Eritrea [6-8]. In addition, a total of 2.75 million internally displaced persons (IDPs), excluding IDPs from Tigray region, have been identified [9]. This creates a major burden on an already overburdened country [10].

Over 140,000 Eritreans who fled hardship and persecution in their home country are hosted in Ethiopia [11]. Some 96,223 Eritrean refugees lived in four refugee camps in the northern part of Ethiopia, Tigray region [12]. However, the two year civil war that erupted in the region in November, 2020, affected the area including these camps, and triggered a massive exodus of local civilians, and Eritrean refugees to Sudan, and internally to a nearby Afar region, which created a huge humanitarian crisis. The Afar region hosts nearly 300,000 IDPs due to the war in Tigray. IDPs have settled in the collective sites with host communities. More than 4,000 IDPs live in Semera, and another 10,000 live in Afdera town [13].

Migration negatively impacts everyone, particularly women, girls, and children are the most vulnerable groups [14]. Nearly half (48.4% and 46%) of the world's migrant population are women and children, respectively [5, 15]. Refugee women face a double burden: In addition to the hardships of forced migration, they suffer from challenges related to their gender. They are at increased risk for sexual abuse, rape, unwanted pregnancies, unsafe abortions, and the spread of STIs including HIV, short birth interval, leading to long-term mental health problems and high infant and maternal mortality [16, 17]. During the collapse of existing social systems, women are unable to care for their children and themselves. In host countries, migrant women usually bear full responsibility for family members and child care, but at the same time do not have access to resources and the ability to make autonomous decisions about them to the same extent as their male counterparts [18-20].

For peer review only

For this study, the definition of SRH was adopted from the International Conference on Population and Development (ICPD 1994), as it describes reproductive health in the context of primary health care and includes family planning, prenatal care, safe delivery, postnatal care, abortion prevention, treatment of STIs, prevention and monitoring of violence against women, and elimination of harmful traditional practices [21]. SRH is at the heart of human existence, prosperity, and well-being of individuals and societies worldwide [22]. SRH is a core component of primary health care, a universal human right, that applies to every humankind including refugees, internally displaced people and, asylum seekers, but is often forgotten [10, 23]. To realize this universal right, all affected groups must have access to SRH services so they are free to make informed choices and decisions [1, 10].

SRH is a basic human right and an essential element in achieving health, gender equity, and social justice. Women's right to SRH was recognized as a fundamental human right at the ICPD 1994 in Cairo and goes beyond refugee camps and other humanitarian emergencies [24-26]. Refugee women continue to experience poor SRH outcomes than the general population, and refugee women are over-represented in the numbers of poor SRH outcomes [26-28].

The socio-ecological model (SEM) is a theoretical framework that suggests individual behavior and health outcomes are influenced by a combination of individual, interpersonal, social, organizational and environmental complex interrelated factors. This model emphasizes one interconnectedness of these factors and the need to consider them holistically addressing public health issues. Urie Bronfenbrenner first introduced SEM model as a conceptual framework for comprehending human growth. In the 1980s, the model was organized as a theory [29]. The modified SEM in this study seeks to comprehensively assess individual, community, organizational, and societal Levels [30]. These five levels of SEM are highly interconnected [31]. Health Care Providers (HCPs) themselves experience challenges at all five levels of the SEM framework. Women's past experience about contraceptives may present challenges to use SRH services at the individual level [32, 33]. On the interpersonal level, lack of cultural competence of health care staff and communication difficulties must be considered [32]. Organizational level challenges were women's unfamiliarity with the healthcare system and a lack of training and protocols governing the delivery of healthcare.

Although Ethiopia's healthcare system is still developing, SRH care is provided free of charge as part of the public service at the primary care unit. Services are mainly provided in the Maternal and Child Health (MCH) Unit in health centers, health posts, and district hospitals. Pregnancies with complications or at risk of complications are treated in hospitals as part of specialized health care. Mainly midwives and nurses provide their services independently in health centers.

SRH challenges are recognized from the end-user (refugee) perspective, there is a lack of research on HCPs' perceptions and experiences of interacting with refugee women seeking SRH care. The perspectives of HCPs is equally important, as they often deal with, provide care to, and support refugees. HCPs can also serve as key informant researchers [34] as they are in a better position to tell what challenges and opportunities the health system pose to the refugees. Moreover, since refugees can't tell or inform the barriers and opportunities that

For peer review only - http://bmjopen.bmj.com/site/about/guidelines.xhtml

they may have from the health system, they also play a role in helping refugees navigate the healthcare system and access the services they need [31]. However, only few researchers have looked at SRH needs of refugee women from HCP's perspectives [32, 33, 35, 36]. Thus, we want to uncover how different broad-level ecological factors that impact women's SRH. Therefore, a systematic approach that considers influences that go beyond the individual level is relevant to gain a broader understanding of health service utilization [31]. This study examined the difficulties of HCPs when caring for refugee women and examine how these difficulties affect the provision of SRH care.

Findings from the study could help Ethiopian Refugees and Returnees Service (RRS) agency to improve SRH care provision for refugees and migrants. Likewise, other major stakeholders like the United Nations (UN), United Nations High Commissioner for Refugees (UNHCR), World food program (WFP), International Medical Corps (IMC) Goal Ethiopia, , and others will benefit out of the study findings. This will also allow for exploring the possibility of updating and standardizing service and training packages for SRH service providers to improve comprehensive SRH services provision in similar humanitarian settings. Thus, understanding the magnitude of the problems and the reasons behind them is crucial for program planners to design effective preventive strategies.

Method

Study setting

The study was conducted in the Asayta refugee camp in Afar, North East part of Ethiopia, which currently hosts more than 18 000 Eritrean refugees. The district is characterized by an arid climate and an ecologically fragile ecosystem, making it difficult to provide basic health care and life-saving services. It is located at 65 km South West of Semera (administrative capital) and 649 km from Addis Ababa, the capital city of Ethiopia, and shares an international border with Djibouti. The temperature varies between 30 and 45 degrees Centigrade, the altitude ranges between 350 and 500 meters above sea level, and the rainfall is irregular. The total population of the district is estimated at 47,210 where (66% live in rural areas). They hardly use health care facilities, mainly because they do not know where to get health care and access to services is difficult [37]. There are four private clinics, three public health posts, and one public district hospital. Malaria, diarrhea, and other febrile illnesses are quite common in the district due to inadequate clean water and sewage disposal facilities, inadequate availability of health facilities, low supplies, and delays in the distribution of humanitarian relief supplies [37].

Asayta refugee camp, which is located 2 km outside Asayta town, is administered by the Ethiopian government in collaboration with UNHCR. The Refugee Health Center is located inside the camp that provides primary health care to refugees. Severe illnesses that require advanced diagnostics and better treatment are referred to the Asayta District Hospital.

Study design

An exploratory qualitative study design with Key Informant Research (KIR) approach was applied [38]. Semi-structured individual interviews were conducted to explore HCPs experiences and perceptions in providing SRH care for Eritrean refugee women.

For peer review only - http://bmjopen.bmj.com/site/about/guidelines.xhtml

Study Participants

Respondents were HCPs responsible for providing SRH care to Eritrean refugee women during pregnancy, childbirth, and/or the postpartum period. HCPs are selected because they are particularly valuable as Key Informants (KIIs) in research with underserved or vulnerable populations for whom limited data or resources may be available. By sharing their knowledge and experience, HCPs can help ensure that research studies are relevant, culturally appropriate and feasible in the context of refugee women being studied.

Patient and public involvement

Neither HCPs nor KII were involved in the design, conduct or analysis of this paper. However, we are planning to hold a dissemination workshop on project key findings and consider next steps for potential impactful interventional work. Which will bring to design long-term partnership with Asayta refugee camp.

Data collection tool and Participants recruitment

We developed a separate semi-structured initial qualitative interview guide based on a review of the relevant literature that aimed at exploring HCPs' perceptions and experiences in meeting the SRH needs of women in Asayta. Study participants were requested to mention their professional background, experience, health facility responsibilities, and the proportion or number of refugee women they serve each month. Participants were then asked a series of open-ended questions about their experience in providing SRH care for Eritrean refugees.

Data collection was conducted through the KII, and focused observations. The initial interview guide was developed to comprehensively capture a core set of questions for all participants. Further questions were posed depending on the type of occupation and organization in which the interviewees worked (i.e., health center or hospital). The KII guide included general questions about the SRH needs of women as perceived by HCP's during the delivery of SRH services; the accessibility of SRH services; the challenges women and HCPs face during treatment, and recommendations on how to mitigate these challenges (see supporting materials in the Appendix). To increase the credibility and richness of the data, respondents were intentionally selected to be as diverse as possible in terms of work experience, gender, work location, and cultural background. Field notes were taken to supplement the audio recordings and document nonverbal cues. At the end of all sessions, the lead author debriefed the participants, summarizing basic information.

The first author conducted the KIIs with the support of a trained research assistant who had no prior contacts or relationships with key informants. Before the interviews began, a researcher explained the detailed objectives of the study to the participants and obtained their written and verbal consent to conduct the interview. Study participants signed a written informed consent form, read the confidentiality statement, and were reminded of their voluntary participation and their right to terminate the interview at any time. The data collection period was between July and August 2022.

Interviews were conducted face-to-face in Amharic language (Ethiopia's official language). Interviews were recorded and transcribed verbatim. Data saturation was reached when no more new information continues to emerge. A summary report of the interview was also written. After the transcriptions were finished, the audio files were removed from each

For peer review only - http://bmjopen.bmj.com/site/about/guidelines.xhtml

transcriber's personal computer. The interviews were professionally transcribed by the first author with the assistance of an experienced female qualitative researcher. The transcription were translated into English for further analysis. On the study materials or transcripts, there were no names or other identifiable personal information. COVID-19 protection measures were taken during interviews, including hand sanitizer, use of face masks, social distancing, and conducting interviews in ventilated large rooms. To maintain anonymity and confidentiality, all study participants were identified with a code.

Data Analysis

The audio recordings were listened several times to confirm the completeness of the verbatim transcription, identify contextual meanings, and make preliminary interpretations of the data. This yielded sufficient textual data to perform iterative categorization of the data for better analysis. The coding process helped us to identify common themes. Codes with similar meanings were combined and then sorted to form subcategories. The subcategories were then compared, and categories with similar concepts were combined into one category.

Due to the complexity of SRH topics, especially in such groups of women, we conducted a systematic two-stage analysis. First, we conducted a thematic analysis of the interviews. Second, taking into account the broader nature of the research question, we developed an adapted SEM model to provide a clear framework and recommendations for improving the SRH needs of Eritrean refugee women. The SEM model has been used elsewhere to understand SRH barriers for refugees. For example, Shtarkshall *et al,* and Mengesha *et al*, applied this model to examine refugee and migrant healthcare access issues in Israel and Australia [35, 39]. Mengesha and colleagues [35], Keygnaert and colleagues [40], and Wells and colleagues [41] have used the SEM framework as well. This model proved to be particularly relevant because it focuses on the synergistic relationship between individuals and their social environment [29, 35]. The model also describes the multiple interactions among individual (micro), interpersonal (meso), institutional (exo), and societal (macro) factors that influence health seeking behaviors[35].

Computer-assisted qualitative and mixed-methods data analysis software (MAXQDA 2018) was used to organize codes and themes to support analysis through close reading of transcripts and notes. Common themes were discussed further by the authors for relevance and consistency with the SEM framework. The use of SEM is justified by the fact that refugee women's use of SRH care is influenced by multiple dynamic factors that consider the interactions between different levels of SEM [42, 43]. Again, it is the interactions of refugee women with their environment, at individual, interpersonal, community, societal, institutional, and policy levels, that can influence access and utilization of SRH care by refugee women, as well as SRH care provision by HCPs in Ethiopia [42]. The data transcripts were then analyzed using the Gale *et al*, framework method, drawing from Braun and Clarke's thematic analysis [44, 45]. The analysis steps suggested by Gale *et al*. were modified and applied as follows: 1) audio transcription and translation; 2) reading and rereading the translations to become familiar with the interviews; 3) line-by-line coding using MaxQDA; 4) developing a thematic framework; 5) applying the framework; 6) placing the data into an SEM framework; and 7) interpreting the data [45].

For peer review only - http://bmjopen.bmj.com/site/about/guidelines.xhtml

Inductive approach was used to identify codes and themes from the data. Analysis began with a familiarization to the developed transcripts to assign initial codes. The data coding was done by using MAXQDA, and integrity was double-checked. A summary of the coded data set was then created with specific reports to identify initial themes. This process allowed to define core concepts and identify unique and specific stories for each theme. Finally, SEM was applied to identify and interpret factors that influence SRH care among Eritrean refugee women at the individual, interpersonal, institutional, societal, and policy levels.

Data quality

We took part in a reflective process relevant to the dynamics between the researcher and the researched. Examining critically how our social and cultural backgrounds, assumptions, and positions impacted the study process [46]. Researcher reflexivity was ensured through additional field notes, focused observations, informal conversations with concerned stakeholders, and peer debriefings after each interview. Reporting data was guided by the Consolidated Criteria for Reporting Qualitative Research (COREQ-32) [47]. Rigor and trustworthiness were assessed using the principles of credibility, reliability, confirmability, and transferability. Data credibility was enhanced through prolonged engagement, peer debriefing, written reminders, and various bracketing methods for data collection. A complete and continuous data collection method was used to ensure data reliability. The data were also reviewed by an expert researcher who had no connection to the study and was an outside observer. To confirm the data, the entire research process was recorded. Also, research colleagues who were not involved in the study but were knowledgeable about qualitative data analysis were given access to the transcripts of interviews as well as the extracted codes and categories to confirm the validity of the coding procedure. Finally, to ensure the transferability of the data, results were examined from several individuals who had similar characteristics to the participants in the study but did not participate in the present research. Data triangulation was achieved through a combination of different data sources (interviews, field notes, focused observation, and SEM theory).

The research team's interdisciplinary makeup allowed for a high level of reflexivity, which pushed us to investigate and consider assumptions and biases. All the authors have ample experience with qualitative studies, and two of the authors (SM and AAM) are recognized experts in the field of SRH in fragile humanitarian settings. We looked at how closely the retrieved themes and theme clusters matched the SEM categories. Almost 72% of the codes and themes developed were in agreement with those taken from the SEM perspective and applied by other researchers [31, 35, 39]

Ethics approval

Ethical approval was obtained from the Ethics Committee of Northwestern and Central Switzerland (EKNZ; EKNZ No. 2020-02154) in Basel, Switzerland, and from the Research Ethics Committee of the Armauer-Hansen Research Institute (AHRI) in Ethiopia as part of the "HEALTH- NIIDS" project. Permission was also obtained from the Refugees and Returnees Service (RRS) in Addis Ababa, Ethiopia. The purpose, objectives, and expectations of the study were explained to the study participants. The information sheet was read to participants in the local language so that they could understand their rights. Unique identification numbers and codes were used to maintain confidentiality. KII were conducted individually and in private. All participant related individual files, participants' identities were encrypted and kept

For peer review only - http://bmjopen.bmj.com/site/about/guidelines.xhtml

confidential via password-protected computer access; to maintain the anonymity of all data. Participation in the study was completely voluntary and written informed consent was obtained from all study subjects.

Result

One physician (general practitioner), one laboratory expert, one pharmacist, about seven nurses and three midwives were present during data collection. For a total of ten key participants (6 from camp and 4 from host community) participated in the study. In addition, five midwives, four nurses, and one senior manager were successfully interviewed. The duration of the interviews ranged from 30 to 72 minutes. The results of the study are presented thematically. The various themes that emerged were classified into several categories and subcategories. The overlapping and similar categories were grouped into five main categories. Finally, we found that many codes, themes, and categories overlapped at multiple levels of the SEM framework, indicating multilayered influences of complex issues, and we decided to use the SEM to organize the main findings from the perspective of HCPs caring for Eritrean refugee women in a humanitarian setting.

Socio-demographic characteristics of the respondents

The total number of ten HCPs participated in the study chanced gender balance (5 males and 5 females) from the refugee health center and the district hospital. The age range of participants was 25 to 34 years, indicating that most of the study participants were young employees. The minimum education level was a college diploma, while more than half of the participants had a bachelor's degree. All participants except two were not from Afar region. Most of them had a total of more than three years of work experience, while the rest had been on the job for only two or three years. The experience of working in Asayta refugee camp is quite limited with a maximum of one year. We cannot list the study participants here, as they are few and easily identified.

Themes and categories according to SEM

The analysis of the main findings of the present study related to the SRH needs of Eritrean refugee women was explored by thematically mapping the problems to five SEM levels: individual, interpersonal, organizational, community, and larger policy perspectives. The intersecting rings in the model show how factors at one level influence factors at other levels (Figure 1 below and Annex 1).

For peer review only - http://bmjopen.bmj.com/site/about/guidelines.xhtml

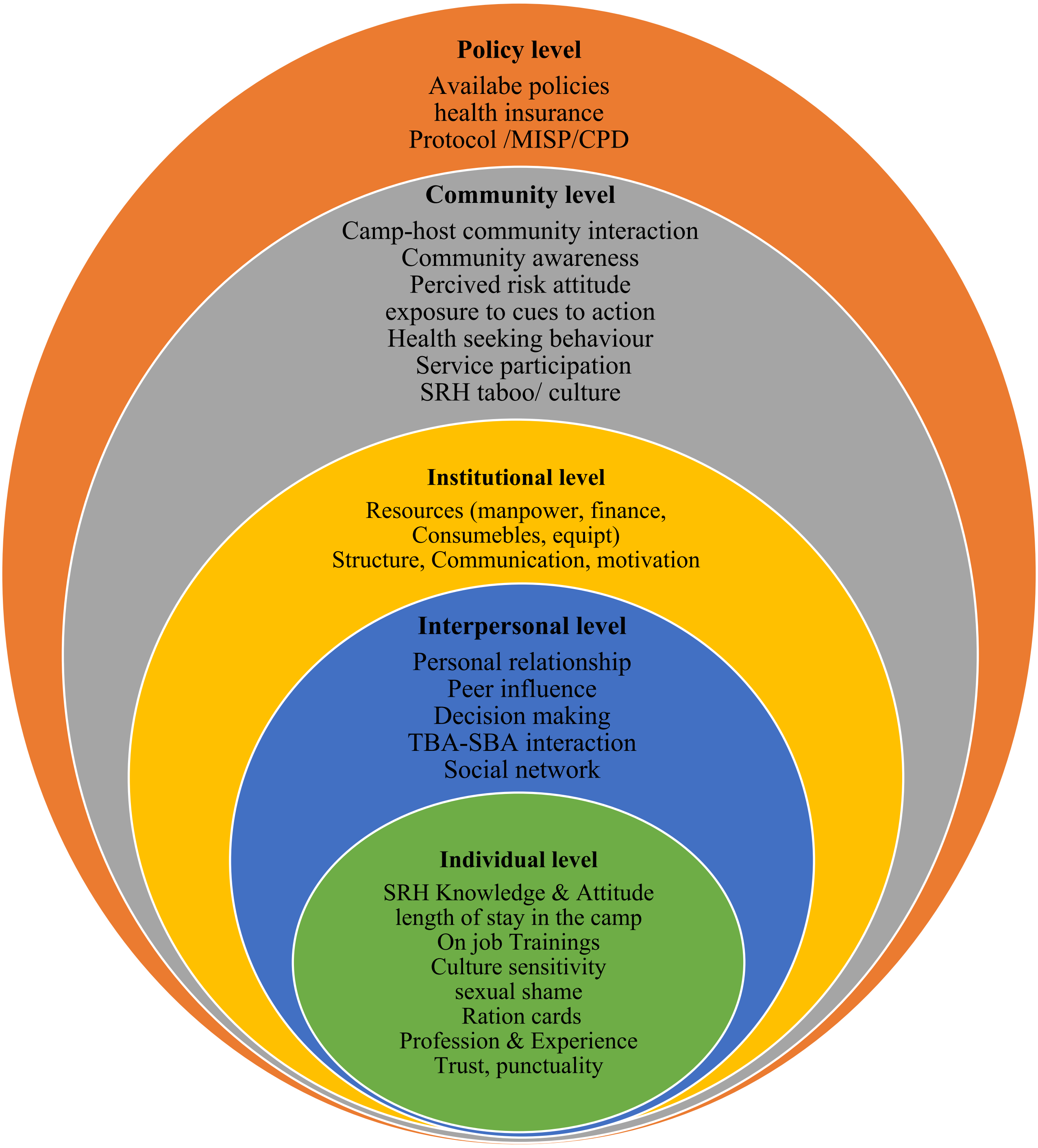

Figure 1. A socio-ecological analysis of factors (adapted) that influence HCPs' preparedness for delivering SRH services for Eritrean refugee women in Asayta refugee camp, Ethiopia.

Individual/intra personal-level factors

The first SEM level, individual level, considers personal characteristics such as age, gender, knowledge, culture, beliefs, and behavioral and lifestyle factors. Job category, camp experience, gender, ethnicity, religion, marital status, SRH knowledge, displacement experiences, and cultural taboos were identified as individual factors that impede appropriate

For peer review only - http://bmjopen.bmj.com/site/about/guidelines.xhtml

SRH service delivery. However, Eritrean refugee women were strongly influenced by their SRH knowledge, autonomy, cultural beliefs, challenges from HCPs, and stressors of adapting to a new environment.

Refugee women SRH knowledge

A lack of clear and accessible health information is mentioned as a barrier to accessing SRH care. HCPs noted that SRH services are not adequately advertised in local languages as well as refugees' mother tongue, leading to confusion among women, particularly Eritrean refugee women who have limited literacy skills, or are unfamiliar with the health system.

> *"It may be helpful to consider strategies to increase accessibility and visibility of SRH services in the refugee camp, such as using multiple communication methods (e.g., visual art, outreach), providing information in local languages, and clearly labeling the location of services."* Female nurse age 26 years.

It is explained that the refugee camp seeks to provide health education on a range of SRH topics, including STIs, contraceptive methods, prenatal care, obstetric care, postpartum care, and gender-based violence. Women must have access to accurate and relevant information about SRH issues so they can make informed decisions about their health.

However, women generally have a limited understanding of SRH issues. Women turn to multiple people for information on SRH issues, and HCPs report having to spend a great deal of time explaining and persuading women. This could be due to several factors, including cultural beliefs and practices, lack of access to education, and lack of exposure to SRH information in addition to language barriers.

> *"Eritrean refugee women's SRH knowledge is quite limited. We spend a lot of time educating refugee women about SRH issues because this information is not a high priority as there are more pressing issues for refugee women, such as housing, food, asylum applications, and social issues. They want clear and complete information about prenatal care, pregnancy, and childbirth. They also want to know how maternity issues might affect their asylum claim". Male nurse age 30 years*

Two another HCP midwifes elaborated the issue with their critical observation as fellow.

> *"when counseling Eritrean refugee women, we have to start from scratch and address multiple SRH issues, which takes more time than the expected counseling hours. There is a need for "repeated SRH counseling sessions" to build trust in SRH care."* Female midwife age 27 years

> *"Awareness among refugee women is limited. I believe that women use SRH services not because of better health behaviors, but because of the food supply and the camp's refusal to register children born outside the camp clinic".* Female nurse age 34 years

HCPs are reluctant to provide safe abortion services

Eritrean refugee women in need of abortion often turn to private clinics for safe abortion services. The HCP at the hospital said,

For peer review only - http://bmjopen.bmj.com/site/about/guidelines.xhtml

"Oh yes, we have to recognize Ethiopian abortion law.' So what's happening on the ground seems to be less official policy and more 'cultural/religious resistance' to safe abortion services." Male midwife age 30 years

Safe abortion services are generally believed to be not available due to the reluctance of HCPs. While most private clinics offer clandestine abortion service, the cost is prohibitive for refugee women and the skills of the professionals in private clinics are not reassuring. As a result, refugee women often opt for illegal, self-induced abortions, which are likely to be unsafe and put women at risk of severe bleeding. HCPs interviewed reported that refugee women with complications following unsafe abortions are admitted to public hospitals.

> *"I have never seen a refugee woman come here (to the refugee clinic) to have an abortion. They do it themselves, self-induced. They buy abortion pills from unknown sources. They even have their traditional methods, like using certain traditional medicines, and they come to us with heavy bleeding. So by the time they come to us, the pregnancy has already been terminated. So we had to refer them to Asayta Hospital, where they receive better treatment like D&C [dilation and curettage]. Curettage removes anything that is left behind."* Female midwife age 27 years

> *"It is common to see schoolgirls from the refugee camp buying abortion pills to end a pregnancy. These drugs are purchased without a doctor's prescription, just like over-the-counter medications. These pills are illegal and unregulated, and we warn young people not to buy "abortion pills" without a prescription, as this can lead to further complications."* Male nurse age 25 years

Women's autonomy

Autonomy refers to a woman's power to make decisions about her SRH services free from coercion and violence. Women's self-decision-making power enables individuals, especially marginalized or disadvantaged groups, to have more control over their own lives. For refugee women, autonomy can involve a range of activities aimed at increasing their voice and decision-making power.

> *"Refugee women who arrived at the camp reported having very limited autonomy in sexual and reproductive health issues. I think this is due to limited literacy skills, poor understanding of health issues, inability of HCPs to speak the local language, and limited social networks, usually competing with competing childcare responsibilities in large families with six to eight children in a household".* Male nurse age 32 years

Women's autonomy could be strengthened through economic empowerment. However, as a KI participant explained below, it is less likely to be pragmatic, giving the environmental and economic landscape of the region in favor of refugees.

> *"It is unlikely that the refugees will be able to find a job here. They usually go far to collect firewood in the grassland, making cultural items such as mats (called Debora) and grass rugs, which are used for Islamic prayers, raising goats for sale, or working as wage laborers in the construction sector, and factories. They usually work hard to earn little money to buy grains of wheat to feed their children".* Coordinator age 34 years

For peer review only - http://bmjopen.bmj.com/site/about/guidelines.xhtml

"The refugee women are not interested in applying for social assistance, preferring to find suitable work to meet their family's basic needs. They describe life in the refugee camp as a modern prison, even though the security situation here is much better than in Eritrea." Male nurse age 30 years

Health seeking behavior

The SRH needs of both the refugees and women in the hosting communities found to have many similarities. Among these are, have similar SRH needs related to accessing SRH services, cultural/traditional expectations and engaging in harmful traditional practices. However, refugee women were found to have additional SRH needs related to issues of referral systems, gender-based violence, women's empowerment and limited resources in refugee camps. Even though refugees and local people share common sociocultural standards and behaviors, refugee women have better health seeking behavior that differs significantly from women in the host community.

Interpersonal-level factors

The second level of the SEM perspective examines the relationships that prevent women from using SRH services. The main SRH barriers reported consistent with this SEM level were language barriers, cultural concerns, interactions with others, and peer pressure that may provide social support or create barriers that may influence a woman's perception of healthy behaviors.

Language and cultural barriers

"The first and most common challenge for all of us in here and in the community is the language problem. There is no language school or dictionary to learn the local language (Afaraf). I have tried to learn common words to help the women in their mother tongue, but I am not fluent enough to talk to the women and treat them according to their needs. All the women here want to be treated holistically, in the context of their religious, cultural, and traditional values." Male nurse age 30 years

HCPs expect their clients to be able to understand (Amharic). If this is not the case, they are expected to bring translators. Clients, on the other hand, especially newly arrived refugees, may not be aware of this problem. Instead, they expect services to be provided in their native local language (Afaraf) and that a translator will be available if HCPs do not speak the language.

"The majority of refugee women in the camp do not speak Amharic, and those with whom we cannot communicate have limited (or no) access to SRH care, even when they come and sit in front of us. That's what we find so unfortunate about the language problem in the clinic." Female midwife age 26 years

"I think that communication on both sides (client and HCP) is one of the main barriers to seeking health services. The cultural and language barrier prevents refugee women from openly expressing their needs because they are not able to clearly state their needs" Female nurse age 26 years

For peer review only - http://bmjopen.bmj.com/site/about/guidelines.xhtml

A 26-year-old female midwife at the camp, described her frustration with translators and how SRH care for adolescents' needs could be compromised. Because of only one interpreter is available at the refugee clinic. Thus, relatives or family members usually assist with translation. The involvement of relatives as interpreters in health care may raise ethical concerns: Privacy and confidentiality of the service provided.

> *"Adolescent refugees are the main users of contraceptives. This is often influenced simply by the presence of a translator who is a member of the refugee community. Adolescents are embarrassed to talk about SRH issues and contraceptives when she (translator) is present. They prefer to call my cell phone for private counseling",* Female midwife age 26 years

HCPs are accused of showing insensitivity to the cultural practices and religious beliefs of refugee women and of assuming that the way they treat them meets global scientific standards. This insensitivity is expressed in them saying:

> *"Unfortunately, we "male HCPs" continue to do what we have trained for: 'frequent touching' and 'naked exams.' However, many refugees assume they can be guaranteed a female HCP to address sensitive SRH needs, especially during childbirth."* Male nurse age 30 years

Peer pressure

Peer influence can be a powerful factor in shaping the SRH needs of refugee women. This happens when individuals feel pressured to conform to the norms, values, or behaviors of the group in order to fit in or be accepted.

> *"We experience that refugee women are often influenced by their peers. This peer pressure may result in positive or negative effects on SRH decision-making. It can also be helpful to seek support from trusted friends, family members, or health care providers when making health behavior decisions."* Male nurse age 25 years

Institutional level factors

The third SEM level on institutional factors, HCPs reported several problems impeding SRH care that are attributable to the organizational level. These bottlenecks relate to the physical work environment, duties and responsibilities, incentives, overtime pay, laboratory facilities, medications, supplies, professional development plan, organizational communication, and decision-making. Unavailability of HCPs and essential services such as central data clerk, laboratory, and pharmacy. Poor quality of services, long wait times, and prohibitive transportation costs for life-threatening emergencies were the barriers frequently reported to camp officials.

High staff turnover

HCPs cited several reasons for the high turnover in the Asayta refugee camp. These include difficult working conditions, inadequate shelter, harsh living conditions, and traumatic experiences. Other factors that may contribute to high HCPs turnover include low job satisfaction, inadequate supervision, and limited opportunities for professional development.

For peer review only - http://bmjopen.bmj.com/site/about/guidelines.xhtml

The high turnover of HCPs was mentioned as one challenge to establishing sustainable healthcare in the camp. Major collapses in care delivery were during the war in the northern Ethiopia where the camp was overwhelmed by a large number of refugees. This high turnover worsens during the pandemic. Study participant stated:

> *"It is difficult for us to convince RRS administration to accept new practices. It takes time to convince them, and on top of that, key leaders change frequently. The leader you convinced and who accepted your proposal is soon replaced, and the new leader needs another time to understand your plan, which is the biggest challenge to staying on the job. This is extremely concerning in our professional duties. To address the high turnover among HCPs in refugee camps, it may be helpful to address the root causes of this problem and implement strategies that improve working conditions, increase job satisfaction, and provide opportunities for professional development and advancement. This could include providing adequate resources and support for HCPs, improving housing and living conditions, and offering CPD opportunities."* Male nurse age 32 years

Fragmented healthcare system

HCPs employed in refugee structures function independently of the regional (district) healthcare system and are structurally responsible for the federal RRS. The refugee clinic lacks essential laboratory equipment and supplies, which can prevent refugee women from accessing SRH services. As a result, the clinic often refers its patients to other hospitals, including Asayta Hospital, Dubti Hospital, and other specialized hospital in Addis Ababa. This referral process is lengthy and cumbersome and can be challenging for patients seeking treatment, especially if they have limited transportation or financial resources. The lack of essential supplies affects the ability of health workers to make accurate diagnoses and treat patients and can lead to delays in treatment and negative health outcomes.

> *"Most of the patients are Eritreans because they have no other options to seek treatment and rely on the referral chains in the camp, which are too long and cumbersome. The lack of basic laboratory materials affects the diagnosis and treatment of clients and can lead to delays in care and negative health outcomes."* Male nurse age 32 years

Another HCP stated the following:

> *"The referral system in place in Asayta refugee camp may not be accessible or practical for women living in remote or hard-to-reach areas, such as "way up" in the mountains and off the road. In these cases, it is difficult or impossible for ambulances to reach these women, instead, mothers in labor may have to be carried to the hospital by others, which can be physically and emotionally exhausting and increase the risk of complications for both the mother and the baby".* Male nurse age 25 years

The refugee clinic lacks essential laboratory equipment and consumables, which can impact refugee women from using SRH services. As a result, the clinic frequently refers patients to other hospitals, including Asayta hospital, Dubti hospital, and specialized hospitals in Addis

For peer review only

For peer review only - http://bmjopen.bmj.com/site/about/guidelines.xhtml

Ababa. This referral process is long and cumbersome and may present challenges for patients seeking care, particularly if they have limited transportation or financial resources. Lack of essential supplies impacted the ability of HCPs to accurately diagnose and treat clients and can lead to delays in care and negative health outcomes.

Community level factors

The fourth level of SEM focuses on the broader community and societal factors that may impact the SRH needs of Eritrean refugee women living in camps and host communities. These factors include the cultural, social, and physical norms that prevail in camps and host communities, as well as the physical and social environments in which people live and work. SEM also looks at the perceived social value of HCPs and the perceived stigma associated with seeking health services, as well as satisfaction with local, state, and national government policies. In this study, community-level influences on the SRH needs of Eritrean refugees that emerged included factors within the camp setting, such as informal networks, e.g., community leaders, and the availability of community resources and interactions with the host community. All of these factors can impact refugees' ability to access the SRH services they need.

SRH is a taboo topic

It is not uncommon for certain cultural or religious practices to influence attitudes toward SRH issues. Among groups of Eritrean refugees, these practices are seen as incompatible with cultural or religious beliefs and are considered offensive or inappropriate. This makes it difficult for refugee women to access SRH services or make informed decisions about their health and can lead to negative consequences such as social exclusion or stigmatization.

HCPs explained how SRH issues are very much taboo topics:

> *"SRH issues are taboo topics that are rarely talked about within the family circle."* Female midwife age 27 years

Another HCP added,

> *"Even the thought of SRH services, contraceptives, sex before marriage, and abortion is considered "harmful to religion and culture" because culture and religion dictates remaining virginal until marriage. Adolescents who have premarital sex are considered guilty and can be excluded from the family and society."* Male midwife age 30 years

Community perceptions of SRH services can have a significant impact on the uptake of these services. In Afar communities, there are many negative attitudes or stigmas surrounding SRH services, which can make it difficult to access these services. Women view SRH services as inappropriate or immoral, or they may have cultural or religious beliefs that conflict with accessing these services. This can lead to a lack of support for SRH services within the community and make it difficult for people to access these services even when they are available. On the other hand, in communities where SRH services are viewed positively and supported by community leaders and other influential figures, these services will likely be more widely available and more widely used. HCPs we spoke with explained that refugee women and especially young adolescents have difficulty accessing SRH services for a variety

For peer review only - http://bmjopen.bmj.com/site/about/guidelines.xhtml

of reasons, including cultural norms and community perceptions. In Afar cultures, discussions of SRH issues are taboo or stigmatized, which can make it difficult for young people to access information or services on these topics. These negative perceptions of SRH services or community views that they are inappropriate to have created barriers to accessing services. These cultural and community factors can make it difficult for adolescents to access the SRH services they need, which can negatively impact their health and well-being. A HCP pointed out,

> *"When a school girl from the camp comes to the SRH clinic, people think, 'Oh, this girl has already started having sex,' and so young people are ashamed to come and ask; they prefer to solve these problems themselves."* Female midwife age 26 years

Insensitivity to cultural practices

Ethiopian health workers in the camp were generally positive about the Eritrean refugee women they care for, emphasizing that they strive to provide the same level of care to all women in need. In the Asayta refugee camp, where women come from different cultural backgrounds, they expect these cultural practices to be respected in all circumstances, but the structure of the health system makes this extremely difficult.

The study participant mentioned:

> *"This (sensitivity to cultural practices) is especially important for HCPs, who must be sensitive to the cultural practices and beliefs of the refugee women they serve to provide respectful and appropriate care. Majority of HCPs are strangers usually have difficulties in responding to the cultural needs of Eritrean refugee women, which can lead to misunderstanding or miscommunication and make it difficult for patients to receive the care they need. In some cases, the cultural insensitivity of health professionals may also contribute to a lack of trust in the healthcare system, which can further impede access to care".* Coordinator Age 32 years

Lack of husband involvement in SRH services

The lack of involvement of husbands in SRH services is a significant barrier to the uptake of these services, particularly in areas such as Afar where SRH is seen as a women's issue or where there are cultural or social norms that discourage men from becoming involved in these issues. Men's lack of participation can negatively impact men's and women's health and well-being by limiting access to information and services that are important for preventing unintended pregnancies and STIs.

> *"There are several sociocultural barriers such as restrictive norms and stigma around adolescent and young adult sexuality, harmful gender norms, and discrimination by communities, families, partners, and providers. Men have low knowledge about SRH issues. As a result, they do not support their wives to access SRH services. In general, the culture does not support the use of some SRH services, particularly family planning services. As a result, the community does not have positive attitudes toward family planning, and men and elders do not want their wives and children to limit the number of offspring they bear".* Male nurse age 25 years

For peer review only - http://bmjopen.bmj.com/site/about/guidelines.xhtml

Gender-Based Violence (GBV)

In a humanitarian setting, violence against women is more common with limited access to support systems and basic services. Common forms of violence against women in the Asayta refugee camp include physical, sexual, and emotional abuse, as well as financial exploitation and female genital mutilation (FGM). These types of violence are perpetrated by individuals, such as intimate partners or family members, or by groups or institutions. Violence against women is normalized and occurs more frequently because of conservative cultural, social, and religious norms that condone violence against women and girls as normal, acceptable behavior. A HCP stated.

> *"As a concerned professional, I know that violence against women is prevalent in our community and is not effectively addressed due to cultural, social, and religious norms. Violence against women and girls is a normal practice here and is never condemned by the community. Moreover, violence by husbands against their wives tends to be justified. Therefore, it is more difficult to recognize and respond to, as it may be considered a normal or acceptable part of daily life. This can make it more difficult to prevent GBV and provide support and services to those who have experienced it. It is important that communities and societies actively address and work to prevent the normalization of violence against women to create a safer and more equitable environment for all. This can include education and awareness-raising efforts, but also trying to change harmful cultural norms and practices that contribute to GBV."* Male nurse age 32 years

Policy level factors

This fifth level of the SEM framework adds a macro perspective that considers the broader context. The national, local, and humanitarian policies and laws that regulate or support the SRH needs of refugee women are considered.

The "Out of Camp" policy introduced by the Ethiopian government in 2010 aims to promote the integration of Eritrean refugees into the host society. This includes access to education for refugee students at all levels, including primary, secondary, and higher education at institutions such as Samara University. The Ethiopian government has taken steps to provide free maternal and reproductive health services in all public health facilities. This is an effective way to encourage women to use these services and improve SRH. However, HCPs pointed out that it is also important to address other confounding barriers that prevent women from accessing these services, such as the cost of transportation and housing.

> *"There are still indirect costs that can place a significant burden on pastoral women, especially those living in humanitarian crises, such as refugees. It might be helpful to think about implementing additional policies or programs to address these types of barriers, such as providing transportation vouchers or subsidies for housing costs. This could help ensure that all people have equal access to the health services they need."* Male nurse age 30 years

For peer review only - http://bmjopen.bmj.com/site/about/guidelines.xhtml

Weak coordination and collaboration

Lack of coordination and collaboration between different actors of RRS and the Ethiopian Ministry of Health structure and beyond led to inefficiencies and gaps in care. Coordination between the different actors responsible for the refugees is cited as a major challenge in implementing an integrated health system, especially in emergencies.

> *"Poor coordination among stakeholders has serious consequences for the effectiveness of SRH care. It can lead to duplication, confusion, and misunderstanding, which can ultimately be detrimental to patient health and well-being. It may be helpful to establish clear protocols for better communication and collaboration. This could include regular meetings or a centralized coordination system for sharing information and resources,"* Male nurse age 30 years

Training needs of HCPs at the refugee clinic

HCPs in the Asayta refugee clinic need specialized training including psychological training to meet the changing health needs of refugee women. They recognized that the SRH needs of refugee women are "more complex" and "completely different," requiring intensive preparation in culturally sensitive skills. Despite this realization, most had not participated in any training that specifically addressed refugee women's health care.

> *"Almost all refugee women have experienced trauma and have special health needs in this context. Health professionals need to be trained to recognize these needs and address them in a sensitive and culturally appropriate way. This is a very specific topic area (intercultural competence) that is not even addressed in universities in Ethiopia."* Female nurse age 26 years

Health system resilience

Another very important issue that needs to be addressed at the macro level is the resilience of health systems in the region, including in refugee camps, which are not well prepared for natural and man-made emergencies and shocks. As a result, populations lack access to basic health services during times of war, pandemics, and natural disasters. The existing health system was easily overwhelmed during the war in northern Ethiopia, the COVID-19 pandemic, and the locust invasion, leaving the population without access to essential health services.

> *"The security situation in the region and even in Ethiopia is volatile; peaceful occasions can rapidly turn into conflicts and devastating situations, as we experienced during the war in northern Ethiopia, when large numbers of people were displaced from northern Afar and Tigray to the town of Asayta, overwhelming the existing health system. At a certain stage of the peace agreement, these IDPs are forced to return to their original place of residence. In such cases, the health system is unable to meet the demands of the community, and resilience is severely compromised. Resilience-building strategies should be implemented, including strengthening emergency preparedness and response plans, increasing investment in the health system, and developing a more robust disaster forecasting and analysis system. In addition, partnerships with international organizations and other actors may be beneficial to provide additional*

For peer review only - http://bmjopen.bmj.com/site/about/guidelines.xhtml

resources and expertise to improve the health system in the region." Coordinator age 32 years

Discussion

This study reported that Eritrean refugee women face multiple challenges in accessing appropriate SRH services. The issues identified align with five level SEM framework highlighting the importance of evidence-based solutions at the individual, social, community, institutional, and policy level factors to address the SRH needs of Eritrean refugee women. A study by Vlahov and colleagues [48] also suggested designing a similar multi-level strategy to target hard-to-reach populations.

Individual-level SEM factors such as migration history, education, SRH knowledge, age, culture, socio-economic background, experience of health care, and length of stay in the camp were found to shape women's perceptions of their health and well-being, as well as their access to and utilization of SRH services. These results are in line with studies conducted [35, 49] and Multicultural Centre for Women's Health [26], which showed poor knowledge about SRH. Interactions with people, and peer pressure, which can provide social support or create barriers might affect a woman's healthy behavior perceptions. Peers, partners, and clan members influence SRH service use behaviors. Interaction between HCPs at the same time influences health service uptake [50, 51]. Language and cultural expectations were prevalent predominant themes that emerged as common SRH barriers among refugee and host community women in Asayta. Differences in language expectations were reported to affect SRH care for both HCPs and women. Similar study findings highlight that different expectations between HCPs and women are significantly affecting service quality [32, 33, 51-53]. High staff turnover, lack of essential services, laboratory, pharmacy, long waiting times, frequent referral, and transport problems were the most common barriers reported at the health facility level by study participants. Similar findings are reported from refugee camps in Kenya [54, 55].

Even though GBV is a widespread problem affecting people around the world, and it is particularly prevalent in conflict and displacement situations such as refugee camps and temporary settlements. It is concerning to witness the normalization of GBV. During migration and human traffic, there are horrific stories of violence and yet are still taken as normal in the system. This finding is supported by a study conducted in Nigeria and many other countries where community acceptance of this abusive relationship is more entrenched and where women suffer in silence as they are often protected by family secrecy, cultural norms, fear, shame, and associated social stigma and therefore remain silent [56-60]. This is mainly due to the general religious teachings that women must submit and obey their partner as head of the family [59, 61]. SRH topics in the camp are also taboo topics similar to other studies[62]. On a similar token HCPs in Asayta generally do not perform safe abortions due to prevailing negative perceptions. Women who need abortions often turn to private health facilities. The same findings are reported in many African countries where SRH is largely a taboo topic, intentionally avoided or not addressed either by the HCP or the healthcare seeker because it is socio-culturally unacceptable or connected with strong feelings of shame [63-65]. A macro perspective model that considers the broader context focus was on national, local, and humanitarian policies and laws that regulate or support the SRH needs of Eritrean refugee

For peer review only - http://bmjopen.bmj.com/site/about/guidelines.xhtml

women in the Asayta camp. These include policies on community integration, livelihood, and access to services such as healthcare, education, housing, epidemics control, and disease management. In agreement with other studies [35, 66], HCPs in this study indicated that factors at all levels of SEM influence the SRH needs of Eritrean refugee women. HCP's perceive that Eritrean refugee women SRH needs and engagement with care varied across SEM perspectives.

The SRH needs of refugees and host communities have many similarities. The mentioned difference in health seeking difference could be due to the mandatory linkage of humanitarian aid services with the use of maternal health services in the camp clinic. This can be an effective strategy to promote SRH seeking behavior, but it is also important to ensure that these services are culturally appropriate and meet the needs of the population they are intended to serve.

Under the available laws, refugees in Ethiopia are entitled to protection from persecution and to the same rights and privileges as Ethiopian citizens, with some exceptions. These include the right to education, the right to work and engage in economic activity, and the right to freedom of movement within certain limits [67, 68]. HCPs reiterated the importance of addressing indirect cost barriers that can prevent women from accessing these services, such as transportation and housing costs. Although Eritrean refugees have a right to access health services, including SRH care, they face multiple complexities where access to services is limited by several factors, including geographic remoteness, lack of resources, and socio-cultural barriers.

Due to structural differences between the regional health system and RRS, health professionals do not have easy access to continuous professional development (CPD) strategies from the national ministry of health. Most of the HCPs in the refugee camp do not have a chance to attend these CPD courses and are not up to date on the latest disease management methods, changes in treatment guidelines, etc.

Another crucial issue is the resilience of the health system in the refugee setting, which are not well prepared for emergencies and shocks such as disease outbreaks, natural disasters, or conflict. The existing health system was easily overwhelmed by triple disasters (war in northern Ethiopia, COVID-19 pandemic, and locust invasion), leaving the population without access to basic health services. Eritrean refugees were among the most vulnerable populations affected by the grave civilian atrocities, displacement, and deteriorating humanitarian situation in northern Ethiopia [69]. Thus, effective refugee policies that are designed to protect the rights and well-being of refugees, while also taking into account the needs and concerns of host communities are needed more than ever. This also highlights the need for increased support from the international community to help Ethiopia meet the needs of refugees and improve the resilience of its health system.

Strength and limitations

Our study is not without limitations. The study focused on the experiences of HCPs caring for Eritrean refugee women in Ethiopia accessing SRH services, which might be impacted by bias which may limit the transferability of findings to other ethnic refugee groups and other

For peer review only - http://bmjopen.bmj.com/site/about/guidelines.xhtml

contexts. Suggestions and conclusions were based on a small number of HCPs involved in the study. The study of SRH, such a sensitive topic, participants' fear of the researchers' intentions, and limitations in building relationships and trust within the Eritrean refugee community in Asayta camp presented challenges in recruiting participants, thus limiting women's participation and representation. However, the study used a variety of methods to better understand how refugees in Asayta camp access SRH services. HCPs were recruited from the Asayta district; therefore, comparing results with other sites may be difficult because different policies and programs apply in different refugee camps in different regions. To promote reflexivity and mitigate these biases, triangulation of evidences through focused observation was done. Despite these limitations, this study is the first attempt to examine SRH service utilization from the perspective of HCPs. This again provides a unique and insightful starting point for understanding the factors that influence SRH service utilization among women in a humanitarian crisis.

Conclusions

Humanitarian emergency settings are often characterized by the breakdown of basic health services. This study highlights the higher unmet SRH needs of Eritrean refugee women in Ethiopia, with severely limited service availability. The findings showed the need to develop interventions that simultaneously target multiple levels of SEM highlighting the importance of evidence-based solutions at the individual, group, community, institutional, and policy levels to address the SRH needs of Eritrean refugee women. Thus the provision of services requires the involvement and coordination of several complex microsystems [23, 31]. For example, at the individual level, women's past experiences can pose challenges to SRH service use. At the interpersonal level, lack of cultural competence, communication difficulties, cultural differences, misunderstandings, and misconceptions was found to affect service delivery. The results have the potential to have an even greater impact, as lessons learned from SRH decision-making can be applied to other general health services. Implementation of lessons learned about refugee health in Ethiopia should also take into account differences in culture and the traditional needs of the target populations. Underlying all solutions and their implementation should be the empowerment of women to optimize their knowledge of SRH. The findings underscore the need for interventions that address challenges at multiple levels.

Given the COVID-19 pandemic and fragile local situation, health needs could be challenged at any time, making it prepared is crucial at all levels of the SEM to ensure health system resilience. Future public health interventions must build on stakeholder input and address all aspects of SEM to achieve the SDGs and reduce maternal mortality. This could involve advocating for increased resources and funding, establishing partnerships for collaboration, and distributing supplies.

The need for better collaboration between the RRS health system and the regional (district) health system is very paramount. Developing a comprehensive and practical program, as well as legislative and policy support for this issue, can provide the foundation for SRH care for this group of women in refugee camps. Training HCPs from the local area who are familiar with culture and language can provide the services effectively. The lessons learned through HCPs

For peer review only - http://bmjopen.bmj.com/site/about/guidelines.xhtml

are important for both policy and practice, as they provide a framework for planning, developing, and implementing interventions for governments and partners charged with improving the use of SRH services. It is also important to work with community leaders and other influential figures to promote gender equality and address harmful cultural norms and practices that contribute to violence against women.

Availability of data and materials

All data collected were qualitative in nature. The data sets used and/or analyzed in the present study are available only upon reasoned request to the corresponding author.

Funding

The "HEALTH NIIDS" (Novel Integrated Infectious Disease Surveillance) project supported this work through the generous financial support of the Stanley Thomas Johnson Foundation and the State Secretariat for Education, Research and Innovation (SERI), Switzerland. The funders have no role in the design, analysis, and dissemination of the findings.

Acknowledgments

We are very grateful to all study participants for their time and valuable contributions. Our appreciation also goes to the entire NIIDS team in Switzerland and Ethiopia.

References

1. WHO, *Report on the health of refugees and migrants in the WHO European Region: no public health without refugee and migrant health (2018)* 2018, WHO: Denmark.
2. Organization., W.H., *Adolescent pregnancy: evidence brief*. 2019, World Health Organization.
3. Organization, W.H., *Universal health coverage for sexual and reproductive health: evidence brief*. 2020, World Health Organization.
4. Kurth, E., et al., *Reproductive health care for asylum-seeking women - a challenge for health professionals.* BMC Public Health, 2010. **10**: p. 659.
5. UNHCR. *Global Trends*. 10th January 2023]; Available from: https://www.unhcr.org/globaltrends
6. UNHCR. *UNHCR Ethiopia Refugee Statistics as of June 2023* 2023 [cited 2023 17.07.2023]; Available from: https://data2.unhcr.org/en/documents/download/101981.
7. Getachew, M., et al., *Magnitude and factors associated with adherence to Iron-folic acid supplementation among pregnant women in Eritrean refugee camps, northern Ethiopia.* BMC Pregnancy Childbirth, 2018. **18**(1): p. 83.
8. UNHCR. *Sharp increase in number of Eritrean refugees and asylum-seekers in Europe, Ethiopia and Sudan*. [cited 2023 10th january]; Available from: https://www.unhcr.org/news/briefing/2014/11/5465fea1381/sharp-increase-number-eritrean-refugees-asylum-seekers-europe-ethiopia.html.
9. International Organization for Migration (IOM), *International Organization for Migration (IOM), Aug 05 2022. DTM Ethiopia — National Displacement Report 12 (March— April 2022). IOM, Ethiopia.* . 2022: Switzerland.
10. UNHCR, *Inter-agency Field Manual on Reproductive Health in Humanitarian Settings*. 2010.
11. VOA, *Report: Eritrean Refugees Relocated from Addis Ababa to Dangerous Area* 2022, VOA.
12. UNHCR. *Ethiopia's Tigray Refugee Crisis Explained*. 2022 [cited 2022 13.12.2022]; Available from: https://www.unrefugees.org/news/ethiopia-s-tigray-refugee-crisis-explained/.

For peer review only - http://bmjopen.bmj.com/site/about/guidelines.xhtml

13. UNHCR. *Ethiopia's Tigray Refugee Crisis Explained*. 2022 [cited 2023 30.04]; Available from: https://www.unrefugees.org/news/ethiopia-s-tigray-refugee-crisis-explained/.
14. Gürol, E.C.T.a.S., *The effects of migration on women's health and reproductive health in Turkey.* Frontiers in Women's Health, 2019. **4**.
15. United Nations, D.o.E.a.S.A., Population Division, *International Migrant Stock 2019. (United Nations database, POP/DB/MIG/Stock/Rev.2019).* 2019, IOM: Berlin.
16. Gebrecherkos, K., et al., *Unmet need for modern contraception and associated factors among reproductive age group women in Eritrean refugee camps, Tigray, north Ethiopia: a cross-sectional study.* BMC Res Notes, 2018. **11**(1): p. 851.
17. Ahmed, J.A., et al., *Epidemiology of respiratory viral infections in two long-term refugee camps in Kenya, 2007-2010.* BMC Infectious Diseases, 2012. **12**(1): p. 7.
18. Cignacco, E., et al., *Sexual and reproductive healthcare for women asylum seekers in Switzerland: a multi-method evaluation.* BMC Health Serv Res, 2018. **18**(1): p. 712.
19. IDMC, *IDMC's 2019 Global Report on Internal Displacement*, NRC, Editor. 2019.
20. Chernet, A., *Cardiovascular diseases risk factors among recently arrived Eritrean refugees in Switzerland.* BMC Res Notes, 2019. **12**.
21. Economic, U.N.D.f., S. Information, and P. Analysis, *Population and Development: Programme of Action Adopted at the International Conference on Population and Development, Cairo, 5-13 September 1994*. Vol. 1. 1995: United Nations Publications.
22. UNFPA, *Adolescent Pregnancy: a review of evidence* 2013.
23. Ahmed, R., et al., *Situation analysis for delivering integrated comprehensive sexual and reproductive health services in humanitarian crisis condition for Rohingya refugees in Cox's Bazar, Bangladesh: protocol for a mixed-method study.* BMJ Open, 2019. **9**(7): p. e028340.
24. Fincher, R.A., *International conference on population and development.* Envtl. Pol'y & L., 1994. **24**: p. 309.
25. Austin, J., et al., *Reproductive health: a right for refugees and internally displaced persons.* Reproductive health matters, 2008. **16**(31): p. 10-21.
26. Health;, M.C.f.W.s., *Act now to advance health equity for migrant and refugee women's sexual and reproductive health*. 2021.
27. Usta, J., J.A.M. Farver, and L. Zein, *Women, war, and violence: surviving the experience.* Journal of Women's Health, 2008. **17**(5): p. 793-804.
28. Barnes, D.M. and C.L. Harrison, *Refugee women's reproductive health in early resettlement.* Journal of Obstetric, Gynecologic, & Neonatal Nursing, 2004. **33**(6): p. 723-728.
29. Bronfenbrenner, U., *The ecology of human development: Experiments by nature and design*. 1979: Harvard university press.
30. Kilanowski, J.F., *Breadth of the socio-ecological model.* Journal of Agromedicine, 2017. **22**(4): p. 295-297.
31. Mengesha, Z.B., et al., *Preparedness of Health Care Professionals for Delivering Sexual and Reproductive Health Care to Refugee and Migrant Women: A Mixed Methods Study.* Int J Environ Res Public Health, 2018. **15**(1).
32. Newbold, K.B. and J. Willinsky, *Providing family planning and reproductive healthcare to Canadian immigrants: perceptions of healthcare providers.* Culture, health & sexuality, 2009. **11**(4): p. 369-382.
33. Ng, C. and K.B. Newbold, *Health care providers' perspectives on the provision of prenatal care to immigrants.* Cult Health Sex, 2011. **13**(5): p. 561-74.
34. King, R., et al., *Barriers and facilitators to accessing skilled birth attendants in Afar region, Ethiopia.* Midwifery, 2015. **31**(5): p. 540-6.
35. Mengesha, Z.B., et al., *Refugee and migrant women's engagement with sexual and reproductive health care in Australia: A socio-ecological analysis of health care professional perspectives.* PLoS One, 2017. **12**(7): p. e0181421.

For peer review only

For peer review only - http://bmjopen.bmj.com/site/about/guidelines.xhtml

36. Lazar, J.N., et al., *Providers' Perceptions of Challenges in Obstetrical Care for Somali Women.* Obstetrics and Gynecology International, 2013. **2013**: p. 149640.

37. Fentaw, R., A. Bogale, and D. Abebaw, *Prevalence of child malnutrition in agro-pastoral households in Afar Regional State of Ethiopia.* Nutr Res Pract, 2013. **7**(2): p. 122-31.

38. Dykes, F. and R. Flacking, *Ethnographic research in maternal and child health*. 2016: Routledge Milton Park, Abingdon, Oxon.

39. Shtarkshall, R.A., F. Baynesan, and B.S. Feldman, *A socio-ecological analysis of Ethiopian immigrants' interactions with the Israeli healthcare system and its policy and service implications.* Ethnicity & health, 2009. **14**(5): p. 459-478.

40. Keygnaert, I., N. Vettenburg, and M. Temmerman, *Hidden violence is silent rape: sexual and gender-based violence in refugees, asylum seekers and undocumented migrants in Belgium and the Netherlands.* Culture, health & sexuality, 2012. **14**(5): p. 505-520.

41. Wells, R., et al., *An ecological model of adaptation to displacement: individual, cultural and community factors affecting psychosocial adjustment among Syrian refugees in Jordan.* Global Mental Health, 2018. **5**.

42. Guruge, S. and N. Khanlou, *Intersectionalities of influence: researching the health of immigrant and refugee women.* Canadian Journal of Nursing Research Archive, 2004: p. 32-47.

43. White, K., M. Ocampo, and I.C. Scarinci, *A socio-ecological approach for examining factors related to contraceptive use among recent Latina immigrants in an emerging Latino state.* Women & Health, 2017. **57**(7): p. 872-889.

44. Braun, V. and V. Clarke, *Using thematic analysis in psychology.* Qualitative Research in Psychology, 2006. **3**(2): p. 77-101.

45. Gale, N.K., et al., *Using the framework method for the analysis of qualitative data in multi-disciplinary health research.* BMC medical research methodology, 2013. **13**(1): p. 1-8.

46. Finlay, L. and B. Gough, *Reflexivity: A practical guide for researchers in health and social sciences*. 2008: John Wiley & Sons.

47. Tong, A., P. Sainsbury, and J. Craig, *Consolidated criteria for reporting qualitative research (COREQ): a 32-item checklist for interviews and focus groups.* International journal for quality in health care, 2007. **19**(6): p. 349-357.

48. Vlahov, D., et al., *Strategies for improving influenza immunization rates among hard-to-reach populations.* Journal of Urban Health, 2007. **84**: p. 615-631.

49. Korri, R., G. Froeschl, and O. Ivanova, *A Cross-Sectional Quantitative Study on Sexual and Reproductive Health Knowledge and Access to Services of Arab and Kurdish Syrian Refugee Young Women Living in an Urban Setting in Lebanon.* Int J Environ Res Public Health, 2021. **18**(18).

50. Control, C.f.D. and Prevention, *The social-ecological model: A framework for prevention*. 2015.

51. Mengesha, Z.B., et al., *Challenges in the Provision of Sexual and Reproductive Health Care to Refugee and Migrant Women: A Q Methodological Study of Health Professional Perspectives.* Journal of Immigrant and Minority Health, 2018. **20**(2): p. 307-316.

52. Essén, B., et al., *Are some perinatal deaths in immigrant groups linked to suboptimal perinatal care services?* BJOG: An International Journal of Obstetrics and Gynaecology, 2002. **109**(6): p. 677-682.

53. Pottie, K., et al., *Language proficiency, gender and self-reported health.* Canadian Journal of Public Health, 2008. **99**(6): p. 505-510.

54. Horn, R., *Responses to intimate partner violence in Kakuma refugee camp: refugee interactions with agency systems.* Social science & medicine, 2010. **70**(1): p. 160-168.

55. Wright, L.-A. and R. Plasterer, *Beyond basic education: Exploring opportunities for higher learning in Kenyan refugee camps.* Refuge, 2010. **27**: p. 42.

56. Women, U., *The World for Women and Girls Annual Report 2019-2020.* 2020.

For peer review only

For peer review only - http://bmjopen.bmj.com/site/about/guidelines.xhtml

57. OBAGBOYE, T.G., *ENTRENCHED EPIDEMIC: STEMMING THE TIDE OF VIOLENCE AGAINST WOMEN IN NIGERIA AND AFRICA.* INTERNATIONAL JOURNAL OF COMPARATIVE LAW AND LEGAL PHILOSOPHY (IJOCLLEP), 2020. **2**(1).

58. Muluneh, M.D., et al., *Gender based violence against women in sub-Saharan Africa: a systematic review and meta-analysis of cross-sectional studies.* International journal of environmental research and public health, 2020. **17**(3): p. 903.

59. Barrett, B.J. and M.S. Pierre, *Variations in women's help seeking in response to intimate partner violence: Findings from a Canadian population-based study.* Violence against women, 2011. **17**(1): p. 47-70.

60. Organization, W.H., *Responding to intimate partner violence and sexual violence against women: WHO clinical and policy guidelines*. 2013: World Health Organization.

61. Chawhanda, C., J. Levin, and L. Ibisomi, *Factors associated with sexual and reproductive health service utilisation in high migration communities in six Southern African countries.* BMC Public Health, 2022. **22**(1): p. 876.

62. Grandey, A.A., A.S. Gabriel, and E.B. King, *Tackling taboo topics: A review of the three M s in working women's lives.* Journal of Management, 2020. **46**(1): p. 7-35.

63. Traumer, L., M.H. Jacobsen, and B.S. Laursen, *Patients' experiences of sexuality as a taboo subject in the Danish healthcare system: a qualitative interview study.* Scandinavian journal of caring sciences, 2019. **33**(1): p. 57-66.

64. Kennedy, E.C., et al., *"These issues aren't talked about at home": a qualitative study of the sexual and reproductive health information preferences of adolescents in Vanuatu.* BMC Public Health, 2014. **14**(1): p. 770.

65. Steven, V.J., et al., *"Provide care for everyone please": engaging community leaders as sexual and reproductive health advocates in North and South Kivu, Democratic Republic of the Congo.* Reproductive Health, 2019. **16**(1): p. 98.

66. Akhavan, S. and S. Karlsen, *Practitioner and client explanations for disparities in health care use between migrant and non-migrant groups in Sweden: a qualitative study.* Journal of Immigrant and Minority Health, 2013. **15**(1): p. 188-197.

67. Canton, H., *United Nations High Commissioner for Refugees—UNHCR*, in *The Europa Directory of International Organizations 2021*. 2021, Routledge. p. 215-234.

68. Seyife, A., et al., *Utilization of modern contraceptives and predictors among women in Shimelba refugee camp, Northern Ethiopia.* PloS one, 2019. **14**(3): p. e0212262.

69. UNHCR, *Deteriorating conditions putting Eritrean refugees at grave risk in Tigray*. 2022, UNHCR: Switzerland

For peer review only

For peer review only - http://bmjopen.bmj.com/site/about/guidelines.xhtml

NIIDS
V2.0_09.02.2021

KII with health professionals and Key officers

Health professionals in the refugee camp/CH: health professionals treating Eritrean/Somali migrants

**Demographic Characteristics of the study participants**
Place
Date
Interviewers

ID	Age	Type of the professional*	Experience in camp/center	Religion	Education level	Nationality

***Medical doctor, Nurse, Public health etc.**

1. Survey of the present health status:

- Is a general clinical assessment conducted for women arriving at the camp/asylum centre? If yes, please indicate which kind (physical/psychological/gynaecological)?Where is the clinical assessment usually conducted? Who is usually present during the examination? Is the clinical examination done by a man or a woman? How many persons are present during a gynecological examination?
- Is an interpreter consulted during assessment? If not, why not?
- Is a professional interpreter available?
- How are the confidentiality and privacy maintained when SRH issues are discussed

2. Psychological healthcare:

- Is data on experiences with violence / trauma systematically collected?
- Is an interpreter consulted during this procedure? If not, why not?
- What is the course of action if a current traumatisation is found?
- What should be improved according to you?

For peer review only - http://bmjopen.bmj.com/site/about/guidelines.xhtml

3. Gynaecological healthcare:

- How can an asylum-seeking woman get information on contraception and family planning that she can understand (considering language, literacy) ? How does she get information on consultations?
- Who can a woman go to if she has acute or chronic gynaecological problems (e.g. vaginal infections, bladder infections, menstrual problems, strong or foul-smelling discharge)? How well are women informed about these possibilities? Are they accessing these services, and who supports them in this? Options for improvement?
- Where are prophylactic gynaecological examinations conducted?
- Is an interpreter consulted during the examination? Who is present during the examination?

4. Healthcare during pregnancy:

- How are the pregnancy check-ups organised?
- Is the woman familiarised with different models of healthcare during pregnancy? (e.g. freelance midwife or gynaecologist, hospital care)? Are pregnant women given a choice? How can they get support (e.g. in terms of accompanying, interpreting) if they want to access ANC services?
- Which options (e.g. internet access, information material, prenatal classes) do women have to inform themselves or exchange information about pregnancy in a language they are familiar with?
- Whom can a pregnant woman contact for acute problems and emergencies? If there is no formal contact person, what do women usually do in case of emergencies?
- Which options does a pregnant woman with pregnancy issues (e.g. contractions or back pains) have, so she can be alone?

5. Care during birth:

- How is the transport to the place of child birth organised (public transport, car)?
- Is the pregnant woman accompanied by someone? If yes, by whom? (Partner, friend, acquaintance, relative). In case of an emergency: availability of ambulances? Who covers the cost of an ambulance?

For peer review only - http://bmjopen.bmj.com/site/about/guidelines.xhtml

NIIDS
V2.0_09.02.2021

6. Protection from Violence in the camp/Asylum Centre

- Does your centre have a violence protection concept?
- How are women protected from gender-based violence and sexual assault/harassment?
- Whom can affected women turn to if they feel harassed? Are there any administrative hurdles?
- What supervision, debriefing, or counselling is available for health professionals who treat survivors of violence?

Thank you very much for your time!

For peer review only
For peer review only - http://bmjopen.bmj.com/site/about/guidelines.xhtml

BMJ Open

Sexual and reproductive health barriers among Eritrean refugee women in Ethiopia: Perspectives from health care providers, using a socio-ecological framework

Journal:	*BMJ Open*
Manuscript ID	bmjopen-2023-080654.R1
Article Type:	Original research
Date Submitted by the Author:	15-Mar-2024
Complete List of Authors:	Zepro, Nejimu ; Swiss Tropical and Public Health Institute, Department of Epidemiology and Public Health; University of Basel<br>Zepro, Nejimu; Swiss Tropical and Public Health Institute, Public Health Epidemiology; Semera University,<br>Medhanyie, Araya; Mekelle University<br>Probst-Hensch, Nicole ; Swiss Tropical and Public Health Institute, Department of Epidemiology and Public Health; University of Basel<br>Chernet, Afona; Swiss Tropical and Public Health Institute, Department of Epidemiology and Public Health; University of Basel<br>Tschopp, Rea; Swiss Tropical and Public Health Institute, Department of Epidemiology and Public Health; University of Basel<br>Abongomera, Charles; Swiss Tropical and Public Health Institute, Department of Epidemiology and Public Health; University of Basel<br>Paris, Daniel; Swiss Tropical and Public Health Institute, Department of Epidemiology and Public Health; University of Basel<br>Merten, Sonja; Swiss Tropical and Public Health Institute, Department of Epidemiology and Public Health; University of Basel
**Primary Subject Heading**:	Public health
Secondary Subject Heading:	Epidemiology, Global health, Health services research, Qualitative research, Sexual health
Keywords:	PUBLIC HEALTH, QUALITATIVE RESEARCH, REPRODUCTIVE MEDICINE, Health policy < HEALTH SERVICES ADMINISTRATION & MANAGEMENT

SCHOLARONE™
Manuscripts

I, the Submitting Author has the right to grant and does grant on behalf of all authors of the Work (as defined in the below author licence), an exclusive licence and/or a non-exclusive licence for contributions from authors who are: i) UK Crown employees; ii) where BMJ has agreed a CC-BY licence shall apply, and/or iii) in accordance with the terms applicable for US Federal Government officers or employees acting as part of their official duties; on a worldwide, perpetual, irrevocable, royalty-free basis to BMJ Publishing Group Ltd ("BMJ") its licensees and where the relevant Journal is co-owned by BMJ to the co-owners of the Journal, to publish the Work in this journal and any other BMJ products and to exploit all rights, as set out in our licence.

The Submitting Author accepts and understands that any supply made under these terms is made by BMJ to the Submitting Author unless you are acting as an employee on behalf of your employer or a postgraduate student of an affiliated institution which is paying any applicable article publishing charge ("APC") for Open Access articles. Where the Submitting Author wishes to make the Work available on an Open Access basis (and intends to pay the relevant APC), the terms of reuse of such Open Access shall be governed by a Creative Commons licence – details of these licences and which Creative Commons licence will apply to this Work are set out in our licence referred to above.

Other than as permitted in any relevant BMJ Author's Self Archiving Policies, I confirm this Work has not been accepted for publication elsewhere, is not being considered for publication elsewhere and does not duplicate material already published. I confirm all authors consent to publication of this Work and authorise the granting of this licence.

For peer review only

Sexual and reproductive health barriers among Eritrean refugee women in Ethiopia: Perspectives from health care providers, using a socio-ecological framework

*Nejimu Biza Zepro [1, 2, 3] nejimu.zepro@swisstph.ch

Araya Abrha Medhanyie [4] araya.medhanyie@gmail.com

Nicole Probst-Hensch [1, 2] nicole.probst@swisstph.ch

Afona Chernet[1, 2] afona.chernet@swisstph.ch

Rea Tschopp[1, 2] rea.tschopp@swisstph.ch

Charles Abongomera[1, 2] charles.abongomera@swisstph.ch

Daniel Henry Paris [1, 2] daniel.paris@swisstph.ch

Sonja Merten[1, 2, 5] sonja.merten@swisstph.ch

[1] Department of Epidemiology and Public Health, Swiss Tropical and Public Health Institute, Allschwil, Switzerland

[2] University of Basel, Basel, Switzerland

[3] College of Health Sciences, Samara University, Afar, Ethiopia

[4] School of Public Health, College of Health Sciences, Mekelle University, Tigray, Ethiopia

[5] Center for African Studies, University of Basel, Basel, Switzerland

*Corresponding author: nejimu.zepro@swisstph.ch or nejim76@gmail.com

For peer review only

Abstract

Objectives The study aimed to explore the experiences and perceptions of healthcare providers (HCP) regarding the sexual and reproductive health (SRH) challenges of Eritrean refugee women in Ethiopia.

Design A qualitative exploratory design with the key informant researcher (KIR) approach.

Setting and participants The study was conducted in the Afar regional state, North East, Ethiopia. The study participants were HCP responsible for providing SRH care for refugee women.

Results Eritrean refugee women have worse health outcomes than the host population. The SRH needs were found to be hindered at multiple layers of Socio-Ecological Model (SEM). High turnover and shortage of HCP, restrictive laws, language issues, cultural inconsistencies, and gender inequalities were among the main barriers reported. Complex multi-structural factors are needed to improve SRH needs of Eritrean refugee women.

Conclusions A complex set of issues spanning individual needs, social norms, community resources, healthcare limitations, and structural mismatches create significant barriers to fulfilling the SRH needs of Eritrean refugee women in Ethiopia**.** Factors like limited awareness, cultural taboos, lack of safe spaces, inadequate healthcare facilities, and restrictive policies all contribute to the severe limitations on SRH services available in the refugee settings. The overlap in findings underscores the importance of developing multi-level interventions that are culturally sensitive to the needs of refuge women across all SEM levels. A bilateral collaboration between Refugees and Returnees Service (RRS) structures and the Asayta district healthcare system are critically important.

Data availability statement: All data relevant to the study are included in the article or uploaded as supplementary information.

Keywords: Eritrea, Ethiopia, health care providers, humanitarian crises, key informant research, refugees, sexual and reproductive health, socio-ecological framework

Strengths and limitations of this study

- Key informant researcher (KIR) approach is found to be appropriate to explore super sensitive SRH issues such as sexuality, multiple sex partners, and intimate partner violence.
- Qualitative study design allowed for a more detailed and nuanced contextual understanding of the SRH challenges among refugee women in such fragile hard to reach settings.
- Collaboration with an experienced social science researcher, and local institutions was instrumental in building relationships and trust between the research team.
- Comparing results with other sites may be difficult considering that the study was conducted with a small sample of HCP in a defined humanitarian settings.

For peer review only

For peer review only - http://bmjopen.bmj.com/site/about/guidelines.xhtml

Introduction

Migration health is a growing global public health issue that should be addressed in line with the United Nations (UN) 2030 Sustainable Development Goals (SDG), particularly Goal 10, reducing inequalities, goal 5, gender equity and Goal 3, good health and well-being for all [1]. However, the public health priorities during migration on sexual and reproductive health (SRH) needs are often overlooked by the health system, with staggering consequences [2]. The World Health Organization (WHO) advocates for universal health coverage, leaving no one behind. This includes the most vulnerable populations including refugees [1]. WHO has also recognized SRH care access as a global health priority in every setting [3].

Previous studies have shown that SRH service needs of refugee women are often prone to poor access to SRH key services, risky life-threatening complications, traumatic events to serious mental illness, unmet need to contraceptives, unplanned pregnancy, unsafe abortion, sexual gender-based violence (SGBV), and sexually transmitted infections (STI) [4-6]. Patriarchy and violence also impacted SRH of Rohinga refugee women in Bangladesh [5, 7]. The United Nations Higher Commission for Refugees (UNHCR) estimates that forced migration has increased from 42.8 million in 2012 to 101.1 million in 2022 as a result of persecution, hostilities, conflict, violence, abuses of human rights, and significant disruptions to the public order [8].

Ethiopia is the second most populous country in Africa (over 110 million people) and the third-largest refugee-hosting African country, sheltering 926,000 refugees and asylum seekers as of 13 June 2023, mainly from South Sudan, Somalia and Eritrea [9-11]. In addition, a total of 2.8 million internally displaced persons (IDP), excluding IDP from the Tigray region, have been identified [12]. Over 140,000 Eritreans who fled hardship and persecution in their home country are hosted in Ethiopia [13]. Some 96,223 Eritrean refugees lived in four refugee camps in the northern part of Ethiopia, Tigray region [14]. However, the two year civil war that erupted in the region in November, 2020, affected the area including these camps, and triggered a massive exodus of local civilians and Eritrean refugees to Sudan, and internally to a nearby Afar region, which created a huge humanitarian crisis. The Afar region hosts nearly 300,000 IDP due to the war in Tigray. IDP have settled in the collective sites with host communities. More than 4,000 IDP live in Semera, and another 10,000 live in Afdera town [15]. This all creates a major burden on an already overburdened country [16].

Migration negatively impacts everyone, particularly women, girls, and children are the most vulnerable groups [17]. Nearly half, 48.4% and 46%, of the world's migrant population are women and children, respectively [8, 18]. Refugee women face a double burden; in addition to the hardships of forced migration, they suffer from challenges related to their gender. They are at increased risk for sexual abuse, rape, unwanted pregnancies, unsafe abortions, and the spread of sexually transmitted infections, including HIV, and short birth interval, which lead to long-term mental health problems and high infant and maternal mortality [19-22]. During the collapse of existing social systems, women are unable to care for their children and themselves. In host countries, migrant women usually bear full responsibility for family members and child care, but at the same time do not have access to resources and the ability to make autonomous decisions about them to the same extent as their male counterparts [22-25].

For peer review only - http://bmjopen.bmj.com/site/about/guidelines.xhtml

For this study, the definition of SRH was adopted from the International Conference on Population and Development (ICPD 1994) and The United Nations Population Fund (UNFPA), as it refers to a state of complete physical, mental and social well-being in all matters relating to the reproductive system. It implies the ability to be free from unwanted pregnancy, unsafe abortion, STI including HIV, prevention of violence against women, elimination of harmful traditional practices and all forms of sexual violence, [26]. SRH is at the heart of human existence, prosperity, and well-being of individuals and societies worldwide [27]. SRH is a core component of primary health care, a universal human right, that applies to every humankind including refugees, internally displaced people and, asylum seekers, but is often forgotten [7, 16]. To realize this universal right, all affected groups must have access to SRH services so they are free to make informed choices and decisions [1, 16]. SRH is a basic human right and an essential element in achieving health, gender equity, and social justice. Women's rights to SRH were recognized as fundamental human rights at the ICPD 1994 in Cairo and goes beyond refugee camps and other humanitarian emergencies [28-30]. Refugee women continue to experience poorer SRH outcomes than the general population, and refugee women are over-represented in the numbers of poor SRH outcomes [30-32].

The socio-ecological model (SEM) is a theoretical framework that suggests individual behavior and health outcomes are influenced by a combination of individual, interpersonal, social, organizational and environmentally complex interrelated factors. This model emphasizes one interconnectedness of these factors and the need to consider them holistically by addressing public health issues. Urie Bronfenbrenner (1979) first introduced the SEM model as a conceptual framework for comprehending human growth. In the 1980s, the model was organized as a theory [33]. The modified SEM in this study seeks to comprehensively assess individual, community, organizational, and societal levels [34]. These five levels of SEM are highly interconnected [35]. Health Care Providers (HCP) themselves experience challenges at all five levels of the SEM framework. Women’s past experiences about contraceptives may present challenges to use SRH services at the individual level [36, 37]. On the interpersonal level, lack of cultural competence of health care staff and communication difficulties must be considered [36]. Organizational level challenges were women's unfamiliarity with the healthcare system, a lack of training and protocols governing the delivery of healthcare.

Although Ethiopia’s healthcare system is still developing, SRH care is provided free of charge as part of the public service at the primary care unit. Services are mainly provided in the Maternal and Child Health (MCH) Unit in health centers, health posts, and district hospitals. Pregnancies with complications or at risk of complications are treated in hospitals as part of specialized health care. Mainly midwives and nurses provide their services independently in health centers.

SRH challenges are recognized from the end-user (refugee) perspective; there is a lack of research on HCP perceptions and experiences of interacting with refugee women seeking SRH care. The perspectives of HCP are equally important, as they often deal with, provide care to, and support refugees. HCP are key informant researchers as they are in a better position to tell what challenges and opportunities the health system poses to the refugees [38]. Moreover, since refugees can't tell or inform the barriers and opportunities that they may have from the health system, they also play a role in helping refugees navigate the healthcare

For peer review only - http://bmjopen.bmj.com/site/about/guidelines.xhtml

system and access the services they need [35]. However, only few researchers have looked at SRH needs of refugee women from HCP's perspectives [36, 37, 39, 40]. Thus, we want to uncover how different broad-level ecological factors that impact women's SRH. Therefore, a systematic approach that considers influences that go beyond the individual level is relevant to gain a broader understanding of health service utilization [35]. This study examined the difficulties of HCP when caring for refugee women and examined how these difficulties affected the provision of SRH care.

Findings from the study could help RRS agency to improve SRH service provision for refugees and migrants. Likewise, other major stakeholders like the United Nations (UN), United Nations High Commissioner for Refugees (UNHCR), World Food Program (WFP), International Medical Corps (IMC) Goal Ethiopia, , and others will benefit from the study findings. This will also allow for exploring the possibility of updating and standardizing service and training packages for SRH service providers to improve comprehensive SRH services provisions in similar humanitarian settings. Thus, understanding the magnitude of the problems and the reasons behind them is crucial for program planners to design effective preventive strategies.

Methods

Study setting

The study was the qualitative arm of the Novel Integrated Infectious Disease Surveillance (NIIDS) project carried out in Ethiopia and Switzerland. The study was conducted in the Asayta refugee camp in Afar, North East part of Ethiopia, which currently hosts more than 18, 000 Eritrean refugees. The district is characterized by an arid climate and an ecologically fragile ecosystem, making it difficult to provide basic health care and life-saving services. It is located at 65 km South West of Semera (administrative capital) and 649 km from Addis Ababa, the capital city of Ethiopia, and shares an international border with Djibouti. The temperature varies between 30 and 45 degrees Centigrade, the altitude ranges between 350 and 500 meters above sea level, and the rainfall is irregular. The total population of the district is estimated at 47,210 where (66% live in rural areas). They rarely use health care facilities, mainly because they do not know where to get health care and access to services is difficult [41]. There are four private clinics, three public health posts, and one public district hospital. Malaria, diarrhea, and other febrile illnesses are quite common in the district due to inadequate clean water and sewage disposal facilities, inadequate availability of health facilities, low supplies, and delays in the distribution of humanitarian relief supplies [41].

Asayta refugee camp, which is located 2 km outside Asayta town, is administered by the RRS in collaboration with UNHCR. The refugee clinic is located inside the camp that provides primary health care to Eritrean refugees. Severe illnesses that require advanced diagnostics are referred to the nearby Asayta district hospital.

Study design

An exploratory qualitative study design with a Key Informant Research (KIR) approach was applied [42]. Semi-structured individual interviews were conducted to explore the experiences and perceptions of HCP in providing SRH care for Eritrean refugee women.

For peer review only - http://bmjopen.bmj.com/site/about/guidelines.xhtml

Study participants

Study participants were HCP responsible for providing SRH care to Eritrean refugee women in the Asyta refugee camp. HCP were purposely selected because they are particularly aware of SRH needs and service uptake among Eritrean refugee women. The perspectives of HCP are equally important in research. By sharing their knowledge and experience, HCP can help ensure that research studies are relevant, culturally appropriate and feasible in the context of the refugee women being studied.

Patient and public involvement

No patients were involved. Key informants were not involved in the design, conduct or analysis of this paper. However, we are planning to hold a dissemination workshop on the project's key findings and consider future follow up projects which will result in a long-term partnership with the Asayta refugee camp.

Data collection tool and participant recruitment

We developed a separate semi-structured initial qualitative interview guide based on a review of the relevant literature [43-46]. Study participants were requested to mention their professional background, experience, health facility responsibilities, and the proportion or number of refugee women they serve each month. Participants were then asked a series of open-ended questions about their experience in providing SRH care for Eritrean refugees.

Data was collected through Key Informant Interviews (KII), and focused observations. The initial interview guide was developed to comprehensively capture a core set of questions from all participants. Further questions were posed depending on the type of occupation and organization in which the interviewees worked. The KII guide included general questions about the SRH needs of women as perceived by HCP during the delivery of SRH services, the accessibility of SRH services, the challenges women and HCP face during treatment, and recommendations on how to mitigate these challenges (see supporting materials in the Appendix). To increase the credibility and richness of the data, respondents were intentionally selected to be as diverse as possible in terms of work experience, gender, work location, and cultural background. Field notes were taken to supplement the audio recordings and document nonverbal cues. At the end of all sessions, the lead author debriefed the participants, summarizing basic information.

The first author conducted the KII with the support of a trained research assistant who had no prior contacts or relationships with study participants. Before the interviews began, a researcher explained the detailed objectives of the study to the participants and obtained their written and verbal consent to conduct the interview. Study participants signed a written informed consent form, read the confidentiality statement, and were reminded of their voluntary participation and their right to terminate the interview at any time. The data collection period was between July and August 2022.

Interviews were conducted face-to-face in the Amharic language (Ethiopia's official language). Interviews were recorded and transcribed verbatim. Data saturation was reached when no more new information continued to emerge. A summary report of each interview was also written. After the transcriptions were finished, the audio files were removed from each transcriber's personal computer. The interviews were professionally transcribed by the first

For peer review only - http://bmjopen.bmj.com/site/about/guidelines.xhtml

author with the assistance of an experienced female qualitative researcher. The transcriptions were translated into English for further analysis. On the study materials or transcripts, there were no names or other identifiable personal information. COVID-19 protection measures were taken during interviews, including use of hand sanitizers, use of face masks, social distancing, and conducting interviews in ventilated large rooms. To maintain anonymity and confidentiality, all study participants were identified with a code.

Data analysis

The audio recordings were listened to several times to confirm the completeness of the verbatim transcription, identify contextual meanings, and make preliminary interpretations of the data. This yielded sufficient textual data to perform iterative categorization of the data for better analysis. The coding process helped us to identify common themes. Codes with similar meanings were combined and then sorted to form subcategories. The subcategories were then compared, and categories with similar concepts were combined into one category.

Due to the complexity of SRH topics, especially in such groups of women, we conducted a systematic two-stage analysis. First, we conducted a thematic analysis of the interviews. Second, taking into account the broader nature of the research question, we developed an adapted SEM model to provide a clear framework and recommendations for improving the SRH needs of Eritrean refugee women. We opted for this model because it effectively illustrates the complex interplay among various factors influencing the health and well-being of refugees[22, 47]. These factors encompass individual aspects such as behaviors, social and community elements like norms, institutional and policy components such as the healthcare system, as well as structural factors including policy and protection. The SEM model has been used elsewhere to understand SRH barriers for refugees. For example, Shtarkshall et al, and Mengesha et al, applied this model to examine refugee and migrant healthcare access issues in Israel and Australia [39, 48]. Mengesha and colleagues [39], Keygnaert and colleagues [49], Tadele and colleagues [22], and Wells and colleagues [50] have used the SEM framework as well. This model proved to be particularly relevant because it focuses on the synergistic relationship between individuals and their social environment [33, 39]. The model also describes the multiple interactions among individual (micro), interpersonal (meso), institutional (exo), and societal (macro) factors that influence health seeking behaviors [22, 39, 47].

Computer-assisted qualitative and mixed-methods data analysis software (MAXQDA 2018) was used to organize codes and themes to support analysis through close reading of transcripts and notes. Common themes were discussed further by the authors for relevance and consistency with the SEM framework. The use of SEM is justified by the fact that refugee women's use of SRH care is influenced by multiple dynamic factors that consider the interactions between different levels of SEM [22, 51, 52]. Again, it is the interactions of refugee women with their environment, at individual, interpersonal, community, societal, institutional, and policy levels, that can influence access and utilization of SRH care by refugee women, as well as SRH care provision by HCP in Ethiopia [51]. The data transcripts were then analyzed using the Gale *et al*, framework method, drawing from Braun and Clarke's thematic analysis [53, 54]. The analysis steps suggested by Gale *et al*. were modified and applied as

For peer review only - http://bmjopen.bmj.com/site/about/guidelines.xhtml

follows: 1) audio transcription and translation; 2) reading and rereading the translations to become familiar with the interviews; 3) line-by-line coding using MAXQDA; 4) developing a thematic framework; 5) applying the framework; 6) placing the data into an SEM framework; and 7) interpreting the data [54].

Inductive approach was used to identify codes and themes from the data. Analysis began with a familiarization to the developed transcripts to assign initial codes. The data coding was done by using MAXQDA, and integrity was double-checked. A summary of the coded data set was then created with specific reports to identify initial themes. This process allowed to define core concepts and identify unique and specific stories for each theme. Finally, SEM was applied to identify and interpret factors that influence SRH care among Eritrean refugee women at the individual, interpersonal, institutional, societal, and policy levels.

Data quality

We took part in a reflective process relevant to the dynamics between the researcher and the researched by examining critically how our social and cultural backgrounds, assumptions, and positions impacted the study process [55]. Researcher reflexivity was ensured through additional field notes, focused observations, informal conversations with concerned stakeholders, and peer debriefings after each interview. Data dissemination and reporting was guided by the Consolidated Criteria for Reporting Qualitative Research (COREQ) and the Standards for Reporting Qualitative Research (SRQR) guidelines [56, 57]. Rigor and trustworthiness were assessed using the principles of credibility, reliability, confirmability, and transferability. Data credibility was enhanced through prolonged engagement, peer debriefing, written reminders, and various bracketing methods for data collection. A complete and continuous data collection method was used to ensure data reliability. The data were also reviewed by an expert researcher who had no connection to the study and was an outside observer. To confirm the data, the entire research process was recorded. Also, research colleagues who were not involved in the study but were knowledgeable about qualitative data analysis were given access to the transcripts of interviews as well as the extracted codes and categories to confirm the validity of the coding procedure. Finally, to ensure the transferability of the data, results were examined from several individuals who had similar characteristics to the participants in the study but did not participate in the present research. Data triangulation was achieved through a combination of different data sources (interviews, field notes, focused observation, and SEM theory).

The research team's interdisciplinary makeup allowed for a high level of reflexivity, which pushed us to investigate and consider assumptions and biases. All the authors have ample experience with qualitative studies, and two of the authors (SM and AAM) are recognized experts in the field of SRH in fragile humanitarian settings. We looked at how closely the retrieved themes and theme clusters matched the SEM categories. Almost 72% of the codes and themes developed were in agreement with those taken from the SEM perspective and applied by other researchers [35, 39, 48]

For peer review only

For peer review only - http://bmjopen.bmj.com/site/about/guidelines.xhtml

Results

Ten KIIs took part in the study, with six from the refugee camp and four from the host hospital. Among them were five midwives, four nurses, and one senior manager. The duration of the interviews ranged from 30 to 72 minutes. The results of the study are presented thematically. The various codes that emerged were classified into several categories and subcategories. The overlapping and similar categories and sub categories were grouped into five main themes. Finally, we found that many themes overlapped at multiple levels of the SEM framework, indicating multilayered influences of complex issues.

Socio-demographic characteristics of the respondents

The total of ten HCP participated in the study with a gender balance of 5 males and 5 females. The age range of participants was between 20 to 40 years, indicating that most of the study participants were young employees. The minimum education level was a college diploma, while more than half of the participants had a bachelor's degree. All participants except two were not from Afar region. Most of them had a total of more than three years of work experience, while the rest had been on the job for only two or three years. The experience of working in Asayta refugee camp is quite limited with a maximum of one year. We cannot list the study participants here, as they are few and easily identified.

Themes and categories according to SEM

The analysis of the main findings of the present study relating to the SRH needs of Eritrean refugee women was explored by thematically mapping the problems to five SEM levels: individual, interpersonal, organizational, community, and larger policy perspectives. The intersecting rings in the model show how factors at one level influence factors at other levels (Figure 1 below).

Individual/intra personal-level factors

The first SEM level, individual level, considers personal characteristics such as age, gender, knowledge, culture, beliefs, and behavioral and lifestyle factors. Job category, camp experience, gender, ethnicity, religion, marital status, SRH knowledge, displacement experiences, and cultural taboos were identified as individual factors that impede appropriate SRH service delivery. However, Eritrean refugee women were strongly influenced by their SRH knowledge, autonomy, cultural beliefs, challenges from HCP, and stressors of adapting to a new environment.

Refugee women's SRH knowledge

A lack of clear and accessible health information is mentioned as a barrier to accessing SRH care. HCP noted that SRH services are not adequately advertised in local languages as well as refugee's mother tongue, leading to confusion among women, particularly Eritrean refugee women who have limited literacy skills, or are unfamiliar with the health system.

It may be helpful to consider strategies to increase accessibility and visibility of SRH services in the refugee camp, such as using multiple communication methods (e.g., visual art, outreach), providing information in local languages, and clearly labeling the location of services.

For peer review only - http://bmjopen.bmj.com/site/about/guidelines.xhtml

It was explained that the refugee camp seeks to provide health education on a range of SRH topics, including STI, contraceptive methods, prenatal care, obstetric care, postpartum care, and gender-based violence. Women must have access to accurate and relevant information about SRH issues so they can make informed decisions about their health.

However, women generally have a limited understanding of SRH issues. Women turn to multiple people for information on SRH issues, and HCP report having to spend a great deal of time explaining and persuading women. This could be due to several factors, including cultural beliefs and practices, lack of access to education, and lack of exposure to SRH information in addition to language barriers.

> "Eritrean refugee women's SRH knowledge is quite limited. We spend a lot of time educating refugee women about SRH issues because this information is not a high priority as there are more pressing issues for refugee women, such as housing, food, asylum applications, and social issues. They want clear and complete information about prenatal care, pregnancy, and childbirth. They also want to know how maternity issues might affect their asylum claim." KII age 20-30 years.

Another HCP elaborated her critical observation as follows:

> "Awareness among refugee women is limited. There is a need for "repeated SRH counseling sessions" to build trust in SRH care. I believe that women use SRH services not because of better health behaviors, but because of the food supply and the camp's refusal to register children born outside the camp clinic." KII age 30-40 years.

HCP are reluctant to provide safe abortion services

Eritrean refugee women in need of abortion often turn to private clinics for abortion services. The HCP at the hospital said:

> "Oh yes, we have to recognize Ethiopian abortion law.' So what's happening on the ground seems to be less official policy and more 'cultural/religious resistance' to safe abortion services". KII age 20-30 years.

Safe abortion services are generally believed to be not available due to the reluctance of HCP. While most private clinics offer clandestine abortion service, the cost is prohibitive for refugee women and the skills of the professionals in private clinics are not reassuring. As a result, refugee women often opt for illegal, self-induced abortions which are likely to be unsafe and put women at risk of severe bleeding. HCP interviewed reported that refugee women with complications following unsafe abortions are admitted to public hospitals.

> "I have never seen a refugee woman come here (to the refugee clinic) to have an abortion. They do it themselves, self-induced. They buy abortion pills from unknown sources. It is also common to see schoolgirls from the refugee camp buying abortion pills to end a pregnancy. These drugs are purchased without a doctor's prescription. They even have their traditional methods, like using certain traditional medicines, and they come to us with heavy bleeding. So by the time they come to us, the pregnancy has already been terminated. So we had to refer them to Asayta Hospital, where they

For peer review only

For peer review only - http://bmjopen.bmj.com/site/about/guidelines.xhtml

> receive better treatment like D&C [dilation and curettage]. Curettage removes anything that is left behind." KII age 20-30 years

Women's autonomy

Autonomy refers to a woman's power to make decisions about her SRH services free from coercion and violence. Women's self-decision-making power enables individuals, especially marginalized or disadvantaged groups, to have more control over their own lives. For refugee women, autonomy can involve a range of activities aimed at increasing their voice and decision-making power.

> "Refugee women who arrived at the camp reported having very limited autonomy in sexual and reproductive health issues. I think this is due to limited literacy skills, poor understanding of health issues, inability of HCP to speak the local language, and limited social networks, usually competing with competing childcare responsibilities in large families with six to eight children in a household." KII age 30-40 years

Women's autonomy could be strengthened through economic empowerment. However, as a KI participant explained below, it is less likely to be pragmatic, giving the environmental and economic landscape of the region in favor of refugees.

> "It is unlikely that the refugees will be able to find a job here. They usually go far to collect firewood in the grassland, making cultural items such as mats and grass rugs, which are used for prayers, raising goats for sale, or working as wage laborers in the construction sector, and factories. They usually work hard to earn little money to buy grains of wheat to feed their children." KII 30-40 years.

Women's empowerment

The empowerment of women is crucial for unlocking their full potential. Within refugee camps, empowerment is not only a moral imperative but also vital for the overall well-being and stability of the camps. Empowered women can more effectively nurture their families, actively engage in community development, and pave the way for a brighter future for themselves and their children. Unfortunately, Eritrean refugee women lack opportunities to access programs promoting women's empowerment.

> "The refugee women are not interested in applying for social assistance, preferring to find suitable work to meet their family's basic needs. They describe life in the refugee camp as a modern prison, even though the security situation here is much better than in Eritrea." KII age 30-40 years.

Health seeking behavior

The SRH needs of both the refugees and women in the hosting communities found to have many similarities. Among these are having similar SRH needs related to accessing SRH services, cultural/traditional expectations and engaging in harmful traditional practices. However, refugee women were found to have additional SRH needs related to issues of referral systems, gender-based violence, women's empowerment and limited resources in refugee camps. Even though refugees and local people share common sociocultural standards

For peer review only - http://bmjopen.bmj.com/site/about/guidelines.xhtml

and behaviors, refugee women have better health seeking behavior that differs significantly from women in the host community.

Interpersonal-level factors

The second level of the SEM perspective examines the relationships that prevent women from using SRH services. The main SRH barriers reported consistent with this SEM level were: language barriers, cultural concerns, interactions with others, and peer pressure that may provide social support or create barriers that may influence a woman's perception of healthy behaviors.

Language and cultural barriers

> “The first and most common challenge for all of us in here and in the community is the language problem. There is no language school or dictionary to learn the local language (Afaraf). I have tried to learn common words to help the women in their mother tongue, but I am not fluent enough to talk to the women and treat them according to their needs. All the women here want to be treated holistically, in the context of their religious, cultural, and traditional values.” KII age 30-40 years.

HCP expect their clients to be able to understand (Amharic). If this is not the case, they are expected to bring translators. Clients, on the other hand, especially newly arrived refugees, may not be aware of this problem. Instead, they expect services to be provided in their native local language (Afaraf) and that a translator will be available if HCP do not speak the language.

> “The majority of refugee women in the camp do not speak Amharic, and those with whom we cannot communicate have limited (or no) access to SRH care, even when they come and sit in front of us. The cultural and language barrier prevents refugee women from openly expressing their needs because they are not able to clearly state their needs. That's what we find so unfortunate about the language problem in the clinic.” KII age 20-30 years.

A KII at the camp, described the frustration with translators and how SRH care for adolescents' needs could be compromised because only one interpreter was available at the refugee clinic. Thus, relatives or family members usually assist with translation. The involvement of relatives as interpreters in health care may raise ethical concerns regarding privacy and confidentiality of the services provided.

> “Adolescent refugees are the main users of contraceptives. This is often influenced simply by the presence of a translator who is a member of the refugee community. Adolescents are embarrassed to talk about SRH issues and contraceptives when she (translator) is present. They prefer to call my cell phone for private counseling.” KII age 20-30 years.

HCP are accused of showing insensitivity to the cultural practices and religious beliefs of refugee women, and of assuming that the way they treat them meets global scientific standards. This insensitivity is expressed in them saying:

For peer review only - http://bmjopen.bmj.com/site/about/guidelines.xhtml

"Unfortunately, we "male HCP" continue to do what we have trained for: 'frequent touching' and 'naked exams.' However, many refugees assume they can be guaranteed a female HCP to address sensitive SRH needs, especially during childbirth." KII age 30-40 years

Peer pressure

Peer influence can be a powerful factor in shaping the SRH needs of refugee women. This happens when individuals feel pressured to conform to the norms, values, or behaviors of the group in order to fit in or be accepted.

> "We experience that refugee women are often influenced by their peers. This peer pressure may result in positive or negative effects on SRH decision-making. It can also be helpful to seek support from trusted friends, family members, or health care providers when making health behavior decisions." KII age 20-30 years.

Institutional level factors

The third SEM level on institutional factors, HCP reported several problems impeding SRH care that are attributable to the organizational level. These bottlenecks relate to the physical work environment, duties and responsibilities, incentives, overtime pay, laboratory facilities, medications, supplies, professional development plan, organizational communication, and decision-making. Unavailability of HCP and essential services such as a central data clerk, laboratory, and pharmacy, poor quality of services, long wait times, and prohibitive transportation costs for life-threatening emergencies were the barriers frequently reported to camp officials.

High staff turnover

HCP cited several reasons for the high turnover in the Asayta refugee camp. These include: difficult working conditions, inadequate shelter, harsh living conditions, and traumatic experiences. Other factors that may contribute to high HCP turnover include: low job satisfaction, inadequate supervision, and limited opportunities for professional development. The high turnover of HCP was mentioned as one challenge to establishing sustainable healthcare in the camp. Major collapses in care delivery were during the war in the northern Ethiopia where the camp was overwhelmed by a large number of refugees. This high turnover worsens during the pandemic. A study participant stated:

> "It is difficult to convince RRS administration to accept new practices. It takes time to convince them, and on top of that, key leaders change frequently. The leader you convinced and who accepted your proposal is soon replaced, and the new leader needs another time to understand your plan, which is the biggest challenge to staying on the job. This is extremely concerning in our professional duties. To address the high turnover among HCP in refugee camps, it may be helpful to address the root causes of this problem and implement strategies that improve working conditions, increase job satisfaction, and provide opportunities for professional development and advancement. This could include providing adequate resources and support for HCP, improving housing and living conditions, and offering CPD opportunities." KII age 30-40 years.

For peer review only - http://bmjopen.bmj.com/site/about/guidelines.xhtml

Fragmented healthcare system

HCP employed in refugee structures function independently of the regional (district) healthcare system and are structurally responsible for the federal RRS. The refugee clinic lacks essential laboratory equipment and supplies, which can prevent refugee women from accessing SRH services. As a result, the clinic often refers its patients to other hospitals, including Asayta Hospital, Dubti Hospital, and other specialized hospital in Addis Ababa. This referral process is lengthy and cumbersome and can be challenging for patients seeking treatment, especially if they have limited transportation or financial resources. The lack of essential supplies affects the ability of health workers to make accurate diagnoses and treat patients and can lead to delays in treatment and negative health outcomes.

> “Most of the patients are Eritreans because they have no other options to seek treatment and rely on the referral chains in the camp, which are too long and cumbersome. The lack of basic laboratory materials affects the diagnosis and treatment of clients and can lead to delays in care and negative health outcomes.” KII age 30-40 years.

Another HCP provider expressed the frustration with the complex challenges of the referral system, emphasizing the difficulties of SRH services for a women from remote locations:

> “The referral system in place in Asayta refugee camp may not be accessible or practical for women living in remote or hard-to-reach areas, such as "way up" in the mountains and off the road. In these cases, it is difficult or impossible for ambulances to reach these women, instead, mothers in labor may have to be carried to the hospital by others, which can be physically and emotionally exhausting and increase the risk of complications for both the mother and the baby.” KII age 20-30 years.

Community level factors

The fourth level of SEM focuses on the broader community and societal factors that may impact the SRH needs of Eritrean refugee women living in camps and host communities. These factors include the cultural, social, and physical norms that prevail in camps and host communities, as well as the physical and social environments in which people live and work. SEM also looks at the perceived social value of HCP and the perceived stigma associated with seeking health services, as well as satisfaction with local, state, and national government policies. In this study, community-level influences on the SRH needs of Eritrean refugees that emerged included factors within the camp setting, such as informal networks, e.g. community leaders, and the availability of community resources and interactions with the host community. All of these factors can impact refugees' ability to access the SRH services they need.

SRH is a taboo topic

It is not uncommon for certain cultural or religious practices to influence attitudes toward SRH issues. Among groups of Eritrean refugees, these practices are seen as incompatible with cultural or religious beliefs and are considered offensive or inappropriate. This makes it

For peer review only - http://bmjopen.bmj.com/site/about/guidelines.xhtml

difficult for refugee women to access SRH services or make informed decisions about their health and can lead to negative consequences such as social exclusion or stigmatization.

HCP explained how SRH issues are very much taboo topics:

> "SRH issues are taboo topics that are rarely talked about within the family circle." KII age 20-30 years.

Another HCP added:

> "Even the thought of SRH services, contraceptives, sex before marriage, and abortion is considered "harmful to religion and culture" because culture and religion dictates remaining virginal until marriage. Adolescents who have premarital sex are considered guilty and can be excluded from the family and society." KII age 30-40 years.

Community perceptions of SRH services can have a significant impact on the uptake of these services. In Afar communities, there are many negative attitudes or stigmas surrounding SRH services, which can make it difficult to access these services. Women view SRH services as inappropriate or immoral, or they may have cultural or religious beliefs that conflict with accessing these services. This can lead to a lack of support for SRH services within the community and make it difficult for people to access these services even when they are available. On the other hand, in communities where SRH services are viewed positively and supported by community leaders and other influential figures, these services will likely be more widely available and more widely used. HCP we spoke with explained that refugee women and especially young adolescents have difficulty accessing SRH services for a variety of reasons, including cultural norms and community perceptions. In Afar cultures, discussions of SRH issues are taboo or stigmatized, which can make it difficult for young people to access information or services. These negative perceptions of SRH services or that the community views that they are inappropriate, have created barriers to accessing services. These cultural and community factors can make it difficult for adolescents to access the SRH services they need, which can negatively impact their health and well-being. A HCP pointed out:

> "When a school girl from the camp comes to the SRH clinic, people think, 'Oh, this girl has already started having sex,' and so young people are ashamed to come and ask; they prefer to solve these problems themselves". KII age 20-30 years

Insensitivity to cultural practices

Ethiopian health workers in the camp were generally positive about the Eritrean refugee women they care for, emphasizing that they strive to provide the same level of care to all women in need. In the Asayta refugee camp, where women come from different cultural backgrounds, they expect these cultural practices to be respected in all circumstances, but the structure of the health system makes this extremely difficult.

> "This (sensitivity to cultural practices) is especially important for HCP, who must be sensitive to the cultural practices and beliefs of the refugee women they serve to provide respectful and appropriate care. Majority of HCP are strangers usually have difficulties in responding to the cultural needs of Eritrean refugee women, which can lead to misunderstanding or miscommunication and make it difficult for patients to

receive the care they need. In some cases, the cultural insensitivity of health professionals may also contribute to a lack of trust in the healthcare system, which can further impede access to care.” KII age 30-40 years.

Lack of husband involvement in SRH services

The lack of involvement of husbands in SRH services is a significant barrier to the uptake of these services, particularly in areas such as Afar where SRH is seen as a woman's issue or where there are cultural or social norms that discourage men from becoming involved in these issues. Men's lack of participation can negatively impact men’s and women's health and well-being by limiting access to information and services that are important for preventing unintended pregnancies and STI.

> “There are several sociocultural barriers such as restrictive norms and stigma around adolescent and young adult sexuality, harmful gender norms, and discrimination by communities, families, partners, and providers. Men have low knowledge about SRH issues. As a result, they do not support their wives to access SRH services. In general, the culture does not support the use of some SRH services, particularly family planning services. As a result, the community does not have positive attitudes toward family planning, and men and elders do not want their wives and children to limit the number of offspring they bear.” KII age 20-30 years.

For peer review only - http://bmjopen.bmj.com/site/about/guidelines.xhtml

Gender-Based Violence (GBV)

In a humanitarian setting, violence against women is more common with limited access to support systems and basic services. Common forms of violence against women in the Asayta refugee camp include physical, sexual, and emotional abuse, as well as financial exploitation and female genital mutilation (FGM). These types of violence are perpetrated by individuals, such as intimate partners or family members, or by groups or institutions. Violence against women is normalized and occurs more frequently because of conservative cultural, social, and religious norms that condone violence against women and girls as normal, acceptable behavior. A HCP stated:

> "As a concerned professional, I know that violence against women is prevalent in our community and is not effectively addressed due to cultural, social, and religious norms. Violence against women and girls is a normal practice here and is never condemned by the community. Moreover, violence by husbands against their wives tends to be justified. Therefore, it is more difficult to recognize and respond to, as it may be considered a normal or acceptable part of daily life. This can make it more difficult to prevent GBV and provide support and services to those who have experienced it. It is important that communities and societies actively address and work to prevent the normalization of violence against women to create a safer and more equitable environment for all. This can include education and awareness-raising efforts, but also trying to change harmful cultural norms and practices that contribute to GBV." KII age 30-40 years.

Policy level factors

This fifth level of the SEM framework adds a macro perspective that considers the broader context. The national, local, and humanitarian policies and laws that regulate or support the SRH needs of refugee women are considered.

The "Out of Camp" policy introduced by the Ethiopian government in 2010 aims to promote the integration of Eritrean refugees into the host society. This includes access to education for refugee students at all levels, including primary, secondary, and higher education institutions. The Ethiopian government has taken steps to provide free SRH services in all public health facilities. This is an effective way to encourage women to use these services and improve SRH. However, HCP pointed out that it is also important to address other confounding barriers that prevent women from accessing these services, such as the cost of transportation and housing.

> *"There* are still indirect costs that can place a significant burden on pastoral women, especially those living in humanitarian crises, such as refugees. It might be helpful to think about implementing additional policies or programs to address these types of barriers, such as providing transportation vouchers or subsidies for housing costs. This could help ensure that all people have equal access to the health services they need." KII age 30-40 years.

For peer review only - http://bmjopen.bmj.com/site/about/guidelines.xhtml

Weak coordination and collaboration

Lack of coordination and collaboration between different actors of RRS and the Ethiopian Ministry of Health structure and beyond led to inefficiencies and gaps in care. Coordination between the different actors responsible for the refugees is cited as a major challenge in implementing an integrated health system, especially in emergencies.

> "Poor coordination among stakeholders has serious consequences. It can lead to duplication, confusion, and misunderstanding, which can ultimately be detrimental to patient health and well-being. It may be helpful to establish clear protocols for better communication and collaboration. This could include regular meetings or a centralized coordination system for sharing information and resources." KII age 30-40 years.

Training needs of HCP at the refugee clinic

HCP in the Asayta refugee clinic need specialized training including psychological training to meet the changing health needs of refugee women. They recognized that the SRH needs of refugee women are "more complex" and "completely different," requiring intensive preparation in culturally sensitive skills. Despite this realization, most had not participated in any training that specifically addressed refugee women's health care.

> "Almost all refugee women have experienced trauma and all demand special needs. HCP need to be trained to recognize these needs and address them in a sensitive and culturally appropriate way. This is a very specific topic area (intercultural competence) that is not even addressed in universities." KII age 20-30 years.

Health system resilience

Another very important issue that needs to be addressed at the macro level is the resilience of health systems in the region, including in refugee camps, which are not well prepared for natural and man-made emergencies and shocks. As a result, populations lack access to basic health services during times of war, pandemics, and natural disasters. The existing health system was easily overwhelmed during the war in northern Ethiopia, the COVID-19 pandemic, and the locust invasion, leaving the population without access to essential health services.

> "The security situation in the region and even in Ethiopia is volatile; peaceful occasions can rapidly turn into conflicts and devastating situations, as we experienced during the war in northern Ethiopia, when large numbers of people were displaced from northern Afar and Tigray to the town of Asayta, overwhelming the existing health system. At a certain stage of the peace agreement, these IDP are forced to return to their original place of residence. In such cases, the health system is unable to meet the demands of the community, and resilience is severely compromised. Resilience-building strategies should be implemented, including strengthening emergency preparedness and response plans, increasing investment in the health system, and developing a more robust disaster forecasting and analysis system. In addition, partnerships with international organizations and other actors may be beneficial to provide additional

For peer review only - http://bmjopen.bmj.com/site/about/guidelines.xhtml

resources and expertise to improve the health system in the region." KII age 30-40 years.

Discussion

Refugee women encounter significant inequalities in accessing and uptake of SRH services despite substantial influx of migrants towards developing countries. Eritrean refugee women experienced worse health outcomes than the host population. This study identified numerous barriers hindering refugee women's access to appropriate SRH services. Analyzed through a Socio-Ecological Model (SEM) framework, these challenges spanned five levels: individual, social, community, institutional, and policy. A similar multi-level intervention approach is suggested by Vlahov et al. to address the needs of women in humanitarian settings [58]. This reinforces the notion that providing effective SRH services requires collaboration and coordination across various complex systems, as highlighted by studies conducted in Bangladesh and Australia [7, 35].

Individual-level SEM factors such as migration history, education, SRH knowledge, age, culture, socio-economic background, experience of health care, and length of stay in the camp were found to shape women's perceptions of their health and well-being, as well as their access to and utilization of SRH services. These results are in line with studies conducted [39, 59] and Multicultural Centre for Women's Health [30], which showed poor knowledge about SRH. Interactions with people, and peer pressure, which can provide social support or create barriers might affect a woman's healthy behavior perceptions. Peers, partners, and clan members influence SRH service use behaviors. Interaction between HCP at the same time influences health service uptake [60, 61]. Language and cultural expectations were prevalent predominant themes that emerged as common SRH barriers among refugee and host community women in Asayta. Differences in language expectations were reported to affect SRH care for both HCP and women. Similar study findings highlight that different expectations between HCP and women are significantly affecting service quality [36, 37, 61-63]. High staff turnover, lack of essential services, laboratory, pharmacy, long waiting times, frequent referral, and transport problems were the most common barriers reported at the health facility level by study participants. Similar findings are reported from refugee camps in Kenya [64, 65].

Even though GBV is a widespread problem affecting people around the world, and it is particularly prevalent in conflict and displacement situations such as refugee camps and temporary settlements, it is concerning to witness the normalization of GBV. During migration and human trafficking, there are horrific stories of violence and yet are still taken as normal in the system. This finding is supported by a study conducted in Nigeria and many other countries where community acceptance of this abusive relationship is more entrenched and where women suffer in silence as they are often protected by family secrecy, cultural norms, fear, shame, and associated social stigma and therefore remain silent [66-70]. This is mainly due to the general religious teachings that women must submit and obey their partner as head of the family [69, 71]. SRH topics in the camp are also taboo topics similar to other studies[72]. On a similar token HCP in Asayta generally do not perform safe abortions due to prevailing negative perceptions. Women who need abortions often turn to private health facilities. The same findings are reported in many African countries where SRH is largely a

For peer review only - http://bmjopen.bmj.com/site/about/guidelines.xhtml

taboo topic, intentionally avoided or not addressed either by the HCP or the healthcare seeker because it is socio-culturally unacceptable or connected with strong feelings of shame [6, 73, 74]. A macro perspective model that considers the broader context focus was on national, local, and humanitarian policies and laws that regulate or support the SRH needs of Eritrean refugee women in the Asayta camp. These included policies on community integration, livelihood, and access to services such as healthcare, education, housing, epidemics control, and disease management. In agreement with other studies [39, 75], HCP in this study indicated that factors at all levels of SEM influence the SRH needs of Eritrean refugee women. HCP perceived that Eritrean refugee women SRH needs and engagement with care varied across SEM perspectives.

The SRH needs of refugees and host communities have many similarities. The mentioned difference in a health seeking difference could be due to the mandatory linkage of humanitarian aid services with the use of maternal health services in the camp clinic. This can be an effective strategy to promote SRH seeking behavior, but it is also important to ensure that these services are culturally appropriate and meet the needs of the population they are intended to serve.

Under the available laws, refugees in Ethiopia are entitled to protection from persecution and to the same rights and privileges as Ethiopian citizens, with some exceptions. These include the right to education, the right to work and engage in economic activity, and the right to freedom of movement within certain limits [76, 77]. HCP reiterated the importance of addressing indirect cost barriers that can prevent women from accessing these services, such as transportation and housing costs. Although Eritrean refugees have a right to access health services, including SRH care, they face multiple complexities where access to services is limited by several factors, including geographic remoteness, lack of resources, and socio-cultural barriers.

The lack of alignment between the national health system and the RRS system of refugee creates barriers for HCP in those camps and they face challenges in accessing continuous professional development (CPD) opportunities. As a result, most camp providers lack access to the latest advancements in disease management, treatment protocols, and other essential areas, potentially compromising the quality of care they can deliver.

Another crucial issue is the resilience of the health system in the refugee setting. This situation is not well prepared for emergencies and shocks such as disease outbreaks, natural disasters, or conflict. The existing health system was easily overwhelmed by triple disasters (war in northern Ethiopia, COVID-19 pandemic, and locust invasion), leaving the population without access to basic health services. Eritrean refugees were among the most vulnerable populations affected by the grave civilian atrocities, displacement, and deteriorating humanitarian situation in northern Ethiopia [78]. Thus, effective refugee policies that are designed to protect the rights and well-being of refugees, while also taking into account the needs and concerns of host communities are needed more than ever. This also highlights the need for increased support from the international community to help Ethiopia meet the needs of refugees and improve the resilience of its health system.

For peer review only - http://bmjopen.bmj.com/site/about/guidelines.xhtml

Strength and limitations

The socioecological model enabled this study to comprehensively explore the multi-level factors influencing refugee women's SRH needs, from individual experiences to societal structures, revealing insights not captured by previous studies in the country. This study is the first attempt to examine SRH service utilization from the perspective of key informant researcher approach. This again provides a unique and insightful starting point for understanding the factors that influence SRH service utilization among refugee women in a humanitarian crisis settings. Exploring SRH studies presents unique challenges due to the sensitive nature of the topic. Participants may hold biases or fears regarding the researchers' intentions, potentially introducing bias into the study. Additionally, building trust and rapport with participants can be difficult, impacting the study's success. HCP were recruited from the Asayta district; therefore, comparing results with other sites may be difficult because different policies and programs apply in different refugee camps in different regions. To promote reflexivity and mitigate these biases, triangulation of evidences through focused observation was done.

Implications for Research, Policy, and Practice

This qualitative study provides invaluable insight into the SRH of Eritrean refugee women from the perspectives of HCP. The study findings confirmed that SRH topics are taboos and act as a barrier to access SRH services. Likewise, it should be ensured that HCP working in the area of SRH should be equipped with the proper training on cultural, compassionate, and respectful competency care approaches. SRH consultations should take place in a safe and supportive manner. Healthcare facilities in the camp should also create private spaces for consultations to maintain the confidentiality of SRH services. Further research is needed to explore the knowledge and attitudes of HCP in SRH care approaches, and in understanding the barriers and facilitators towards providing comprehensive SRH service. This has the potential to facilitate the proper use of SRH services consequently leading to overall improvements to women health.

Even though SRH is included in Ethiopian social policy, the implementation of rules and regulation are not available or very minimal. It is critical to establish national policies and guidelines describing the standard of care in the area of SRH and clarify providers' roles in offering SRH advice. The findings may also be useful for public health implementers and policy evaluators, as they could broaden their understanding of what works more effectively and for whom, in fragile humanitarian contexts, with the aim of improving SRH service use among refugee women.

Conclusions

Humanitarian care settings are often characterized by the breakdown of basic health services. This study highlights the higher unmet SRH needs of Eritrean refugee women in Ethiopia, with severely limited service availability. The findings showed the need to develop interventions that simultaneously target multiple levels of SEM highlighting the importance of evidence-

For peer review only - http://bmjopen.bmj.com/site/about/guidelines.xhtml

based solutions at the individual, group, community, institutional, and policy levels to address the SRH needs of Eritrean refugee women. For example, at the individual level, women's past experiences can pose challenges to SRH service use. At the interpersonal level, lack of cultural competence, communication difficulties, cultural differences, misunderstandings, and misconceptions were found to impact service delivery. The results have the potential to have an even greater impact, as lessons learned from SRH decision-making can be applied to other general health services. Implementation of lessons learned about refugee health in Ethiopia should also take into account differences in culture and the traditional needs of the target populations. Underlying all solutions and their implementation should be the empowerment of women to optimize their knowledge of SRH.

Given the COVID-19 pandemic and fragile situation, health needs could be challenged at any time, preparation is crucial at all levels of the SEM to ensure health system resilience. Future public health interventions must build on stakeholder input and address all aspects of SEM to achieve the SDG and reduce maternal mortality. This could involve advocating for increased resources and funding, establishing partnerships for collaboration, and distributing supplies.

The need for better collaboration between the RRS health system and the regional (district) health system is very paramount. Developing a comprehensive and practical program, as well as legislative and policy support for this issue, can provide the foundation for SRH care for this group of women in refugee camps. Training HCP from the local area who are familiar with culture and language can provide the services effectively. The lessons learned through HCP are important for both policy and practice, as they provide a framework for planning, developing, and implementing interventions for governments and partners charged with improving the use of SRH services. It is also important to work with community leaders and other influential figures to promote gender equality and address harmful cultural norms.

Authors' contributions

NBZ: conceptualisation, methodology, investigation, analysis, writing original draft. SM, AAM, DHP, and NPH provided critical review and supervision. CA, AC and RT contributed for project development and coordination. SM, AAM, RT, CA, AC, DHP and NPH contributed and reviewed the article prior to submission. All authors have read and approved the final version.

Funding

The "HEALTH NIIDS"project supported this work through the generous financial support of the Stanley Thomas Johnson Foundation and the State Secretariat for Education, Research and Innovation (SERI), Switzerland with grant number 1053-KF. The funders have no role in the design, analysis, and dissemination of the findings.

Ethics statements

Patient consent for publication

Not applicable.

Competing interests

For peer review only - http://bmjopen.bmj.com/site/about/guidelines.xhtml

The authors declare that they have no competing interests

Availability of data and materials

All data collected were qualitative in nature. The data relevant to the study are included in the article or uploaded as supplementary information.

Ethics approval

Ethical approval was obtained from the Ethics Committee of Northwestern and Central Switzerland (EKNZ; EKNZ No. 2020-02154) in Basel, Switzerland, and from the Research Ethics Committee of the Armauer-Hansen Research Institute (AHRI) in Ethiopia as part of the "HEALTH- NIIDS" project. Permission was also obtained from the Refugees and Returnees Service (RRS) in Addis Ababa, Ethiopia. The purpose, objectives, and expectations of the study were explained to the study participants. The information sheet was read to participants in the local language so that they could understand their rights. Unique identification numbers and codes were used to maintain confidentiality. KII were conducted individually and in private. All participant related individual files and participants' identities were encrypted and kept confidential via password-protected computer access to maintain the anonymity of all data. Participation in the study was completely voluntary and written informed consent was obtained from all study subjects.

Acknowledgments

We are very grateful to all study participants for their time and valuable contributions. Our appreciation goes to the entire NIIDS project team in Switzerland and Ethiopia. We are also grateful to Karen Maigetter for her invaluable assistance with the language edits.

Figure 1. A socio-ecological analysis framework of factors that influence HCPs' readiness to provide SRH services to Eritrean refugee women in Asayta refugee camp, Ethiopia (adapted).

References

1. WHO, *Report on the health of refugees and migrants in the WHO European Region: no public health without refugee and migrant health (2018)* 2018, WHO: Denmark.
2. Organization., W.H., *Adolescent pregnancy: evidence brief*. 2019, World Health Organization.
3. Organization, W.H., *Universal health coverage for sexual and reproductive health: evidence brief*. 2020, World Health Organization.
4. Kurth, E., et al., *Reproductive health care for asylum-seeking women - a challenge for health professionals.* BMC Public Health, 2010. **10**: p. 659.
5. Jannat, S., R.I. Sifat, and M. Khisa, *Sexual and Reproductive Health Conditions of Women: Insights from Rohingya Refugee Women in Bangladesh.* Sexuality Research and Social Policy, 2023. **20**(3): p. 855-868.
6. Kennedy, E.C., et al., *"These issues aren't talked about at home": a qualitative study of the sexual and reproductive health information preferences of adolescents in Vanuatu.* BMC Public Health, 2014. **14**(1): p. 770.
7. Ahmed, R., et al., *Situation analysis for delivering integrated comprehensive sexual and reproductive health services in humanitarian crisis condition for Rohingya refugees in Cox's Bazar, Bangladesh: protocol for a mixed-method study.* BMJ Open, 2019. **9**(7): p. e028340.

For peer review only - http://bmjopen.bmj.com/site/about/guidelines.xhtml

8. UNHCR. *Global Trends*. 10th January 2023]; Available from: https://www.unhcr.org/globaltrends
9. UNHCR. *UNHCR Ethiopia Refugee Statistics as of June 2023* 2023 [cited 2023 17.07.2023]; Available from: https://data2.unhcr.org/en/documents/download/101981.
10. Getachew, M., et al., *Magnitude and factors associated with adherence to Iron-folic acid supplementation among pregnant women in Eritrean refugee camps, northern Ethiopia.* BMC Pregnancy Childbirth, 2018. **18**(1): p. 83.
11. UNHCR. *Sharp increase in number of Eritrean refugees and asylum-seekers in Europe, Ethiopia and Sudan*. [cited 2023 10th january]; Available from: https://www.unhcr.org/news/briefing/2014/11/5465fea1381/sharp-increase-number-eritrean-refugees-asylum-seekers-europe-ethiopia.html.
12. International Organization for Migration (IOM), *International Organization for Migration (IOM), Aug 05 2022. DTM Ethiopia — National Displacement Report 12 (March— April 2022). IOM, Ethiopia.* . 2022: Switzerland.
13. VOA, *Report: Eritrean Refugees Relocated from Addis Ababa to Dangerous Area* 2022, VOA.
14. UNHCR. *Ethiopia's Tigray Refugee Crisis Explained*. 2022 [cited 2022 13.12.2022]; Available from: https://www.unrefugees.org/news/ethiopia-s-tigray-refugee-crisis-explained/.
15. UNHCR. *Ethiopia's Tigray Refugee Crisis Explained*. 2022 [cited 2023 30.04]; Available from: https://www.unrefugees.org/news/ethiopia-s-tigray-refugee-crisis-explained/.
16. UNHCR, *Inter-agency Field Manual on Reproductive Health in Humanitarian Settings*. 2010.
17. Gürol, E.C.T.a.S., *The effects of migration on women's health and reproductive health in Turkey.* Frontiers in Women's Health, 2019. **4**.
18. United Nations, D.o.E.a.S.A., Population Division, *International Migrant Stock 2019. (United Nations database, POP/DB/MIG/Stock/Rev.2019).* 2019, IOM: Berlin.
19. Gebrecherkos, K., et al., *Unmet need for modern contraception and associated factors among reproductive age group women in Eritrean refugee camps, Tigray, north Ethiopia: a cross-sectional study.* BMC Res Notes, 2018. **11**(1): p. 851.
20. Ahmed, J.A., et al., *Epidemiology of respiratory viral infections in two long-term refugee camps in Kenya, 2007-2010.* BMC Infectious Diseases, 2012. **12**(1): p. 7.
21. Organization, W.H., *WHO recommendations on intrapartum care for a positive childbirth experience*. 2018: World Health Organization.
22. Darebo, T.D., et al., *The sexual and reproductive healthcare challenges when dealing with female migrants and refugees in low and middle-income countries (a qualitative evidence synthesis).* BMC Public Health, 2024. **24**(1): p. 520.
23. Cignacco, E., et al., *Sexual and reproductive healthcare for women asylum seekers in Switzerland: a multi-method evaluation.* BMC Health Serv Res, 2018. **18**(1): p. 712.
24. IDMC, *IDMC's 2019 Global Report on Internal Displacement*, NRC, Editor. 2019.
25. Chernet, A., *Cardiovascular diseases risk factors among recently arrived Eritrean refugees in Switzerland.* BMC Res Notes, 2019. **12**.
26. Economic, U.N.D.f., S. Information, and P. Analysis, *Population and Development: Programme of Action Adopted at the International Conference on Population and Development, Cairo, 5-13 September 1994*. Vol. 1. 1995: United Nations Publications.
27. UNFPA, *Adolescent Pregnancy: a review of evidence* 2013.
28. Fincher, R.A., *International conference on population and development.* Envtl. Pol'y & L., 1994. **24**: p. 309.
29. Austin, J., et al., *Reproductive health: a right for refugees and internally displaced persons.* Reproductive health matters, 2008. **16**(31): p. 10-21.
30. Health;, M.C.f.W.s., *Act now to a d v a n c e h e a l t h e q u i t y f o r m i g r a n t a n d r e f u g e e w o m e n ' s s e x u a l a n d r e p r o d u c t i v e h e a l t h*. 2021.
31. Usta, J., J.A.M. Farver, and L. Zein, *Women, war, and violence: surviving the experience.* Journal of Women's Health, 2008. **17**(5): p. 793-804.

For peer review only - http://bmjopen.bmj.com/site/about/guidelines.xhtml

32. Barnes, D.M. and C.L. Harrison, *Refugee women's reproductive health in early resettlement.* Journal of Obstetric, Gynecologic, & Neonatal Nursing, 2004. **33**(6): p. 723-728.
33. Bronfenbrenner, U., *The ecology of human development: Experiments by nature and design*. 1979: Harvard university press.
34. Kilanowski, J.F., *Breadth of the socio-ecological model.* Journal of Agromedicine, 2017. **22**(4): p. 295-297.
35. Mengesha, Z.B., et al., *Preparedness of Health Care Professionals for Delivering Sexual and Reproductive Health Care to Refugee and Migrant Women: A Mixed Methods Study.* Int J Environ Res Public Health, 2018. **15**(1).
36. Newbold, K.B. and J. Willinsky, *Providing family planning and reproductive healthcare to Canadian immigrants: perceptions of healthcare providers.* Culture, health & sexuality, 2009. **11**(4): p. 369-382.
37. Ng, C. and K.B. Newbold, *Health care providers' perspectives on the provision of prenatal care to immigrants.* Cult Health Sex, 2011. **13**(5): p. 561-74.
38. King, R., et al., *Barriers and facilitators to accessing skilled birth attendants in Afar region, Ethiopia.* Midwifery, 2015. **31**(5): p. 540-6.
39. Mengesha, Z.B., et al., *Refugee and migrant women's engagement with sexual and reproductive health care in Australia: A socio-ecological analysis of health care professional perspectives.* PLoS One, 2017. **12**(7): p. e0181421.
40. Lazar, J.N., et al., *Providers' Perceptions of Challenges in Obstetrical Care for Somali Women.* Obstetrics and Gynecology International, 2013. **2013**: p. 149640.
41. Fentaw, R., A. Bogale, and D. Abebaw, *Prevalence of child malnutrition in agro-pastoral households in Afar Regional State of Ethiopia.* Nutr Res Pract, 2013. **7**(2): p. 122-31.
42. Dykes, F. and R. Flacking, *Ethnographic research in maternal and child health*. 2016: Routledge Milton Park, Abingdon, Oxon.
43. McLeroy, K.R., et al., *An ecological perspective on health promotion programs.* Health education quarterly, 1988. **15**(4): p. 351-377.
44. Vu, M., et al., *System-level factors influencing refugee women's access and utilization of sexual and reproductive health services: A qualitative study of providers' perspectives.* Frontiers in Global Women's Health, 2022. **3**: p. 1048700.
45. Sawadogo, P.M., et al., *Barriers and facilitators of access to sexual and reproductive health services among migrant, internally displaced, asylum seeking and refugee women: A scoping review.* Plos one, 2023. **18**(9): p. e0291486.
46. Davidson, N., et al., *Access to preventive sexual and reproductive health care for women from refugee-like backgrounds: a systematic review.* BMC Public Health, 2022. **22**(1): p. 403.
47. Tirado, V., et al., *Barriers and facilitators for the sexual and reproductive health and rights of young people in refugee contexts globally: A scoping review.* PloS one, 2020. **15**(7): p. e0236316.
48. Shtarkshall, R.A., F. Baynesan, and B.S. Feldman, *A socio-ecological analysis of Ethiopian immigrants' interactions with the Israeli healthcare system and its policy and service implications.* Ethnicity & health, 2009. **14**(5): p. 459-478.
49. Keygnaert, I., N. Vettenburg, and M. Temmerman, *Hidden violence is silent rape: sexual and gender-based violence in refugees, asylum seekers and undocumented migrants in Belgium and the Netherlands.* Culture, health & sexuality, 2012. **14**(5): p. 505-520.
50. Wells, R., et al., *An ecological model of adaptation to displacement: individual, cultural and community factors affecting psychosocial adjustment among Syrian refugees in Jordan.* Global Mental Health, 2018. **5**.
51. Guruge, S. and N. Khanlou, *Intersectionalities of influence: researching the health of immigrant and refugee women.* Canadian Journal of Nursing Research Archive, 2004: p. 32-47.

For peer review only

For peer review only - http://bmjopen.bmj.com/site/about/guidelines.xhtml

52. White, K., M. Ocampo, and I.C. Scarinci, *A socio-ecological approach for examining factors related to contraceptive use among recent Latina immigrants in an emerging Latino state.* Women & Health, 2017. **57**(7): p. 872-889.
53. Braun, V. and V. Clarke, *Using thematic analysis in psychology.* Qualitative Research in Psychology, 2006. **3**(2): p. 77-101.
54. Gale, N.K., et al., *Using the framework method for the analysis of qualitative data in multi-disciplinary health research.* BMC medical research methodology, 2013. **13**(1): p. 1-8.
55. Finlay, L. and B. Gough, *Reflexivity: A practical guide for researchers in health and social sciences*. 2008: John Wiley & Sons.
56. Tong, A., P. Sainsbury, and J. Craig, *Consolidated criteria for reporting qualitative research (COREQ): a 32-item checklist for interviews and focus groups.* International journal for quality in health care, 2007. **19**(6): p. 349-357.
57. O'Brien, B.C., et al., *Standards for reporting qualitative research: a synthesis of recommendations.* Acad Med, 2014. **89**(9): p. 1245-51.
58. Vlahov, D., et al., *Strategies for improving influenza immunization rates among hard-to-reach populations.* Journal of Urban Health, 2007. **84**: p. 615-631.
59. Korri, R., G. Froeschl, and O. Ivanova, *A Cross-Sectional Quantitative Study on Sexual and Reproductive Health Knowledge and Access to Services of Arab and Kurdish Syrian Refugee Young Women Living in an Urban Setting in Lebanon.* Int J Environ Res Public Health, 2021. **18**(18).
60. Control, C.f.D. and Prevention, *The social-ecological model: A framework for prevention*. 2015.
61. Mengesha, Z.B., et al., *Challenges in the Provision of Sexual and Reproductive Health Care to Refugee and Migrant Women: A Q Methodological Study of Health Professional Perspectives.* Journal of Immigrant and Minority Health, 2018. **20**(2): p. 307-316.
62. Essén, B., et al., *Are some perinatal deaths in immigrant groups linked to suboptimal perinatal care services?* BJOG: An International Journal of Obstetrics and Gynaecology, 2002. **109**(6): p. 677-682.
63. Pottie, K., et al., *Language proficiency, gender and self-reported health.* Canadian Journal of Public Health, 2008. **99**(6): p. 505-510.
64. Horn, R., *Responses to intimate partner violence in Kakuma refugee camp: refugee interactions with agency systems.* Social science & medicine, 2010. **70**(1): p. 160-168.
65. Wright, L.-A. and R. Plasterer, *Beyond basic education: Exploring opportunities for higher learning in Kenyan refugee camps.* Refuge, 2010. **27**: p. 42.
66. Women, U., *The World for Women and Girls Annual Report 2019-2020.* 2020.
67. OBAGBOYE, T.G., *ENTRENCHED EPIDEMIC: STEMMING THE TIDE OF VIOLENCE AGAINST WOMEN IN NIGERIA AND AFRICA.* INTERNATIONAL JOURNAL OF COMPARATIVE LAW AND LEGAL PHILOSOPHY (IJOCLLEP), 2020. **2**(1).
68. Muluneh, M.D., et al., *Gender based violence against women in sub-Saharan Africa: a systematic review and meta-analysis of cross-sectional studies.* International journal of environmental research and public health, 2020. **17**(3): p. 903.
69. Barrett, B.J. and M.S. Pierre, *Variations in women's help seeking in response to intimate partner violence: Findings from a Canadian population-based study.* Violence against women, 2011. **17**(1): p. 47-70.
70. Organization, W.H., *Responding to intimate partner violence and sexual violence against women: WHO clinical and policy guidelines*. 2013: World Health Organization.
71. Chawhanda, C., J. Levin, and L. Ibisomi, *Factors associated with sexual and reproductive health service utilisation in high migration communities in six Southern African countries.* BMC Public Health, 2022. **22**(1): p. 876.
72. Grandey, A.A., A.S. Gabriel, and E.B. King, *Tackling taboo topics: A review of the three M s in working women's lives.* Journal of Management, 2020. **46**(1): p. 7-35.

For peer review only

For peer review only - http://bmjopen.bmj.com/site/about/guidelines.xhtml

73. Traumer, L., M.H. Jacobsen, and B.S. Laursen, *Patients' experiences of sexuality as a taboo subject in the Danish healthcare system: a qualitative interview study.* Scandinavian journal of caring sciences, 2019. **33**(1): p. 57-66.
74. Steven, V.J., et al., *"Provide care for everyone please": engaging community leaders as sexual and reproductive health advocates in North and South Kivu, Democratic Republic of the Congo.* Reproductive Health, 2019. **16**(1): p. 98.
75. Akhavan, S. and S. Karlsen, *Practitioner and client explanations for disparities in health care use between migrant and non-migrant groups in Sweden: a qualitative study.* Journal of Immigrant and Minority Health, 2013. **15**(1): p. 188-197.
76. Canton, H., *United Nations High Commissioner for Refugees—UNHCR*, in *The Europa Directory of International Organizations 2021*. 2021, Routledge. p. 215-234.
77. Seyife, A., et al., *Utilization of modern contraceptives and predictors among women in Shimelba refugee camp, Northern Ethiopia.* PloS one, 2019. **14**(3): p. e0212262.
78. UNHCR, *Deteriorating conditions putting Eritrean refugees at grave risk in Tigray*. 2022, UNHCR: Switzerland

For peer review only

For peer review only - http://bmjopen.bmj.com/site/about/guidelines.xhtml

Figure 1

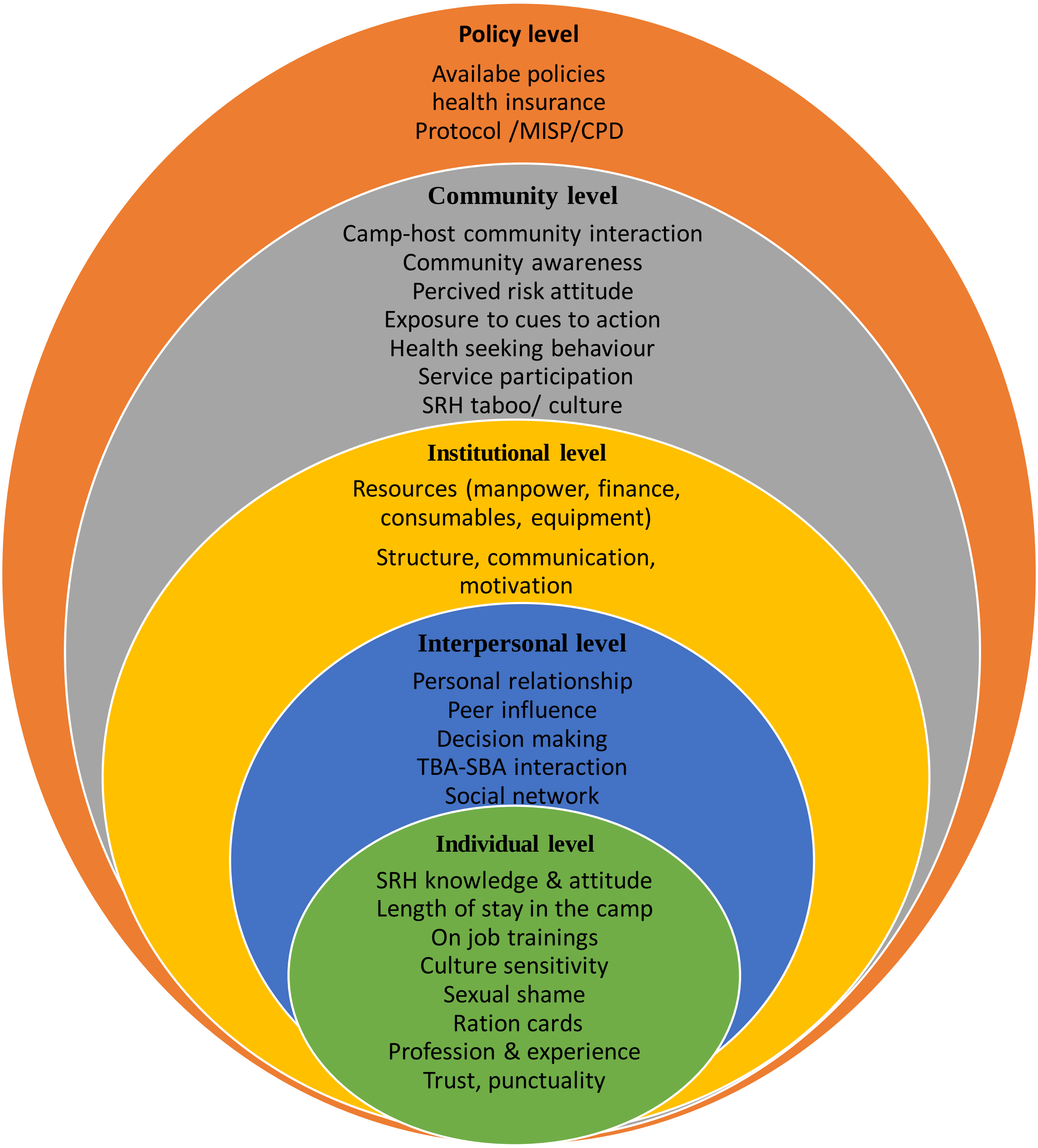

Figure 1. A socio-ecological analysis framework of factors that influence HCPs' readiness to provide SRH services to Eritrean refugee women in Asayta refugee camp, Ethiopia (adapted).

Additional file 3

Standards for Reporting Qualitative Research (SRQR)*

http://www.equator-network.org/reporting-guidelines/srqr/

Title and abstract	Page/line no
**Title** - Concise description of the nature and topic of the study Identifying the study as qualitative or indicating the approach (e.g., ethnography, grounded theory) or data collection methods (e.g., interview, focus group) is recommended	Page# 1, line #1-2
**Abstract** - Summary of key elements of the study using the abstract format of the intended publication; typically includes background, purpose, methods, results, and conclusions	Page# 2, line #32-53

Introduction

**Problem formulation** - Description and significance of the problem/phenomenon studied; review of relevant theory and empirical work; problem statement	Page# 3-5, line #71-151
**Purpose or research question** - Purpose of the study and specific objectives or questions	Page# 4-5, line #151-172

Methods

**Qualitative approach and research paradigm** - Qualitative approach (e.g., ethnography, grounded theory, case study, phenomenology, narrative research) and guiding theory if appropriate; identifying the research paradigm (e.g., postpositivist, constructivist/ interpretivist) is also recommended; rationale**	Page# 5, line #196-199 And Page# 7-8, line #251-299
**Researcher characteristics and reflexivity** - Researchers' characteristics that may influence the research, including personal attributes, qualifications/experience, relationship with participants, assumptions, and/or presuppositions; potential or actual interaction between researchers' characteristics and the research questions, approach, methods, results, and/or transferability	Page# 5, line #221-237 And #299-325
**Context** - Setting/site and salient contextual factors; rationale**	Page# 5, line #175-194
**Sampling strategy** - How and why research participants, documents, or events were selected; criteria for deciding when no further sampling was necessary (e.g., sampling saturation); rationale**	Page# 6, line #202-207 and #214-248

For peer review only

**Ethical issues pertaining to human subjects** - Documentation of approval by an appropriate ethics review board and participant consent, or explanation for lack thereof; other confidentiality and data security issues	Page# 24, line #891-902
**Data collection methods** - Types of data collected; details of data collection procedures including (as appropriate) start and stop dates of data collection and analysis, iterative process, triangulation of sources/methods, and modification of procedures in response to evolving study findings; rationale**	Page# 6, line #213-248

Results/findings

**Synthesis and interpretation** - Main findings (e.g., interpretations, inferences, and themes); might include development of a theory or model, or integration with prior research or theory	Page# 9-19, line #328-721
**Links to empirical data** - Evidence (e.g., quotes, field notes, text excerpts, photographs) to substantiate analytic findings	

Discussion

**Integration with prior work, implications, transferability, and contribution(s) to the field** - Short summary of main findings; explanation of how findings and conclusions connect to, support, elaborate on, or challenge conclusions of earlier scholarship; discussion of scope of application/generalizability; identification of unique contribution(s) to scholarship in a discipline or field	Page# 20-, line #722-805
**Limitations** - Trustworthiness and limitations of findings	Page# 22, line#808-821

Other

**Conflicts of interest** - Potential sources of influence or perceived influence on study conduct and conclusions; how these were managed	Page# 23, line #886
**Funding** - Sources of funding and other support; role of funders in data collection, interpretation, and reporting	Page# 23, line #878-881

For peer review only

KII with health professionals and Key officers

Health professionals in the refugee camp/CH: health professionals treating Eritrean/Somali migrants

Demographic Characteristics of the study participants
Place
Date
Interviewers

ID	Age	Type of the professional*	Experience in camp/center	Religion	Education level	Nationality

***Medical doctor, Nurse, Public health etc.**

For peer review only

1. Survey of the present health status:

- Is a general clinical assessment conducted for women arriving at the camp/asylum centre? If yes, please indicate which kind (physical/psychological/gynaecological)?Where is the clinical assessment usually conducted? Who is usually present during the examination? Is the clinical examination done by a man or a woman? How many persons are present during a gynecological examination?
- Is an interpreter consulted during assessment? If not, why not?
- Is a professional interpreter available?
- How are the confidentiality and privacy maintained when SRH issues are discussed

2. Psychological healthcare:

- Is data on experiences with violence / trauma systematically collected?
- Is an interpreter consulted during this procedure? If not, why not?
- What is the course of action if a current traumatisation is found?
- What should be improved according to you?

For peer review only - http://bmjopen.bmj.com/site/about/guidelines.xhtml

3. Gynaecological healthcare:

- How can an asylum-seeking woman get information on contraception and family planning that she can understand (considering language, literacy) ? How does she get information on consultations?
- Who can a woman go to if she has acute or chronic gynaecological problems (e.g. vaginal infections, bladder infections, menstrual problems, strong or foul-smelling discharge)? How well are women informed about these possibilities? Are they accessing these services, and who supports them in this? Options for improvement?
- Where are prophylactic gynaecological examinations conducted?
- Is an interpreter consulted during the examination? Who is present during the examination?

4. Healthcare during pregnancy:

- How are the pregnancy check-ups organised?
- Is the woman familiarised with different models of healthcare during pregnancy? (e.g. freelance midwife or gynaecologist, hospital care)? Are pregnant women given a choice? How can they get support (e.g. in terms of accompanying, interpreting) if they want to access ANC services?
- Which options (e.g. internet access, information material, prenatal classes) do women have to inform themselves or exchange information about pregnancy in a language they are familiar with?
- Whom can a pregnant woman contact for acute problems and emergencies? If there is no formal contact person, what do women usually do in case of emergencies?
- Which options does a pregnant woman with pregnancy issues (e.g. contractions or back pains) have, so she can be alone?

5. Care during birth:

- How is the transport to the place of child birth organised (public transport, car)?
- Is the pregnant woman accompanied by someone? If yes, by whom? (Partner, friend, acquaintance, relative). In case of an emergency: availability of ambulances? Who covers the cost of an ambulance?

For peer review only - http://bmjopen.bmj.com/site/about/guidelines.xhtml

NIIDS
V2.0_09.02.2021

6. Protection from Violence in the camp/Asylum Centre

- Does your centre have a violence protection concept?
- How are women protected from gender-based violence and sexual assault/harassment?
- Whom can affected women turn to if they feel harassed? Are there any administrative hurdles?
- What supervision, debriefing, or counselling is available for health professionals who treat survivors of violence?

Thank you very much for your time!

For peer review only

For peer review only - http://bmjopen.bmj.com/site/about/guidelines.xhtml

BMJ Open

Navigating Challenges: A socio-ecological analysis of sexual and reproductive health barriers among Eritrean refugee women in Ethiopia, using a key informant approach

Journal:	*BMJ Open*
Manuscript ID	bmjopen-2023-080654.R2
Article Type:	Original research
Date Submitted by the Author:	26-Mar-2024
Complete List of Authors:	Zepro, Nejimu; Swiss Tropical and Public Health Institute, Public Health Epidemiology; University of Basel,<br>Medhanyie, Araya; Mekelle University<br>Probst-Hensch, Nicole ; Swiss Tropical and Public Health Institute, Department of Epidemiology and Public Health; University of Basel<br>Chernet, Afona; Swiss Tropical and Public Health Institute, Department of Epidemiology and Public Health; University of Basel<br>Tschopp, Rea; Swiss Tropical and Public Health Institute, Department of Epidemiology and Public Health; University of Basel<br>Abongomera, Charles; Swiss Tropical and Public Health Institute, Department of Epidemiology and Public Health; University of Basel<br>Paris, Daniel; Swiss Tropical and Public Health Institute, Department of Epidemiology and Public Health; University of Basel<br>Merten, Sonja; Swiss Tropical and Public Health Institute, Department of Epidemiology and Public Health; University of Basel
**Primary Subject Heading**:	Public health
Secondary Subject Heading:	Epidemiology, Global health, Health services research, Qualitative research, Sexual health
Keywords:	PUBLIC HEALTH, QUALITATIVE RESEARCH, REPRODUCTIVE MEDICINE, Health policy < HEALTH SERVICES ADMINISTRATION & MANAGEMENT

SCHOLARONE™
Manuscripts

I, the Submitting Author has the right to grant and does grant on behalf of all authors of the Work (as defined in the below author licence), an exclusive licence and/or a non-exclusive licence for contributions from authors who are: i) UK Crown employees; ii) where BMJ has agreed a CC-BY licence shall apply, and/or iii) in accordance with the terms applicable for US Federal Government officers or employees acting as part of their official duties; on a worldwide, perpetual, irrevocable, royalty-free basis to BMJ Publishing Group Ltd ("BMJ") its licensees and where the relevant Journal is co-owned by BMJ to the co-owners of the Journal, to publish the Work in this journal and any other BMJ products and to exploit all rights, as set out in our licence.

The Submitting Author accepts and understands that any supply made under these terms is made by BMJ to the Submitting Author unless you are acting as an employee on behalf of your employer or a postgraduate student of an affiliated institution which is paying any applicable article publishing charge ("APC") for Open Access articles. Where the Submitting Author wishes to make the Work available on an Open Access basis (and intends to pay the relevant APC), the terms of reuse of such Open Access shall be governed by a Creative Commons licence – details of these licences and which Creative Commons licence will apply to this Work are set out in our licence referred to above.

Other than as permitted in any relevant BMJ Author's Self Archiving Policies, I confirm this Work has not been accepted for publication elsewhere, is not being considered for publication elsewhere and does not duplicate material already published. I confirm all authors consent to publication of this Work and authorise the granting of this licence.

For peer review only

Navigating challenges: a socio-ecological analysis of sexual and reproductive health barriers among Eritrean refugee women in Ethiopia, using a key informant approach

*Nejimu Biza Zepro [1, 2, 3] nejimu.zepro@swisstph.ch
Araya Abrha Medhanyie [4] araya.medhanyie@gmail.com
Nicole Probst-Hensch [1, 2] nicole.probst@swisstph.ch
Afona Chernet[1, 2] afona.chernet@swisstph.ch
Rea Tschopp[1, 2] rea.tschopp@swisstph.ch
Charles Abongomera[1, 2] charles.abongomera@swisstph.ch
Daniel Henry Paris [1, 2] daniel.paris@swisstph.ch
Sonja Merten[1, 2, 5] sonja.merten@swisstph.ch

[1] Department of Epidemiology and Public Health, Swiss Tropical and Public Health Institute, Allschwil, Switzerland
[2] University of Basel, Basel, Switzerland
[3] College of Health Sciences, Samara University, Afar, Ethiopia
[4] School of Public Health, College of Health Sciences, Mekelle University, Tigray, Ethiopia
[5] Center for African Studies, University of Basel, Basel, Switzerland

*Corresponding author: nejimu.zepro@swisstph.ch or nejim76@gmail.com

For peer review only - http://bmjopen.bmj.com/site/about/guidelines.xhtml

Abstract

Objectives The study aimed to explore the experiences and perceptions of healthcare providers (HCP) regarding the sexual and reproductive health (SRH) challenges of Eritrean refugee women in Ethiopia.

Design A qualitative exploratory design with the key informant (KI) approach.

Setting and participants The study was conducted in the Afar regional state, North East, Ethiopia. The study participants were HCP responsible for providing SRH care for refugee women.

Results Eritrean refugee women have worse health outcomes than the host population. The SRH needs were found to be hindered at multiple layers of Socio-Ecological Model (SEM). High turnover and shortage of HCP, restrictive laws, language issues, cultural inconsistencies, and gender inequalities were among the main barriers reported. Complex multi-structural factors are needed to improve SRH needs of Eritrean refugee women.

Conclusions A complex set of issues spanning individual needs, social norms, community resources, healthcare limitations, and structural mismatches create significant barriers to fulfilling the SRH needs of Eritrean refugee women in Ethiopia**.** Factors like limited awareness, cultural taboos, lack of safe spaces, inadequate healthcare facilities, and restrictive policies all contribute to the severe limitations on SRH services available in the refugee settings. The overlap in findings underscores the importance of developing multi-level interventions that are culturally sensitive to the needs of refuge women across all SEM levels. A bilateral collaboration between Refugees and Returnees Service (RRS) structures and the Asayta district healthcare system are critically important.

Data availability statement: All data relevant to the study are included in the article or uploaded as supplementary information.

Keywords: Eritrea, Ethiopia, health care providers, humanitarian crises, key informant research, refugees, sexual and reproductive health, socio-ecological framework

Strengths and limitations of this study

- Key informant researcher (KIR) approach is found to be appropriate to explore super sensitive SRH issues such as sexuality, multiple sex partners, and intimate partner violence.
- Qualitative study design allowed for a more detailed and nuanced contextual understanding of the SRH challenges among refugee women in such fragile hard to reach settings.
- Collaboration with an experienced social science researcher, and local institutions was instrumental in building relationships and trust between the research team.
- Comparing results with other sites may be difficult considering that the study was conducted with a small sample of HCP in a defined humanitarian settings.

For peer review only

Introduction

Migration health is a growing global public health issue that should be addressed in line with the United Nations (UN) 2030 Sustainable Development Goals (SDG), particularly Goal 10, reducing inequalities, goal 5, gender equity and Goal 3, good health and well-being for all [1]. However, the public health priorities during migration on sexual and reproductive health (SRH) needs are often overlooked by the health system, with staggering consequences [2]. The World Health Organization (WHO) advocates for universal health coverage, leaving no one behind. This includes the most vulnerable populations including refugees [1]. WHO has also recognized SRH care access as a global health priority in every setting [3].

Previous studies have shown that SRH service needs of refugee women are often prone to poor access to SRH key services, risky life-threatening complications, traumatic events to serious mental illness, unmet need to contraceptives, unplanned pregnancy, unsafe abortion, sexual gender-based violence (SGBV), and sexually transmitted infections (STI) [4-6]. Patriarchy and violence also impacted SRH of Rohinga refugee women in Bangladesh [5, 7]. The United Nations Higher Commission for Refugees (UNHCR) estimates that forced migration has increased from 42.8 million in 2012 to 101.1 million in 2022 as a result of persecution, hostilities, conflict, violence, abuses of human rights, and significant disruptions to the public order [8].

Ethiopia is the second most populous country in Africa (over 110 million people) and the third-largest refugee-hosting African country, sheltering 926,000 refugees and asylum seekers as of 13 June 2023, mainly from South Sudan, Somalia and Eritrea [9-11]. In addition, a total of 2.8 million internally displaced persons (IDP), excluding IDP from the Tigray region, have been identified [12]. Over 140,000 Eritreans who fled hardship and persecution in their home country are hosted in Ethiopia [13]. Some 96,223 Eritrean refugees lived in four refugee camps in the northern part of Ethiopia, Tigray region [14]. However, the two year civil war that erupted in the region in November, 2020, affected the area including these camps, and triggered a massive exodus of local civilians and Eritrean refugees to Sudan, and internally to a nearby Afar region, which created a huge humanitarian crisis. The Afar region hosts nearly 300,000 IDP due to the war in Tigray. IDP have settled in the collective sites with host communities. More than 4,000 IDP live in Semera, and another 10,000 live in Afdera town [15]. This all creates a major burden on an already overburdened country [16].

Migration negatively impacts everyone, particularly women, girls, and children are the most vulnerable groups [17]. Nearly half, 48.4% and 46%, of the world's migrant population are women and children, respectively [8, 18]. Refugee women face a double burden; in addition to the hardships of forced migration, they suffer from challenges related to their gender. They are at increased risk for sexual abuse, rape, unwanted pregnancies, unsafe abortions, and the spread of sexually transmitted infections, including HIV, and short birth interval, which lead to long-term mental health problems and high infant and maternal mortality [19-22]. During the collapse of existing social systems, women are unable to care for their children and themselves. In host countries, migrant women usually bear full responsibility for family members and child care, but at the same time do not have access to resources and the ability to make autonomous decisions about them to the same extent as their male counterparts [22-25].

For peer review only - http://bmjopen.bmj.com/site/about/guidelines.xhtml

For this study, the definition of SRH was adopted from the International Conference on Population and Development (ICPD 1994) and The United Nations Population Fund (UNFPA), as it refers to a state of complete physical, mental and social well-being in all matters relating to the reproductive system. It implies the ability to be free from unwanted pregnancy, unsafe abortion, STI including HIV, prevention of violence against women, elimination of harmful traditional practices and all forms of sexual violence, [26]. SRH is at the heart of human existence, prosperity, and well-being of individuals and societies worldwide [27]. SRH is a core component of primary health care, a universal human right, that applies to every humankind including refugees, internally displaced people and, asylum seekers, but is often forgotten [7, 16]. To realize this universal right, all affected groups must have access to SRH services so they are free to make informed choices and decisions [1, 16]. SRH is a basic human right and an essential element in achieving health, gender equity, and social justice. Women's rights to SRH were recognized as fundamental human rights at the ICPD 1994 in Cairo and goes beyond refugee camps and other humanitarian emergencies [28-30]. Refugee women continue to experience poorer SRH outcomes than the general population, and refugee women are over-represented in the numbers of poor SRH outcomes [30-32].

The socio-ecological model (SEM) is a theoretical framework that suggests individual behavior and health outcomes are influenced by a combination of individual, interpersonal, social, organizational and environmentally complex interrelated factors. This model emphasizes one interconnectedness of these factors and the need to consider them holistically by addressing public health issues. Urie Bronfenbrenner (1979) first introduced the SEM model as a conceptual framework for comprehending human growth. In the 1980s, the model was organized as a theory [33]. The modified SEM in this study seeks to comprehensively assess individual, community, organizational, and societal levels [34]. These five levels of SEM are highly interconnected [35]. Health Care Providers (HCP) themselves experience challenges at all five levels of the SEM framework. Women's past experiences about contraceptives may present challenges to use SRH services at the individual level [36, 37]. On the interpersonal level, lack of cultural competence of health care staff and communication difficulties must be considered [36]. Organizational level challenges were women's unfamiliarity with the healthcare system, a lack of training and protocols governing the delivery of healthcare.

Although Ethiopia's healthcare system is still developing, SRH care is provided free of charge as part of the public service at the primary care unit. Services are mainly provided in the Maternal and Child Health (MCH) Unit in health centers, health posts, and district hospitals. Pregnancies with complications or at risk of complications are treated in hospitals as part of specialized health care. Mainly midwives and nurses provide their services independently in health centers.

SRH challenges are recognized from the end-user (refugee) perspective; there is a lack of research on HCP perceptions and experiences of interacting with refugee women seeking SRH care. The perspectives of HCP are equally important, as they often deal with, provide care to, and support refugees. HCP are key informant researchers as they are in a better position to tell what challenges and opportunities the health system poses to the refugees [38]. Moreover, since refugees can't tell or inform the barriers and opportunities that they may have from the health system, they also play a role in helping refugees navigate the healthcare

For peer review only - http://bmjopen.bmj.com/site/about/guidelines.xhtml

system and access the services they need [35]. However, only few researchers have looked at SRH needs of refugee women from HCP's perspectives [36, 37, 39, 40]. Thus, we want to uncover how different broad-level ecological factors that impact women's SRH. Therefore, a systematic approach that considers influences that go beyond the individual level is relevant to gain a broader understanding of health service utilization [35]. This study examined the difficulties of HCP when caring for refugee women and examined how these difficulties affected the provision of SRH care.

Findings from the study could help RRS agency to improve SRH service provision for refugees and migrants. Likewise, other major stakeholders like the United Nations (UN), United Nations High Commissioner for Refugees (UNHCR), World Food Program (WFP), International Medical Corps (IMC) Goal Ethiopia, , and others will benefit from the study findings. This will also allow for exploring the possibility of updating and standardizing service and training packages for SRH service providers to improve comprehensive SRH services provisions in similar humanitarian settings. Thus, understanding the magnitude of the problems and the reasons behind them is crucial for program planners to design effective preventive strategies.

Methods

Study setting

The study was the qualitative arm of the Novel Integrated Infectious Disease Surveillance (NIIDS) project carried out in Ethiopia and Switzerland. The study was conducted in the Asayta refugee camp in Afar, North East part of Ethiopia, which currently hosts more than 18, 000 Eritrean refugees. The district is characterized by an arid climate and an ecologically fragile ecosystem, making it difficult to provide basic health care and life-saving services. It is located at 65 km South West of Semera (administrative capital) and 649 km from Addis Ababa, the capital city of Ethiopia, and shares an international border with Djibouti. The temperature varies between 30 and 45 degrees Centigrade, the altitude ranges between 350 and 500 meters above sea level, and the rainfall is irregular. The total population of the district is estimated at 47,210 where (66% live in rural areas). They rarely use health care facilities, mainly because they do not know where to get health care and access to services is difficult [41]. There are four private clinics, three public health posts, and one public district hospital. Malaria, diarrhea, and other febrile illnesses are quite common in the district due to inadequate clean water and sewage disposal facilities, inadequate availability of health facilities, low supplies, and delays in the distribution of humanitarian relief supplies [41].

Asayta refugee camp, which is located 2 km outside Asayta town, is administered by the RRS in collaboration with UNHCR. The refugee clinic is located inside the camp that provides primary health care to Eritrean refugees. Severe illnesses that require advanced diagnostics are referred to the nearby Asayta district hospital.

Study design

An exploratory qualitative study design with a Key Informant Research (KIR) approach was applied [42]. Semi-structured individual interviews were conducted to explore the experiences and perceptions of HCP in providing SRH care for Eritrean refugee women.

For peer review only - http://bmjopen.bmj.com/site/about/guidelines.xhtml

Study participants

Study participants were HCP responsible for providing SRH care to Eritrean refugee women in the Asyta refugee camp. HCP were purposely selected because they are particularly aware of SRH needs and service uptake among Eritrean refugee women. The perspectives of HCP are equally important in research. By sharing their knowledge and experience, HCP can help ensure that research studies are relevant, culturally appropriate and feasible in the context of the refugee women being studied.

Patient and public involvement

No patients were involved. Key informants were not involved in the design, conduct or analysis of this paper. However, we are planning to hold a dissemination workshop on the project's key findings and consider future follow up projects which will result in a long-term partnership with the Asayta refugee camp.

Data collection tool and participant recruitment

We developed a separate semi-structured initial qualitative interview guide based on a review of the relevant literature [43-46]. Study participants were requested to mention their professional background, experience, health facility responsibilities, and the proportion or number of refugee women they serve each month. Participants were then asked a series of open-ended questions about their experience in providing SRH care for Eritrean refugees.

Data was collected through Key Informant Interviews (KII), and focused observations. The initial interview guide was developed to comprehensively capture a core set of questions from all participants. Further questions were posed depending on the type of occupation and organization in which the interviewees worked. The KII guide included general questions about the SRH needs of women as perceived by HCP during the delivery of SRH services, the accessibility of SRH services, the challenges women and HCP face during treatment, and recommendations on how to mitigate these challenges 'supplementary file'. To increase the credibility and richness of the data, respondents were intentionally selected to be as diverse as possible in terms of work experience, gender, work location, and cultural background. Field notes were taken to supplement the audio recordings and document nonverbal cues. At the end of all sessions, the lead author debriefed the participants, summarizing basic information.

The first author conducted the KII with the support of a trained research assistant who had no prior contacts or relationships with study participants. Before the interviews began, a researcher explained the detailed objectives of the study to the participants and obtained their written and verbal consent to conduct the interview. Study participants signed a written informed consent form, read the confidentiality statement, and were reminded of their voluntary participation and their right to terminate the interview at any time. The data collection period was between July and August 2022.

Interviews were conducted face-to-face in the Amharic language (Ethiopia's official language). Interviews were recorded and transcribed verbatim. Data saturation was reached when no more new information continued to emerge. A summary report of each interview was also written. After the transcriptions were finished, the audio files were removed from each transcriber's personal computer. The interviews were professionally transcribed by the first author with the assistance of an experienced female qualitative researcher. The transcriptions

For peer review only - http://bmjopen.bmj.com/site/about/guidelines.xhtml

were translated into English for further analysis. On the study materials or transcripts, there were no names or other identifiable personal information. COVID-19 protection measures were taken during interviews, including use of hand sanitizers, use of face masks, social distancing, and conducting interviews in ventilated large rooms. To maintain anonymity and confidentiality, all study participants were identified with a code.

Data analysis

The audio recordings were listened to several times to confirm the completeness of the verbatim transcription, identify contextual meanings, and make preliminary interpretations of the data. This yielded sufficient textual data to perform iterative categorization of the data for better analysis. The coding process helped us to identify common themes. Codes with similar meanings were combined and then sorted to form subcategories. The subcategories were then compared, and categories with similar concepts were combined into one category.

Due to the complexity of SRH topics, especially in such groups of women, we conducted a systematic two-stage analysis. First, we conducted a thematic analysis of the interviews. Second, taking into account the broader nature of the research question, we developed an adapted SEM model to provide a clear framework and recommendations for improving the SRH needs of Eritrean refugee women. We opted for this model because it effectively illustrates the complex interplay among various factors influencing the health and well-being of refugees[22, 47]. These factors encompass individual aspects such as behaviors, social and community elements like norms, institutional and policy components such as the healthcare system, as well as structural factors including policy and protection. The SEM model has been used elsewhere to understand SRH barriers for refugees. For example, Shtarkshall et al, and Mengesha et al, applied this model to examine refugee and migrant healthcare access issues in Israel and Australia [39, 48]. Mengesha and colleagues [39], Keygnaert and colleagues [49], Tadele and colleagues [22], and Wells and colleagues [50] have used the SEM framework as well. This model proved to be particularly relevant because it focuses on the synergistic relationship between individuals and their social environment [33, 39]. The model also describes the multiple interactions among individual (micro), interpersonal (meso), institutional (exo), and societal (macro) factors that influence health seeking behaviors [22, 39, 47].

Computer-assisted qualitative and mixed-methods data analysis software (MAXQDA 2018) was used to organize codes and themes to support analysis through close reading of transcripts and notes. Common themes were discussed further by the authors for relevance and consistency with the SEM framework. The use of SEM is justified by the fact that refugee women's use of SRH care is influenced by multiple dynamic factors that consider the interactions between different levels of SEM [22, 51, 52]. Again, it is the interactions of refugee women with their environment, at individual, interpersonal, community, societal, institutional, and policy levels, that can influence access and utilization of SRH care by refugee women, as well as SRH care provision by HCP in Ethiopia [51]. The data transcripts were then analyzed using the Gale *et al*, framework method, drawing from Braun and Clarke's thematic analysis [53, 54]. The analysis steps suggested by Gale *et al*. were modified and applied as follows: 1) audio transcription and translation; 2) reading and rereading the translations to

For peer review only - http://bmjopen.bmj.com/site/about/guidelines.xhtml

become familiar with the interviews; 3) line-by-line coding using MAXQDA; 4) developing a thematic framework; 5) applying the framework; 6) placing the data into an SEM framework; and 7) interpreting the data [54].

Inductive approach was used to identify codes and themes from the data. Analysis began with a familiarization to the developed transcripts to assign initial codes. The data coding was done by using MAXQDA, and integrity was double-checked. A summary of the coded data set was then created with specific reports to identify initial themes. This process allowed to define core concepts and identify unique and specific stories for each theme. Finally, SEM was applied to identify and interpret factors that influence SRH care among Eritrean refugee women at the individual, interpersonal, institutional, societal, and policy levels.

Data quality

We took part in a reflective process relevant to the dynamics between the researcher and the researched by examining critically how our social and cultural backgrounds, assumptions, and positions impacted the study process [55]. Researcher reflexivity was ensured through additional field notes, focused observations, informal conversations with concerned stakeholders, and peer debriefings after each interview. Data dissemination and reporting was guided by the Consolidated Criteria for Reporting Qualitative Research (COREQ) and the Standards for Reporting Qualitative Research (SRQR) guidelines 'supplementary file' [56, 57]. Rigor and trustworthiness were assessed using the principles of credibility, reliability, confirmability, and transferability. Data credibility was enhanced through prolonged engagement, peer debriefing, written reminders, and various bracketing methods for data collection. A complete and continuous data collection method was used to ensure data reliability. The data were also reviewed by an expert researcher who had no connection to the study and was an outside observer. To confirm the data, the entire research process was recorded. Also, research colleagues who were not involved in the study but were knowledgeable about qualitative data analysis were given access to the transcripts of interviews as well as the extracted codes and categories to confirm the validity of the coding procedure. Finally, to ensure the transferability of the data, results were examined from several individuals who had similar characteristics to the participants in the study but did not participate in the present research. Data triangulation was achieved through a combination of different data sources (interviews, field notes, focused observation, and SEM theory).

The research team's interdisciplinary makeup allowed for a high level of reflexivity, which pushed us to investigate and consider assumptions and biases. All the authors have ample experience with qualitative studies, and two of the authors (SM and AAM) are recognized experts in the field of SRH in fragile humanitarian settings. We looked at how closely the retrieved themes and theme clusters matched the SEM categories. Almost 72% of the codes and themes developed were in agreement with those taken from the SEM perspective and applied by other researchers [35, 39, 48]

For peer review only

For peer review only - http://bmjopen.bmj.com/site/about/guidelines.xhtml

Results

Ten KIIs took part in the study, with six from the refugee camp and four from the host hospital. Among them were five midwives, four nurses, and one senior manager. The duration of the interviews ranged from 30 to 72 minutes. The results of the study are presented thematically. The various codes that emerged were classified into several categories and subcategories. The overlapping and similar categories and sub categories were grouped into five main themes. Finally, we found that many themes overlapped at multiple levels of the SEM framework, indicating multilayered influences of complex issues.

Socio-demographic characteristics of the respondents

The total of ten HCP participated in the study with a gender balance of 5 males and 5 females. The age range of participants was between 20 to 40 years, indicating that most of the study participants were young employees. The minimum education level was a college diploma, while more than half of the participants had a bachelor's degree. All participants except two were not from Afar region. Most of them had a total of more than three years of work experience, while the rest had been on the job for only two or three years. The experience of working in Asayta refugee camp is quite limited with a maximum of one year. We cannot list the study participants here, as they are few and easily identified.

Themes and categories according to SEM

The analysis of the main findings of the present study relating to the SRH needs of Eritrean refugee women was explored by thematically mapping the problems to five SEM levels: individual, interpersonal, organizational, community, and larger policy perspectives. The intersecting rings in the model show how factors at one level influence factors at other levels (Figure 1 below).

Individual/intra personal-level factors

The first SEM level, individual level, considers personal characteristics such as age, gender, knowledge, culture, beliefs, and behavioral and lifestyle factors. Job category, camp experience, gender, ethnicity, religion, marital status, SRH knowledge, displacement experiences, and cultural taboos were identified as individual factors that impede appropriate SRH service delivery. However, Eritrean refugee women were strongly influenced by their SRH knowledge, autonomy, cultural beliefs, challenges from HCP, and stressors of adapting to a new environment.

Refugee women's SRH knowledge

A lack of clear and accessible health information is mentioned as a barrier to accessing SRH care. HCP noted that SRH services are not adequately advertised in local languages as well as refugee's mother tongue, leading to confusion among women, particularly Eritrean refugee women who have limited literacy skills, or are unfamiliar with the health system.

It may be helpful to consider strategies to increase accessibility and visibility of SRH services in the refugee camp, such as using multiple communication methods (e.g., visual art, outreach), providing information in local languages, and clearly labeling the location of services.

For peer review only - http://bmjopen.bmj.com/site/about/guidelines.xhtml

It was explained that the refugee camp seeks to provide health education on a range of SRH topics, including STI, contraceptive methods, prenatal care, obstetric care, postpartum care, and gender-based violence. Women must have access to accurate and relevant information about SRH issues so they can make informed decisions about their health.

However, women generally have a limited understanding of SRH issues. Women turn to multiple people for information on SRH issues, and HCP report having to spend a great deal of time explaining and persuading women. This could be due to several factors, including cultural beliefs and practices, lack of access to education, and lack of exposure to SRH information in addition to language barriers.

> "Eritrean refugee women's SRH knowledge is quite limited. We spend a lot of time educating refugee women about SRH issues because this information is not a high priority as there are more pressing issues for refugee women, such as housing, food, asylum applications, and social issues. They want clear and complete information about prenatal care, pregnancy, and childbirth. They also want to know how maternity issues might affect their asylum claim." KII age 20-30 years.

Another HCP elaborated her critical observation as follows:

> "Awareness among refugee women is limited. There is a need for "repeated SRH counseling sessions" to build trust in SRH care. I believe that women use SRH services not because of better health behaviors, but because of the food supply and the camp's refusal to register children born outside the camp clinic." KII age 30-40 years.

HCP are reluctant to provide safe abortion services

Eritrean refugee women in need of abortion often turn to private clinics for abortion services. The HCP at the hospital said:

> "Oh yes, we have to recognize Ethiopian abortion law.' So what's happening on the ground seems to be less official policy and more 'cultural/religious resistance' to safe abortion services". KII age 20-30 years.

Safe abortion services are generally believed to be not available due to the reluctance of HCP. While most private clinics offer clandestine abortion service, the cost is prohibitive for refugee women and the skills of the professionals in private clinics are not reassuring. As a result, refugee women often opt for illegal, self-induced abortions which are likely to be unsafe and put women at risk of severe bleeding. HCP interviewed reported that refugee women with complications following unsafe abortions are admitted to public hospitals.

> "I have never seen a refugee woman come here (to the refugee clinic) to have an abortion. They do it themselves, self-induced. They buy abortion pills from unknown sources. It is also common to see schoolgirls from the refugee camp buying abortion pills to end a pregnancy. These drugs are purchased without a doctor's prescription. They even have their traditional methods, like using certain traditional medicines, and they come to us with heavy bleeding. So by the time they come to us, the pregnancy has already been terminated. So we had to refer them to Asayta Hospital, where they

For peer review only - http://bmjopen.bmj.com/site/about/guidelines.xhtml

receive better treatment like D&C [dilation and curettage]. Curettage removes anything that is left behind." KII age 20-30 years

Women's autonomy

Autonomy refers to a woman's power to make decisions about her SRH services free from coercion and violence. Women's self-decision-making power enables individuals, especially marginalized or disadvantaged groups, to have more control over their own lives. For refugee women, autonomy can involve a range of activities aimed at increasing their voice and decision-making power.

> "Refugee women who arrived at the camp reported having very limited autonomy in sexual and reproductive health issues. I think this is due to limited literacy skills, poor understanding of health issues, inability of HCP to speak the local language, and limited social networks, usually competing with competing childcare responsibilities in large families with six to eight children in a household." KII age 30-40 years

Women's autonomy could be strengthened through economic empowerment. However, as a KI participant explained below, it is less likely to be pragmatic, giving the environmental and economic landscape of the region in favor of refugees.

> "It is unlikely that the refugees will be able to find a job here. They usually go far to collect firewood in the grassland, making cultural items such as mats and grass rugs, which are used for prayers, raising goats for sale, or working as wage laborers in the construction sector, and factories. They usually work hard to earn little money to buy grains of wheat to feed their children." KII 30-40 years.

Women's empowerment

The empowerment of women is crucial for unlocking their full potential. Within refugee camps, empowerment is not only a moral imperative but also vital for the overall well-being and stability of the camps. Empowered women can more effectively nurture their families, actively engage in community development, and pave the way for a brighter future for themselves and their children. Unfortunately, Eritrean refugee women lack opportunities to access programs promoting women's empowerment.

> "The refugee women are not interested in applying for social assistance, preferring to find suitable work to meet their family's basic needs. They describe life in the refugee camp as a modern prison, even though the security situation here is much better than in Eritrea." KII age 30-40 years.

Health seeking behavior

The SRH needs of both the refugees and women in the hosting communities found to have many similarities. Among these are having similar SRH needs related to accessing SRH services, cultural/traditional expectations and engaging in harmful traditional practices. However, refugee women were found to have additional SRH needs related to issues of referral systems, gender-based violence, women's empowerment and limited resources in refugee camps. Even though refugees and local people share common sociocultural standards

For peer review only - http://bmjopen.bmj.com/site/about/guidelines.xhtml

and behaviors, refugee women have better health seeking behavior that differs significantly from women in the host community.

Interpersonal-level factors

The second level of the SEM perspective examines the relationships that prevent women from using SRH services. The main SRH barriers reported consistent with this SEM level were: language barriers, cultural concerns, interactions with others, and peer pressure that may provide social support or create barriers that may influence a woman's perception of healthy behaviors.

Language and cultural barriers

> "The first and most common challenge for all of us in here and in the community is the language problem. There is no language school or dictionary to learn the local language (Afaraf). I have tried to learn common words to help the women in their mother tongue, but I am not fluent enough to talk to the women and treat them according to their needs. All the women here want to be treated holistically, in the context of their religious, cultural, and traditional values." KII age 30-40 years.

HCP expect their clients to be able to understand (Amharic). If this is not the case, they are expected to bring translators. Clients, on the other hand, especially newly arrived refugees, may not be aware of this problem. Instead, they expect services to be provided in their native local language (Afaraf) and that a translator will be available if HCP do not speak the language.

> "The majority of refugee women in the camp do not speak Amharic, and those with whom we cannot communicate have limited (or no) access to SRH care, even when they come and sit in front of us. The cultural and language barrier prevents refugee women from openly expressing their needs because they are not able to clearly state their needs. That's what we find so unfortunate about the language problem in the clinic." KII age 20-30 years.

A KII at the camp, described the frustration with translators and how SRH care for adolescents' needs could be compromised because only one interpreter was available at the refugee clinic. Thus, relatives or family members usually assist with translation. The involvement of relatives as interpreters in health care may raise ethical concerns regarding privacy and confidentiality of the services provided.

> "Adolescent refugees are the main users of contraceptives. This is often influenced simply by the presence of a translator who is a member of the refugee community. Adolescents are embarrassed to talk about SRH issues and contraceptives when she (translator) is present. They prefer to call my cell phone for private counseling." KII age 20-30 years.

HCP are accused of showing insensitivity to the cultural practices and religious beliefs of refugee women, and of assuming that the way they treat them meets global scientific standards. This insensitivity is expressed in them saying:

For peer review only - http://bmjopen.bmj.com/site/about/guidelines.xhtml

"Unfortunately, we "male HCP" continue to do what we have trained for: 'frequent touching' and 'naked exams.' However, many refugees assume they can be guaranteed a female HCP to address sensitive SRH needs, especially during childbirth." KII age 30-40 years

Peer pressure

Peer influence can be a powerful factor in shaping the SRH needs of refugee women. This happens when individuals feel pressured to conform to the norms, values, or behaviors of the group in order to fit in or be accepted.

> "We experience that refugee women are often influenced by their peers. This peer pressure may result in positive or negative effects on SRH decision-making. It can also be helpful to seek support from trusted friends, family members, or health care providers when making health behavior decisions." KII age 20-30 years.

Institutional level factors

The third SEM level on institutional factors, HCP reported several problems impeding SRH care that are attributable to the organizational level. These bottlenecks relate to the physical work environment, duties and responsibilities, incentives, overtime pay, laboratory facilities, medications, supplies, professional development plan, organizational communication, and decision-making. Unavailability of HCP and essential services such as a central data clerk, laboratory, and pharmacy, poor quality of services, long wait times, and prohibitive transportation costs for life-threatening emergencies were the barriers frequently reported to camp officials.

High staff turnover

HCP cited several reasons for the high turnover in the Asayta refugee camp. These include: difficult working conditions, inadequate shelter, harsh living conditions, and traumatic experiences. Other factors that may contribute to high HCP turnover include: low job satisfaction, inadequate supervision, and limited opportunities for professional development. The high turnover of HCP was mentioned as one challenge to establishing sustainable healthcare in the camp. Major collapses in care delivery were during the war in the northern Ethiopia where the camp was overwhelmed by a large number of refugees. This high turnover worsens during the pandemic. A study participant stated:

> "It is difficult to convince RRS administration to accept new practices. It takes time to convince them, and on top of that, key leaders change frequently. The leader you convinced and who accepted your proposal is soon replaced, and the new leader needs another time to understand your plan, which is the biggest challenge to staying on the job. This is extremely concerning in our professional duties. To address the high turnover among HCP in refugee camps, it may be helpful to address the root causes of this problem and implement strategies that improve working conditions, increase job satisfaction, and provide opportunities for professional development and advancement. This could include providing adequate resources and support for HCP, improving housing and living conditions, and offering CPD opportunities." KII age 30-40 years.

For peer review only - http://bmjopen.bmj.com/site/about/guidelines.xhtml

Fragmented healthcare system

HCP employed in refugee structures function independently of the regional (district) healthcare system and are structurally responsible for the federal RRS. The refugee clinic lacks essential laboratory equipment and supplies, which can prevent refugee women from accessing SRH services. As a result, the clinic often refers its patients to other hospitals, including Asayta Hospital, Dubti Hospital, and other specialized hospital in Addis Ababa. This referral process is lengthy and cumbersome and can be challenging for patients seeking treatment, especially if they have limited transportation or financial resources. The lack of essential supplies affects the ability of health workers to make accurate diagnoses and treat patients and can lead to delays in treatment and negative health outcomes.

> “Most of the patients are Eritreans because they have no other options to seek treatment and rely on the referral chains in the camp, which are too long and cumbersome. The lack of basic laboratory materials affects the diagnosis and treatment of clients and can lead to delays in care and negative health outcomes.” KII age 30-40 years.

Another HCP provider expressed the frustration with the complex challenges of the referral system, emphasizing the difficulties of SRH services for a women from remote locations:

> “The referral system in place in Asayta refugee camp may not be accessible or practical for women living in remote or hard-to-reach areas, such as "way up" in the mountains and off the road. In these cases, it is difficult or impossible for ambulances to reach these women, instead, mothers in labor may have to be carried to the hospital by others, which can be physically and emotionally exhausting and increase the risk of complications for both the mother and the baby.” KII age 20-30 years.

Community level factors

The fourth level of SEM focuses on the broader community and societal factors that may impact the SRH needs of Eritrean refugee women living in camps and host communities. These factors include the cultural, social, and physical norms that prevail in camps and host communities, as well as the physical and social environments in which people live and work. SEM also looks at the perceived social value of HCP and the perceived stigma associated with seeking health services, as well as satisfaction with local, state, and national government policies. In this study, community-level influences on the SRH needs of Eritrean refugees that emerged included factors within the camp setting, such as informal networks, e.g. community leaders, and the availability of community resources and interactions with the host community. All of these factors can impact refugees' ability to access the SRH services they need.

SRH is a taboo topic

It is not uncommon for certain cultural or religious practices to influence attitudes toward SRH issues. Among groups of Eritrean refugees, these practices are seen as incompatible with cultural or religious beliefs and are considered offensive or inappropriate. This makes it

For peer review only - http://bmjopen.bmj.com/site/about/guidelines.xhtml

difficult for refugee women to access SRH services or make informed decisions about their health and can lead to negative consequences such as social exclusion or stigmatization.

HCP explained how SRH issues are very much taboo topics:

> "SRH issues are taboo topics that are rarely talked about within the family circle." KII age 20-30 years.

Another HCP added:

> "Even the thought of SRH services, contraceptives, sex before marriage, and abortion is considered "harmful to religion and culture" because culture and religion dictates remaining virginal until marriage. Adolescents who have premarital sex are considered guilty and can be excluded from the family and society." KII age 30-40 years.

Community perceptions of SRH services can have a significant impact on the uptake of these services. In Afar communities, there are many negative attitudes or stigmas surrounding SRH services, which can make it difficult to access these services. Women view SRH services as inappropriate or immoral, or they may have cultural or religious beliefs that conflict with accessing these services. This can lead to a lack of support for SRH services within the community and make it difficult for people to access these services even when they are available. On the other hand, in communities where SRH services are viewed positively and supported by community leaders and other influential figures, these services will likely be more widely available and more widely used. HCP we spoke with explained that refugee women and especially young adolescents have difficulty accessing SRH services for a variety of reasons, including cultural norms and community perceptions. In Afar cultures, discussions of SRH issues are taboo or stigmatized, which can make it difficult for young people to access information or services. These negative perceptions of SRH services or that the community views that they are inappropriate, have created barriers to accessing services. These cultural and community factors can make it difficult for adolescents to access the SRH services they need, which can negatively impact their health and well-being. A HCP pointed out:

> "When a school girl from the camp comes to the SRH clinic, people think, 'Oh, this girl has already started having sex,' and so young people are ashamed to come and ask; they prefer to solve these problems themselves". KII age 20-30 years

Insensitivity to cultural practices

Ethiopian health workers in the camp were generally positive about the Eritrean refugee women they care for, emphasizing that they strive to provide the same level of care to all women in need. In the Asayta refugee camp, where women come from different cultural backgrounds, they expect these cultural practices to be respected in all circumstances, but the structure of the health system makes this extremely difficult.

> "This (sensitivity to cultural practices) is especially important for HCP, who must be sensitive to the cultural practices and beliefs of the refugee women they serve to provide respectful and appropriate care. Majority of HCP are strangers usually have difficulties in responding to the cultural needs of Eritrean refugee women, which can lead to misunderstanding or miscommunication and make it difficult for patients to

For peer review only - http://bmjopen.bmj.com/site/about/guidelines.xhtml

receive the care they need. In some cases, the cultural insensitivity of health professionals may also contribute to a lack of trust in the healthcare system, which can further impede access to care." KII age 30-40 years.

Lack of husband involvement in SRH services

The lack of involvement of husbands in SRH services is a significant barrier to the uptake of these services, particularly in areas such as Afar where SRH is seen as a woman's issue or where there are cultural or social norms that discourage men from becoming involved in these issues. Men's lack of participation can negatively impact men's and women's health and well-being by limiting access to information and services that are important for preventing unintended pregnancies and STI.

> "There are several sociocultural barriers such as restrictive norms and stigma around adolescent and young adult sexuality, harmful gender norms, and discrimination by communities, families, partners, and providers. Men have low knowledge about SRH issues. As a result, they do not support their wives to access SRH services. In general, the culture does not support the use of some SRH services, particularly family planning services. As a result, the community does not have positive attitudes toward family planning, and men and elders do not want their wives and children to limit the number of offspring they bear." KII age 20-30 years.

For peer review only

Gender-Based Violence (GBV)

In a humanitarian setting, violence against women is more common with limited access to support systems and basic services. Common forms of violence against women in the Asayta refugee camp include physical, sexual, and emotional abuse, as well as financial exploitation and female genital mutilation (FGM). These types of violence are perpetrated by individuals, such as intimate partners or family members, or by groups or institutions. Violence against women is normalized and occurs more frequently because of conservative cultural, social, and religious norms that condone violence against women and girls as normal, acceptable behavior. A HCP stated:

> "As a concerned professional, I know that violence against women is prevalent in our community and is not effectively addressed due to cultural, social, and religious norms. Violence against women and girls is a normal practice here and is never condemned by the community. Moreover, violence by husbands against their wives tends to be justified. Therefore, it is more difficult to recognize and respond to, as it may be considered a normal or acceptable part of daily life. This can make it more difficult to prevent GBV and provide support and services to those who have experienced it. It is important that communities and societies actively address and work to prevent the normalization of violence against women to create a safer and more equitable environment for all. This can include education and awareness-raising efforts, but also trying to change harmful cultural norms and practices that contribute to GBV." KII age 30-40 years.

Policy level factors

This fifth level of the SEM framework adds a macro perspective that considers the broader context. The national, local, and humanitarian policies and laws that regulate or support the SRH needs of refugee women are considered.

The "Out of Camp" policy introduced by the Ethiopian government in 2010 aims to promote the integration of Eritrean refugees into the host society. This includes access to education for refugee students at all levels, including primary, secondary, and higher education institutions. The Ethiopian government has taken steps to provide free SRH services in all public health facilities. This is an effective way to encourage women to use these services and improve SRH. However, HCP pointed out that it is also important to address other confounding barriers that prevent women from accessing these services, such as the cost of transportation and housing.

> "*There* are still indirect costs that can place a significant burden on pastoral women, especially those living in humanitarian crises, such as refugees. It might be helpful to think about implementing additional policies or programs to address these types of barriers, such as providing transportation vouchers or subsidies for housing costs. This could help ensure that all people have equal access to the health services they need." KII age 30-40 years.

For peer review only - http://bmjopen.bmj.com/site/about/guidelines.xhtml

Weak coordination and collaboration

Lack of coordination and collaboration between different actors of RRS and the Ethiopian Ministry of Health structure and beyond led to inefficiencies and gaps in care. Coordination between the different actors responsible for the refugees is cited as a major challenge in implementing an integrated health system, especially in emergencies.

> “Poor coordination among stakeholders has serious consequences. It can lead to duplication, confusion, and misunderstanding, which can ultimately be detrimental to patient health and well-being. It may be helpful to establish clear protocols for better communication and collaboration. This could include regular meetings or a centralized coordination system for sharing information and resources.” KII age 30-40 years.

Training needs of HCP at the refugee clinic

HCP in the Asayta refugee clinic need specialized training including psychological training to meet the changing health needs of refugee women. They recognized that the SRH needs of refugee women are "more complex" and "completely different," requiring intensive preparation in culturally sensitive skills. Despite this realization, most had not participated in any training that specifically addressed refugee women's health care.

> “Almost all refugee women have experienced trauma and all demand special needs. HCP need to be trained to recognize these needs and address them in a sensitive and culturally appropriate way. This is a very specific topic area (intercultural competence) that is not even addressed in universities.” KII age 20-30 years.

Health system resilience

Another very important issue that needs to be addressed at the macro level is the resilience of health systems in the region, including in refugee camps, which are not well prepared for natural and man-made emergencies and shocks. As a result, populations lack access to basic health services during times of war, pandemics, and natural disasters. The existing health system was easily overwhelmed during the war in northern Ethiopia, the COVID-19 pandemic, and the locust invasion, leaving the population without access to essential health services.

> “The security situation in the region and even in Ethiopia is volatile; peaceful occasions can rapidly turn into conflicts and devastating situations, as we experienced during the war in northern Ethiopia, when large numbers of people were displaced from northern Afar and Tigray to the town of Asayta, overwhelming the existing health system. At a certain stage of the peace agreement, these IDP are forced to return to their original place of residence. In such cases, the health system is unable to meet the demands of the community, and resilience is severely compromised. Resilience-building strategies should be implemented, including strengthening emergency preparedness and response plans, increasing investment in the health system, and developing a more robust disaster forecasting and analysis system. In addition, partnerships with international organizations and other actors may be beneficial to provide additional

For peer review only - http://bmjopen.bmj.com/site/about/guidelines.xhtml

resources and expertise to improve the health system in the region." KII age 30-40 years.

Discussion

Refugee women encounter significant inequalities in accessing and uptake of SRH services despite substantial influx of migrants towards developing countries. Eritrean refugee women experienced worse health outcomes than the host population. This study identified numerous barriers hindering refugee women's access to appropriate SRH services. Analyzed through a Socio-Ecological Model (SEM) framework, these challenges spanned five levels: individual, social, community, institutional, and policy. A similar multi-level intervention approach is suggested by Vlahov et al. to address the needs of women in humanitarian settings [58]. This reinforces the notion that providing effective SRH services requires collaboration and coordination across various complex systems, as highlighted by studies conducted in Bangladesh and Australia [7, 35].

Individual-level SEM factors such as migration history, education, SRH knowledge, age, culture, socio-economic background, experience of health care, and length of stay in the camp were found to shape women's perceptions of their health and well-being, as well as their access to and utilization of SRH services. These results are in line with studies conducted [39, 59] and Multicultural Centre for Women's Health [30], which showed poor knowledge about SRH. Interactions with people, and peer pressure, which can provide social support or create barriers might affect a woman's healthy behavior perceptions. Peers, partners, and clan members influence SRH service use behaviors. Interaction between HCP at the same time influences health service uptake [60, 61]. Language and cultural expectations were prevalent predominant themes that emerged as common SRH barriers among refugee and host community women in Asayta. Differences in language expectations were reported to affect SRH care for both HCP and women. Similar study findings highlight that different expectations between HCP and women are significantly affecting service quality [36, 37, 61-63]. High staff turnover, lack of essential services, laboratory, pharmacy, long waiting times, frequent referral, and transport problems were the most common barriers reported at the health facility level by study participants. Similar findings are reported from refugee camps in Kenya [64, 65].

Even though GBV is a widespread problem affecting people around the world, and it is particularly prevalent in conflict and displacement situations such as refugee camps and temporary settlements, it is concerning to witness the normalization of GBV. During migration and human trafficking, there are horrific stories of violence and yet are still taken as normal in the system. This finding is supported by a study conducted in Nigeria and many other countries where community acceptance of this abusive relationship is more entrenched and where women suffer in silence as they are often protected by family secrecy, cultural norms, fear, shame, and associated social stigma and therefore remain silent [66-70]. This is mainly due to the general religious teachings that women must submit and obey their partner as head of the family [69, 71]. SRH topics in the camp are also taboo topics similar to other studies[72]. On a similar token HCP in Asayta generally do not perform safe abortions due to prevailing negative perceptions. Women who need abortions often turn to private health facilities. The same findings are reported in many African countries where SRH is largely a

For peer review only - http://bmjopen.bmj.com/site/about/guidelines.xhtml

taboo topic, intentionally avoided or not addressed either by the HCP or the healthcare seeker because it is socio-culturally unacceptable or connected with strong feelings of shame [6, 73, 74]. A macro perspective model that considers the broader context focus was on national, local, and humanitarian policies and laws that regulate or support the SRH needs of Eritrean refugee women in the Asayta camp. These included policies on community integration, livelihood, and access to services such as healthcare, education, housing, epidemics control, and disease management. In agreement with other studies [39, 75], HCP in this study indicated that factors at all levels of SEM influence the SRH needs of Eritrean refugee women. HCP perceived that Eritrean refugee women SRH needs and engagement with care varied across SEM perspectives.

The SRH needs of refugees and host communities have many similarities. The mentioned difference in a health seeking difference could be due to the mandatory linkage of humanitarian aid services with the use of maternal health services in the camp clinic. This can be an effective strategy to promote SRH seeking behavior, but it is also important to ensure that these services are culturally appropriate and meet the needs of the population they are intended to serve.

Under the available laws, refugees in Ethiopia are entitled to protection from persecution and to the same rights and privileges as Ethiopian citizens, with some exceptions. These include the right to education, the right to work and engage in economic activity, and the right to freedom of movement within certain limits [76, 77]. HCP reiterated the importance of addressing indirect cost barriers that can prevent women from accessing these services, such as transportation and housing costs. Although Eritrean refugees have a right to access health services, including SRH care, they face multiple complexities where access to services is limited by several factors, including geographic remoteness, lack of resources, and socio-cultural barriers.

The lack of alignment between the national health system and the RRS system of refugee creates barriers for HCP in those camps and they face challenges in accessing continuous professional development (CPD) opportunities. As a result, most camp providers lack access to the latest advancements in disease management, treatment protocols, and other essential areas, potentially compromising the quality of care they can deliver.

Another crucial issue is the resilience of the health system in the refugee setting. This situation is not well prepared for emergencies and shocks such as disease outbreaks, natural disasters, or conflict. The existing health system was easily overwhelmed by triple disasters (war in northern Ethiopia, COVID-19 pandemic, and locust invasion), leaving the population without access to basic health services. Eritrean refugees were among the most vulnerable populations affected by the grave civilian atrocities, displacement, and deteriorating humanitarian situation in northern Ethiopia [78]. Thus, effective refugee policies that are designed to protect the rights and well-being of refugees, while also taking into account the needs and concerns of host communities are needed more than ever. This also highlights the need for increased support from the international community to help Ethiopia meet the needs of refugees and improve the resilience of its health system.

For peer review only - http://bmjopen.bmj.com/site/about/guidelines.xhtml

Strength and limitations

The socioecological model enabled this study to comprehensively explore the multi-level factors influencing refugee women's SRH needs, from individual experiences to societal structures, revealing insights not captured by previous studies in the country. This study is the first attempt to examine SRH service utilization from the perspective of key informant researcher approach. This again provides a unique and insightful starting point for understanding the factors that influence SRH service utilization among refugee women in a humanitarian crisis settings. Exploring SRH studies presents unique challenges due to the sensitive nature of the topic. Participants may hold biases or fears regarding the researchers' intentions, potentially introducing bias into the study. Additionally, building trust and rapport with participants can be difficult, impacting the study's success. HCP were recruited from the Asayta district; therefore, comparing results with other sites may be difficult because different policies and programs apply in different refugee camps in different regions. To promote reflexivity and mitigate these biases, triangulation of evidences through focused observation was done.

Implications for Research, Policy, and Practice

This qualitative study provides invaluable insight into the SRH of Eritrean refugee women from the perspectives of HCP. The study findings confirmed that SRH topics are taboos and act as a barrier to access SRH services. Likewise, it should be ensured that HCP working in the area of SRH should be equipped with the proper training on cultural, compassionate, and respectful competency care approaches. SRH consultations should take place in a safe and supportive manner. Healthcare facilities in the camp should also create private spaces for consultations to maintain the confidentiality of SRH services. Further research is needed to explore the knowledge and attitudes of HCP in SRH care approaches, and in understanding the barriers and facilitators towards providing comprehensive SRH service. This has the potential to facilitate the proper use of SRH services consequently leading to overall improvements to women health.

Even though SRH is included in Ethiopian social policy, the implementation of rules and regulation are not available or very minimal. It is critical to establish national policies and guidelines describing the standard of care in the area of SRH and clarify providers' roles in offering SRH advice. The findings may also be useful for public health implementers and policy evaluators, as they could broaden their understanding of what works more effectively and for whom, in fragile humanitarian contexts, with the aim of improving SRH service use among refugee women.

Conclusions

Humanitarian care settings are often characterized by the breakdown of basic health services. This study highlights the higher unmet SRH needs of Eritrean refugee women in Ethiopia, with severely limited service availability. The findings showed the need to develop interventions that simultaneously target multiple levels of SEM highlighting the importance of evidence-

For peer review only - http://bmjopen.bmj.com/site/about/guidelines.xhtml

For peer review only

based solutions at the individual, group, community, institutional, and policy levels to address the SRH needs of Eritrean refugee women. For example, at the individual level, women's past experiences can pose challenges to SRH service use. At the interpersonal level, lack of cultural competence, communication difficulties, cultural differences, misunderstandings, and misconceptions were found to impact service delivery. The results have the potential to have an even greater impact, as lessons learned from SRH decision-making can be applied to other general health services. Implementation of lessons learned about refugee health in Ethiopia should also take into account differences in culture and the traditional needs of the target populations. Underlying all solutions and their implementation should be the empowerment of women to optimize their knowledge of SRH.

Given the COVID-19 pandemic and fragile situation, health needs could be challenged at any time, preparation is crucial at all levels of the SEM to ensure health system resilience. Future public health interventions must build on stakeholder input and address all aspects of SEM to achieve the SDG and reduce maternal mortality. This could involve advocating for increased resources and funding, establishing partnerships for collaboration, and distributing supplies.

The need for better collaboration between the RRS health system and the regional (district) health system is very paramount. Developing a comprehensive and practical program, as well as legislative and policy support for this issue, can provide the foundation for SRH care for this group of women in refugee camps. Training HCP from the local area who are familiar with culture and language can provide the services effectively. The lessons learned through HCP are important for both policy and practice, as they provide a framework for planning, developing, and implementing interventions for governments and partners charged with improving the use of SRH services. It is also important to work with community leaders and other influential figures to promote gender equality and address harmful cultural norms.

For peer review only

Authors' contributions

NBZ: conceptualisation, methodology, investigation, analysis, and writing original draft. SM, AAM, DHP, and NPH provided critical review and supervision. CA, AC and RT contributed for project development and coordination. All authors have read, and approved the final version.

Funding

The "HEALTH NIIDS"project supported this work through the generous financial support of the Stanley Thomas Johnson Foundation and the State Secretariat for Education, Research and Innovation (SERI), Switzerland with grant number 1053-KF. The funders have no role in the design, analysis, and dissemination of the findings.

Ethics statements

Patient consent for publication

Not applicable.

Competing interests

The authors declare that they have no competing interests

Availability of data and materials

All data collected were qualitative in nature. The data relevant to the study are included in the article or uploaded as supplementary information.

Ethics approval

Ethical approval was obtained from the Ethics Committee of Northwestern and Central Switzerland (EKNZ; EKNZ No. 2020-02154) in Basel, Switzerland, and from the Research Ethics Committee of the Armauer-Hansen Research Institute (AHRI) in Ethiopia as part of the "HEALTH- NIIDS" project. Permission was also obtained from the Refugees and Returnees Service (RRS) in Addis Ababa, Ethiopia. The purpose, objectives, and expectations of the study were explained to the study participants. The information sheet was read to participants in the local language so that they could understand their rights. Unique identification numbers and codes were used to maintain confidentiality. KII were conducted individually and in private. All participant related individual files and participants' identities were encrypted and kept confidential via password-protected computer access to maintain the anonymity of all data. Participation in the study was completely voluntary and written informed consent was obtained from all study subjects.

Acknowledgments

We are very grateful to all study participants for their time and valuable contributions. Our appreciation goes to the entire NIIDS project team in Switzerland and Ethiopia. We are also grateful to Karen Maigetter for her invaluable assistance with the language edits.

List of figures

Figure 1. A socio-ecological analysis framework of factors that influence HCPs' readiness to provide SRH services to Eritrean refugee women in Asayta refugee camp, Ethiopia (adapted).

References

1. WHO, *Report on the health of refugees and migrants in the WHO European Region: no public health without refugee and migrant health (2018)* 2018, WHO: Denmark.
2. Organization., W.H., *Adolescent pregnancy: evidence brief*. 2019, World Health Organization.
3. Organization, W.H., *Universal health coverage for sexual and reproductive health: evidence brief*. 2020, World Health Organization.
4. Kurth, E., et al., *Reproductive health care for asylum-seeking women - a challenge for health professionals.* BMC Public Health, 2010. **10**: p. 659.
5. Jannat, S., R.I. Sifat, and M. Khisa, *Sexual and Reproductive Health Conditions of Women: Insights from Rohingya Refugee Women in Bangladesh.* Sexuality Research and Social Policy, 2023. **20**(3): p. 855-868.
6. Kennedy, E.C., et al., *"These issues aren't talked about at home": a qualitative study of the sexual and reproductive health information preferences of adolescents in Vanuatu.* BMC Public Health, 2014. **14**(1): p. 770.

For peer review only - http://bmjopen.bmj.com/site/about/guidelines.xhtml

7. Ahmed, R., et al., *Situation analysis for delivering integrated comprehensive sexual and reproductive health services in humanitarian crisis condition for Rohingya refugees in Cox's Bazar, Bangladesh: protocol for a mixed-method study.* BMJ Open, 2019. **9**(7): p. e028340.
8. UNHCR. *Global Trends*. 10th January 2023]; Available from: https://www.unhcr.org/globaltrends
9. UNHCR. *UNHCR Ethiopia Refugee Statistics as of June 2023* 2023 [cited 2023 17.07.2023]; Available from: https://data2.unhcr.org/en/documents/download/101981.
10. Getachew, M., et al., *Magnitude and factors associated with adherence to Iron-folic acid supplementation among pregnant women in Eritrean refugee camps, northern Ethiopia.* BMC Pregnancy Childbirth, 2018. **18**(1): p. 83.
11. UNHCR. *Sharp increase in number of Eritrean refugees and asylum-seekers in Europe, Ethiopia and Sudan*. [cited 2023 10th january]; Available from: https://www.unhcr.org/news/briefing/2014/11/5465fea1381/sharp-increase-number-eritrean-refugees-asylum-seekers-europe-ethiopia.html.
12. International Organization for Migration (IOM), *International Organization for Migration (IOM), Aug 05 2022. DTM Ethiopia — National Displacement Report 12 (March— April 2022). IOM, Ethiopia.* . 2022: Switzerland.
13. VOA, *Report: Eritrean Refugees Relocated from Addis Ababa to Dangerous Area* 2022, VOA.
14. UNHCR. *Ethiopia's Tigray Refugee Crisis Explained*. 2022 [cited 2022 13.12.2022]; Available from: https://www.unrefugees.org/news/ethiopia-s-tigray-refugee-crisis-explained/.
15. UNHCR. *Ethiopia's Tigray Refugee Crisis Explained*. 2022 [cited 2023 30.04]; Available from: https://www.unrefugees.org/news/ethiopia-s-tigray-refugee-crisis-explained/.
16. UNHCR, *Inter-agency Field Manual on Reproductive Health in Humanitarian Settings*. 2010.
17. Gürol, E.C.T.a.S., *The effects of migration on women's health and reproductive health in Turkey.* Frontiers in Women's Health, 2019. **4**.
18. United Nations, D.o.E.a.S.A., Population Division, *International Migrant Stock 2019. (United Nations database, POP/DB/MIG/Stock/Rev.2019).* 2019, IOM: Berlin.
19. Gebrecherkos, K., et al., *Unmet need for modern contraception and associated factors among reproductive age group women in Eritrean refugee camps, Tigray, north Ethiopia: a cross-sectional study.* BMC Res Notes, 2018. **11**(1): p. 851.
20. Ahmed, J.A., et al., *Epidemiology of respiratory viral infections in two long-term refugee camps in Kenya, 2007-2010.* BMC Infectious Diseases, 2012. **12**(1): p. 7.
21. Organization, W.H., *WHO recommendations on intrapartum care for a positive childbirth experience*. 2018: World Health Organization.
22. Darebo, T.D., et al., *The sexual and reproductive healthcare challenges when dealing with female migrants and refugees in low and middle-income countries (a qualitative evidence synthesis).* BMC Public Health, 2024. **24**(1): p. 520.
23. Cignacco, E., et al., *Sexual and reproductive healthcare for women asylum seekers in Switzerland: a multi-method evaluation.* BMC Health Serv Res, 2018. **18**(1): p. 712.
24. IDMC, *IDMC's 2019 Global Report on Internal Displacement*, NRC, Editor. 2019.
25. Chernet, A., *Cardiovascular diseases risk factors among recently arrived Eritrean refugees in Switzerland.* BMC Res Notes, 2019. **12**.
26. Economic, U.N.D.f., S. Information, and P. Analysis, *Population and Development: Programme of Action Adopted at the International Conference on Population and Development, Cairo, 5-13 September 1994*. Vol. 1. 1995: United Nations Publications.
27. UNFPA, *Adolescent Pregnancy: a review of evidence* 2013.
28. Fincher, R.A., *International conference on population and development.* Envtl. Pol'y & L., 1994. **24**: p. 309.
29. Austin, J., et al., *Reproductive health: a right for refugees and internally displaced persons.* Reproductive health matters, 2008. **16**(31): p. 10-21.

For peer review only - http://bmjopen.bmj.com/site/about/guidelines.xhtml

30. Health;, M.C.f.W.s., *Act now to advance health equity for migrant and refugee women's sexual and reproductive health*. 2021.
31. Usta, J., J.A.M. Farver, and L. Zein, *Women, war, and violence: surviving the experience.* Journal of Women's Health, 2008. **17**(5): p. 793-804.
32. Barnes, D.M. and C.L. Harrison, *Refugee women's reproductive health in early resettlement.* Journal of Obstetric, Gynecologic, & Neonatal Nursing, 2004. **33**(6): p. 723-728.
33. Bronfenbrenner, U., *The ecology of human development: Experiments by nature and design*. 1979: Harvard university press.
34. Kilanowski, J.F., *Breadth of the socio-ecological model.* Journal of Agromedicine, 2017. **22**(4): p. 295-297.
35. Mengesha, Z.B., et al., *Preparedness of Health Care Professionals for Delivering Sexual and Reproductive Health Care to Refugee and Migrant Women: A Mixed Methods Study.* Int J Environ Res Public Health, 2018. **15**(1).
36. Newbold, K.B. and J. Willinsky, *Providing family planning and reproductive healthcare to Canadian immigrants: perceptions of healthcare providers.* Culture, health & sexuality, 2009. **11**(4): p. 369-382.
37. Ng, C. and K.B. Newbold, *Health care providers' perspectives on the provision of prenatal care to immigrants.* Cult Health Sex, 2011. **13**(5): p. 561-74.
38. King, R., et al., *Barriers and facilitators to accessing skilled birth attendants in Afar region, Ethiopia.* Midwifery, 2015. **31**(5): p. 540-6.
39. Mengesha, Z.B., et al., *Refugee and migrant women's engagement with sexual and reproductive health care in Australia: A socio-ecological analysis of health care professional perspectives.* PLoS One, 2017. **12**(7): p. e0181421.
40. Lazar, J.N., et al., *Providers' Perceptions of Challenges in Obstetrical Care for Somali Women.* Obstetrics and Gynecology International, 2013. **2013**: p. 149640.
41. Fentaw, R., A. Bogale, and D. Abebaw, *Prevalence of child malnutrition in agro-pastoral households in Afar Regional State of Ethiopia.* Nutr Res Pract, 2013. **7**(2): p. 122-31.
42. Dykes, F. and R. Flacking, *Ethnographic research in maternal and child health*. 2016: Routledge Milton Park, Abingdon, Oxon.
43. McLeroy, K.R., et al., *An ecological perspective on health promotion programs.* Health education quarterly, 1988. **15**(4): p. 351-377.
44. Vu, M., et al., *System-level factors influencing refugee women's access and utilization of sexual and reproductive health services: A qualitative study of providers' perspectives.* Frontiers in Global Women's Health, 2022. **3**: p. 1048700.
45. Sawadogo, P.M., et al., *Barriers and facilitators of access to sexual and reproductive health services among migrant, internally displaced, asylum seeking and refugee women: A scoping review.* Plos one, 2023. **18**(9): p. e0291486.
46. Davidson, N., et al., *Access to preventive sexual and reproductive health care for women from refugee-like backgrounds: a systematic review.* BMC Public Health, 2022. **22**(1): p. 403.
47. Tirado, V., et al., *Barriers and facilitators for the sexual and reproductive health and rights of young people in refugee contexts globally: A scoping review.* PloS one, 2020. **15**(7): p. e0236316.
48. Shtarkshall, R.A., F. Baynesan, and B.S. Feldman, *A socio-ecological analysis of Ethiopian immigrants' interactions with the Israeli healthcare system and its policy and service implications.* Ethnicity & health, 2009. **14**(5): p. 459-478.
49. Keygnaert, I., N. Vettenburg, and M. Temmerman, *Hidden violence is silent rape: sexual and gender-based violence in refugees, asylum seekers and undocumented migrants in Belgium and the Netherlands.* Culture, health & sexuality, 2012. **14**(5): p. 505-520.
50. Wells, R., et al., *An ecological model of adaptation to displacement: individual, cultural and community factors affecting psychosocial adjustment among Syrian refugees in Jordan.* Global Mental Health, 2018. **5**.

For peer review only

For peer review only - http://bmjopen.bmj.com/site/about/guidelines.xhtml

51. Guruge, S. and N. Khanlou, *Intersectionalities of influence: researching the health of immigrant and refugee women.* Canadian Journal of Nursing Research Archive, 2004: p. 32-47.
52. White, K., M. Ocampo, and I.C. Scarinci, *A socio-ecological approach for examining factors related to contraceptive use among recent Latina immigrants in an emerging Latino state.* Women & Health, 2017. **57**(7): p. 872-889.
53. Braun, V. and V. Clarke, *Using thematic analysis in psychology.* Qualitative Research in Psychology, 2006. **3**(2): p. 77-101.
54. Gale, N.K., et al., *Using the framework method for the analysis of qualitative data in multi-disciplinary health research.* BMC medical research methodology, 2013. **13**(1): p. 1-8.
55. Finlay, L. and B. Gough, *Reflexivity: A practical guide for researchers in health and social sciences*. 2008: John Wiley & Sons.
56. Tong, A., P. Sainsbury, and J. Craig, *Consolidated criteria for reporting qualitative research (COREQ): a 32-item checklist for interviews and focus groups.* International journal for quality in health care, 2007. **19**(6): p. 349-357.
57. O'Brien, B.C., et al., *Standards for reporting qualitative research: a synthesis of recommendations.* Acad Med, 2014. **89**(9): p. 1245-51.
58. Vlahov, D., et al., *Strategies for improving influenza immunization rates among hard-to-reach populations.* Journal of Urban Health, 2007. **84**: p. 615-631.
59. Korri, R., G. Froeschl, and O. Ivanova, *A Cross-Sectional Quantitative Study on Sexual and Reproductive Health Knowledge and Access to Services of Arab and Kurdish Syrian Refugee Young Women Living in an Urban Setting in Lebanon.* Int J Environ Res Public Health, 2021. **18**(18).
60. Control, C.f.D. and Prevention, *The social-ecological model: A framework for prevention*. 2015.
61. Mengesha, Z.B., et al., *Challenges in the Provision of Sexual and Reproductive Health Care to Refugee and Migrant Women: A Q Methodological Study of Health Professional Perspectives.* Journal of Immigrant and Minority Health, 2018. **20**(2): p. 307-316.
62. Essén, B., et al., *Are some perinatal deaths in immigrant groups linked to suboptimal perinatal care services?* BJOG: An International Journal of Obstetrics and Gynaecology, 2002. **109**(6): p. 677-682.
63. Pottie, K., et al., *Language proficiency, gender and self-reported health.* Canadian Journal of Public Health, 2008. **99**(6): p. 505-510.
64. Horn, R., *Responses to intimate partner violence in Kakuma refugee camp: refugee interactions with agency systems.* Social science & medicine, 2010. **70**(1): p. 160-168.
65. Wright, L.-A. and R. Plasterer, *Beyond basic education: Exploring opportunities for higher learning in Kenyan refugee camps.* Refuge, 2010. **27**: p. 42.
66. Women, U., *The World for Women and Girls Annual Report 2019-2020.* 2020.
67. OBAGBOYE, T.G., *ENTRENCHED EPIDEMIC: STEMMING THE TIDE OF VIOLENCE AGAINST WOMEN IN NIGERIA AND AFRICA.* INTERNATIONAL JOURNAL OF COMPARATIVE LAW AND LEGAL PHILOSOPHY (IJOCLLEP), 2020. **2**(1).
68. Muluneh, M.D., et al., *Gender based violence against women in sub-Saharan Africa: a systematic review and meta-analysis of cross-sectional studies.* International journal of environmental research and public health, 2020. **17**(3): p. 903.
69. Barrett, B.J. and M.S. Pierre, *Variations in women's help seeking in response to intimate partner violence: Findings from a Canadian population-based study.* Violence against women, 2011. **17**(1): p. 47-70.
70. Organization, W.H., *Responding to intimate partner violence and sexual violence against women: WHO clinical and policy guidelines*. 2013: World Health Organization.

For peer review only

For peer review only - http://bmjopen.bmj.com/site/about/guidelines.xhtml

71. Chawhanda, C., J. Levin, and L. Ibisomi, *Factors associated with sexual and reproductive health service utilisation in high migration communities in six Southern African countries.* BMC Public Health, 2022. **22**(1): p. 876.
72. Grandey, A.A., A.S. Gabriel, and E.B. King, *Tackling taboo topics: A review of the three M s in working women's lives.* Journal of Management, 2020. **46**(1): p. 7-35.
73. Traumer, L., M.H. Jacobsen, and B.S. Laursen, *Patients' experiences of sexuality as a taboo subject in the Danish healthcare system: a qualitative interview study.* Scandinavian journal of caring sciences, 2019. **33**(1): p. 57-66.
74. Steven, V.J., et al., *"Provide care for everyone please": engaging community leaders as sexual and reproductive health advocates in North and South Kivu, Democratic Republic of the Congo.* Reproductive Health, 2019. **16**(1): p. 98.
75. Akhavan, S. and S. Karlsen, *Practitioner and client explanations for disparities in health care use between migrant and non-migrant groups in Sweden: a qualitative study.* Journal of Immigrant and Minority Health, 2013. **15**(1): p. 188-197.
76. Canton, H., *United Nations High Commissioner for Refugees—UNHCR*, in *The Europa Directory of International Organizations 2021*. 2021, Routledge. p. 215-234.
77. Seyife, A., et al., *Utilization of modern contraceptives and predictors among women in Shimelba refugee camp, Northern Ethiopia.* PloS one, 2019. **14**(3): p. e0212262.
78. UNHCR, *Deteriorating conditions putting Eritrean refugees at grave risk in Tigray*. 2022, UNHCR: Switzerland

For peer review only

Figure 1

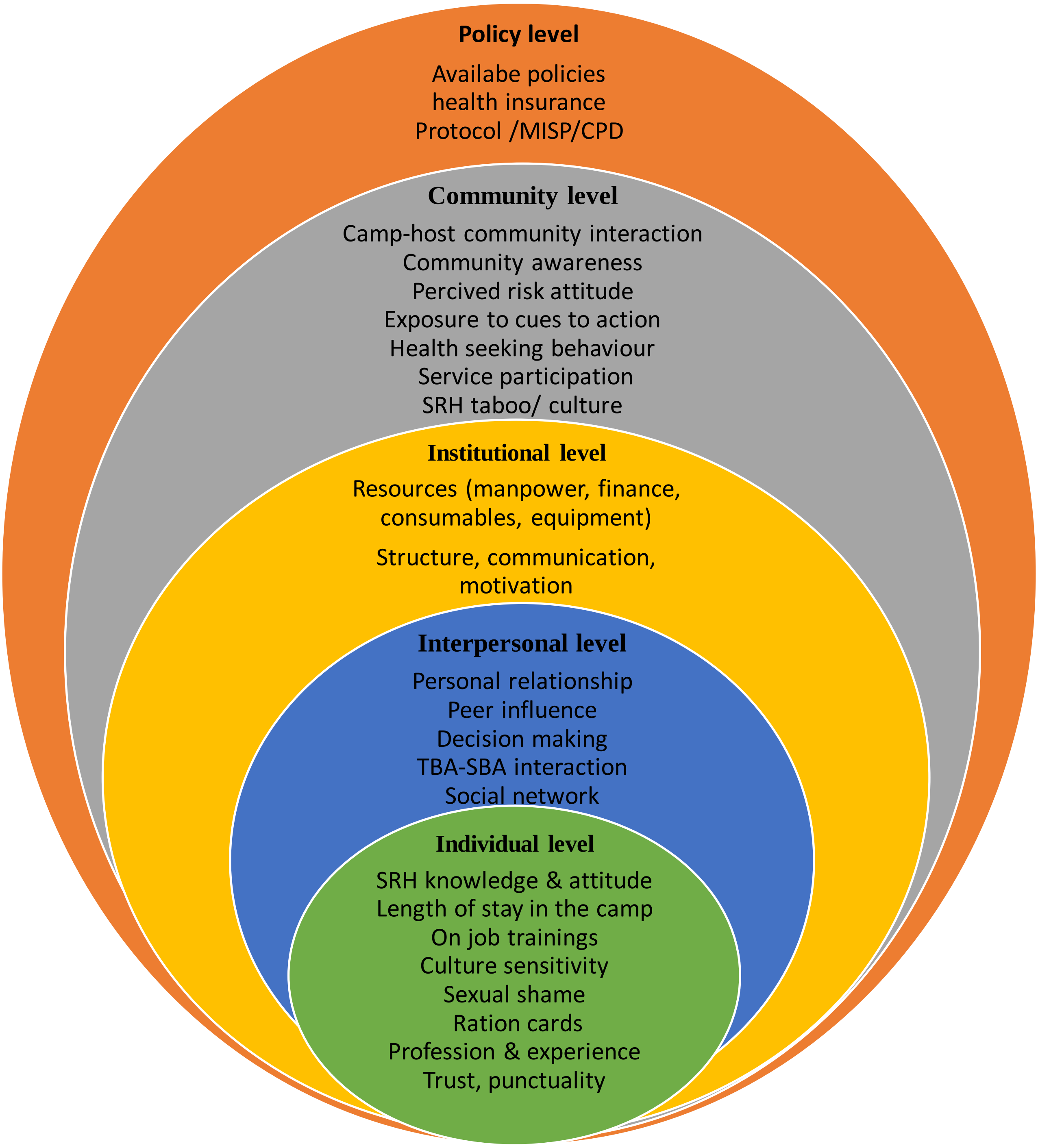

Figure 1. A socio-ecological analysis framework of factors that influence HCPs' readiness to provide SRH services to Eritrean refugee women in Asayta refugee camp, Ethiopia (adapted).

Additional file 3

Standards for Reporting Qualitative Research (SRQR)*

http://www.equator-network.org/reporting-guidelines/srqr/

**Title and abstract**	**Page/line no**
**Title** - Concise description of the nature and topic of the study Identifying the study as qualitative or indicating the approach (e.g., ethnography, grounded theory) or data collection methods (e.g., interview, focus group) is recommended	Page# 1, line #1-2
**Abstract** - Summary of key elements of the study using the abstract format of the intended publication; typically includes background, purpose, methods, results, and conclusions	Page# 2, line #32-53

Introduction

**Problem formulation** - Description and significance of the problem/phenomenon studied; review of relevant theory and empirical work; problem statement	Page# 3-5, line #71-151
**Purpose or research question** - Purpose of the study and specific objectives or questions	Page# 4-5, line #151-172

Methods

**Qualitative approach and research paradigm** - Qualitative approach (e.g., ethnography, grounded theory, case study, phenomenology, narrative research) and guiding theory if appropriate; identifying the research paradigm (e.g., postpositivist, constructivist/ interpretivist) is also recommended; rationale**	Page# 5, line #196-199 And Page# 7-8, line #251-299
**Researcher characteristics and reflexivity** - Researchers' characteristics that may influence the research, including personal attributes, qualifications/experience, relationship with participants, assumptions, and/or presuppositions; potential or actual interaction between researchers' characteristics and the research questions, approach, methods, results, and/or transferability	Page# 5, line #221-237 And #299-325
**Context** - Setting/site and salient contextual factors; rationale**	Page# 5, line #175-194
**Sampling strategy** - How and why research participants, documents, or events were selected; criteria for deciding when no further sampling was necessary (e.g., sampling saturation); rationale**	Page# 6, line #202-207 and #214-248

For peer review only

**Ethical issues pertaining to human subjects** - Documentation of approval by an appropriate ethics review board and participant consent, or explanation for lack thereof; other confidentiality and data security issues	Page# 24, line #891-902
**Data collection methods** - Types of data collected; details of data collection procedures including (as appropriate) start and stop dates of data collection and analysis, iterative process, triangulation of sources/methods, and modification of procedures in response to evolving study findings; rationale**	Page# 6, line #213-248

Results/findings

**Synthesis and interpretation** - Main findings (e.g., interpretations, inferences, and themes); might include development of a theory or model, or integration with prior research or theory	Page# 9-19, line #328-721
**Links to empirical data** - Evidence (e.g., quotes, field notes, text excerpts, photographs) to substantiate analytic findings	

Discussion

**Integration with prior work, implications, transferability, and contribution(s) to the field** - Short summary of main findings; explanation of how findings and conclusions connect to, support, elaborate on, or challenge conclusions of earlier scholarship; discussion of scope of application/generalizability; identification of unique contribution(s) to scholarship in a discipline or field	Page# 20-, line #722-805
**Limitations** - Trustworthiness and limitations of findings	Page# 22, line#808-821

Other

**Conflicts of interest** - Potential sources of influence or perceived influence on study conduct and conclusions; how these were managed	Page# 23, line #886
**Funding** - Sources of funding and other support; role of funders in data collection, interpretation, and reporting	Page# 23, line #878-881

For peer review only

KII with health professionals and Key officers

Health professionals in the refugee camp/CH: health professionals treating Eritrean/Somali migrants

Demographic Characteristics of the study participants
Place
Date
Interviewers

ID	Age	Type of the professional*	Experience in camp/center	Religion	Education level	Nationality

***Medical doctor, Nurse, Public health etc.**

For peer review only

1. Survey of the present health status:

- Is a general clinical assessment conducted for women arriving at the camp/asylum centre? If yes, please indicate which kind (physical/psychological/gynaecological)?Where is the clinical assessment usually conducted? Who is usually present during the examination? Is the clinical examination done by a man or a woman? How many persons are present during a gynecological examination?
- Is an interpreter consulted during assessment? If not, why not?
- Is a professional interpreter available?
- How are the confidentiality and privacy maintained when SRH issues are discussed

2. Psychological healthcare:

- Is data on experiences with violence / trauma systematically collected?
- Is an interpreter consulted during this procedure? If not, why not?
- What is the course of action if a current traumatisation is found?
- What should be improved according to you?

For peer review only - http://bmjopen.bmj.com/site/about/guidelines.xhtml

3. Gynaecological healthcare:

- How can an asylum-seeking woman get information on contraception and family planning that she can understand (considering language, literacy) ? How does she get information on consultations?
- Who can a woman go to if she has acute or chronic gynaecological problems (e.g. vaginal infections, bladder infections, menstrual problems, strong or foul-smelling discharge)? How well are women informed about these possibilities? Are they accessing these services, and who supports them in this? Options for improvement?
- Where are prophylactic gynaecological examinations conducted?
- Is an interpreter consulted during the examination? Who is present during the examination?

4. Healthcare during pregnancy:

- How are the pregnancy check-ups organised?
- Is the woman familiarised with different models of healthcare during pregnancy? (e.g. freelance midwife or gynaecologist, hospital care)? Are pregnant women given a choice? How can they get support (e.g. in terms of accompanying, interpreting) if they want to access ANC services?
- Which options (e.g. internet access, information material, prenatal classes) do women have to inform themselves or exchange information about pregnancy in a language they are familiar with?
- Whom can a pregnant woman contact for acute problems and emergencies? If there is no formal contact person, what do women usually do in case of emergencies?
- Which options does a pregnant woman with pregnancy issues (e.g. contractions or back pains) have, so she can be alone?

5. Care during birth:

- How is the transport to the place of child birth organised (public transport, car)?
- Is the pregnant woman accompanied by someone? If yes, by whom? (Partner, friend, acquaintance, relative). In case of an emergency: availability of ambulances? Who covers the cost of an ambulance?

For peer review only - http://bmjopen.bmj.com/site/about/guidelines.xhtml

6. Protection from Violence in the camp/Asylum Centre

- Does your centre have a violence protection concept?
- How are women protected from gender-based violence and sexual assault/harassment?
- Whom can affected women turn to if they feel harassed? Are there any administrative hurdles?
- What supervision, debriefing, or counselling is available for health professionals who treat survivors of violence?

Thank you very much for your time!

For peer review only - http://bmjopen.bmj.com/site/about/guidelines.xhtml
